# Salt tolerance evaluation and mini-core collection development in *Miscanthus sacchariflorus* and *M. lutarioriparius*

**DOI:** 10.3389/fpls.2024.1364826

**Published:** 2024-03-05

**Authors:** Yanmei Tang, Shicheng Li, Dessireé Zerpa-Catanho, Zhihai Zhang, Sai Yang, Xuying Zheng, Shuai Xue, Xianyan Kuang, Mingxi Liu, Xiong He, Zili Yi, Liang Xiao

**Affiliations:** ^1^ College of Bioscience and Biotechnology, Hunan Agricultural University, Changsha, Hunan, China; ^2^ Department of Crop Sciences, University of Illinois at Urbana-Champaign, Champaign, IL, United States; ^3^ Institute for Sustainability, Energy, and Environment, University of Illinois at Urbana-Champaign, Champaign, IL, United States; ^4^ Orient Science & Technology College of Hunan Agricultural University, Changsha, Hunan, China; ^5^ Department of Biological and Environmental Sciences, Alabama A&M University, Huntsville, AL, United States; ^6^ Department of Grassland Science, College of Agronomy, Hunan Agricultural University, Changsha, Hunan, China; ^7^ Hunan Heyi Crop Science Co., Ltd., Changsha, Hunan, China

**Keywords:** *Miscanthus sacchariflorus*, *Miscanthus lutarioriparius*, seedling stage, salt tolerance, comprehensive evaluation, ion homeostasis, core collection

## Abstract

Marginal lands, such as those with saline soils, have potential as alternative resources for cultivating dedicated biomass crops used in the production of renewable energy and chemicals. Optimum utilization of marginal lands can not only alleviate the competition for arable land use with primary food crops, but also contribute to bioenergy products and soil improvement. *Miscanthus sacchariflorus* and *M. lutarioriparius* are prominent perennial plants suitable for sustainable bioenergy production in saline soils. However, their responses to salt stress remain largely unexplored. In this study, we utilized 318 genotypes of *M. sacchariflorus* and *M. lutarioriparius* to assess their salt tolerance levels under 150 mM NaCl using 14 traits, and subsequently established a mini-core elite collection for salt tolerance. Our results revealed substantial variation in salt tolerance among the evaluated genotypes. Salt-tolerant genotypes exhibited significantly lower Na^+^ content, and K^+^ content was positively correlated with Na^+^ content. Interestingly, a few genotypes with higher Na^+^ levels in shoots showed improved shoot growth characteristics. This observation suggests that *M. sacchariflorus* and *M. lutarioriparius* adapt to salt stress by regulating ion homeostasis, primarily through enhanced K^+^ uptake, shoot Na^+^ exclusion, and Na^+^ sequestration in shoot vacuoles. To evaluate salt tolerance comprehensively, we developed an assessment value (D value) based on the membership function values of the 14 traits. We identified three highly salt-tolerant, 50 salt-tolerant, 127 moderately salt-tolerant, 117 salt-sensitive, and 21 highly salt-sensitive genotypes at the seedling stage by employing the D value. A mathematical evaluation model for salt tolerance was established for *M. sacchariflorus* and *M. lutarioriparius* at the seedling stage. Notably, the mini-core collection containing 64 genotypes developed using the Core Hunter algorithm effectively represented the overall variability of the entire collection. This mini-core collection serves as a valuable gene pool for future in-depth investigations of salt tolerance mechanisms in *Miscanthus*.

## 1 Introduction

Salinity is a significant abiotic stress factor that inhibits plant growth and reduces crop yield (Munns and Gilliham, 2015). Soil salinization is a devastating global environmental issue, affecting over 833 million hectares and accounting for more than 8.7% of the world’s land area (FAO, 2021). In China, the distribution of saline land is extensive, with approximately 99.13 million hectares located mainly in northern, northwestern, northeastern, and coastal areas (Wu et al., 2019). Saline land is generally unsuitable for most crops and leads to reduced production and plant mortality. There is an urgent global ecological need to find practical approaches to improve and utilize these salty lands (Wang et al., 2019). The most promising strategy is screening and developing salt-tolerant crop species and varieties (Ashraf et al., 2012).

Plant response to salt stress is a complex genetic and physiological mechanism controlled by multiple quantitative trait loci (QTL) (Flowers, 2004). Salt stress induces osmotic pressure, ion toxicity, and nutritional imbalances, which reduce cell growth and alter metabolite levels (Munns and Tester, 2008). Higher plants have developed various adaptive mechanisms in response to salt stress, which are generally categorized into three groups: tolerance to osmotic stress, Na^+^ exclusion through leaves, and tissue tolerance (Munns, 2005). Under salt stress, plants regulate osmotic pressure through the synthesis of organic regulators and accumulation of inorganic ions. Among these ions, K^+^, Na^+^, and Cl^-^ are crucial for 80-95% of cellular osmoregulation (Munns, 2005). K^+^, in particular, is essential for maintaining vital cellular functions and has been emphasized for the critical role it plays in plant salt tolerance (Cuin et al., 2008). Additionally, plants possess mechanisms for external Na^+^ exclusion or ion segregation within cells (with ions accumulating in vesicles) to maintain ion homeostasis and stable plant growth under salt stress. Plant roots activate specific ion transporters, such as HKT (high-affinity K^+^ transporters), NHX (Na^+^/H^+^ antiporters), SOS (salt overly sensitive genes), HAK (high-affinity K^+^), potassium channels (AKT), and H^+^ pumping, to facilitate Na^+^ transport, compartmentation, or elimination under salt stress (Zhu, 2016).

*Miscanthus* is a perennial, rhizomatous, tall C4 grass that has been deemed a promising energy crop and is currently being developed to produce lignocellulosic biomass as a sustainable alternative to fossil fuels and as an eco-industrial crop (Shavyrkina et al., 2023). Growing *Miscanthus* on marginal land for biomass production could contribute to food security and efficient land use (Xue et al., 2016). Additionally, it has the potential to enhance soil carbon sequestration with long-term benefits for the recovery of marginal land by improving soil structure and fertility (Xu et al., 2021). *M. lutarioriparius* and *M. sacchariflorus* are closely related subspecies that are distributed in different habitats belonging to Poaceae, *Miscanthus* Anderson. *M. sacchariflorus*, characterized by high genetic diversity and adaptation (Sun et al., 2010), can be an alternative for ecological landscape restoration in coastal saline areas (Sun and Chen, 2015). Furthermore, *M. lutarioriparius*, a species endemic to Central China, exhibits the highest biomass production (Sun et al., 2010). Studies have shown that the seeds of *M. sacchariflorus* and *M. lutarioriparius* are more salt-tolerant than those of *M. sinensis* (Zheng et al., 2015). On marginal land (salinity level of 2.7 dS/m) in the Yellow River Delta, *M. sacchariflorus* demonstrated stable yields, whereas the biomass yield of *M. lutarioriparius* surpassed that of switchgrass (Zheng et al., 2019). Thus, *M. sacchariflorus* and *M. lutarioriparius* are biomass crops with a high potential for sustainable production in saline soils (Zheng et al., 2022). However, further research is required to fully understand the salt tolerance mechanisms of *M. sacchariflorus* and *M. lutarioriparius*. Previous evaluations of the salt tolerance of the seeds and seedlings of both species were performed using small sample sizes (Zong et al., 2013; Sun and Chen, 2015; Zheng et al., 2015; Chen et al., 2017; Duan et al., 2018). Similarly, transcriptomic approaches have shed light on some responsive genes in *M. lutarioriparius* under long-term salt stress. However, these represent only a fraction of the overall mechanisms underlying salt tolerance in *M. lutarioriparius* (Song et al., 2017; Wang et al., 2019; Yu et al., 2023). Therefore, the genetic diversity and mechanisms underlying salt tolerance in *M. sacchariflorus* and *M. lutarioriparius* remain largely unknown and require further investigation.

Studying the mechanism of salt tolerance using a large number of genotypes in *M. sacchariflorus* and *M. lutarioriparius* is time-consuming, labor-intensive, and expensive. To improve efficiency, it is necessary to establish a core library of salt tolerance that captures the entire range of genetic variability with minimal redundancy, by reducing the size and increasing the diversity of the germplasm set. This core set can provide a starting point for enhancing genetic gains and employing phenomic and genomic tools in less time (Tripathi et al., 2022). An economical, time-effective, and labor-saving approach for studying physiological or molecular response mechanisms using phenotypic traits of the core collection or trait-marker associations has been widely used (Guo et al., 2022; Wang et al., 2022).

In this study, we assessed the salt tolerance of 318 genotypes of *M. sacchariflorus* and *M. lutarioriparius* in a hydroponic system using 14 salt-tolerance traits. The objectives of this study were: (1) to determine the optimal salt concentration for evaluating salt tolerance in *M. sacchariflorus* and *M. lutarioriparius*; (2) to explore the genetic diversity and physiological mechanisms of salt tolerance in these species; (3) to develop a mathematical evaluation model for studying salt tolerance in *M. sacchariflorus* and *M. lutarioriparius*; and (4) to establish a core collection for salt tolerance in *M. sacchariflorus* and *M. lutarioriparius*. This study provides a better understanding of salt-tolerant *Miscanthus* variety breeding and improvement, as well as the optimum utilization of marginal lands.

## 2 Materials and methods

### 2.1 Plant materials

**Figure 1:**
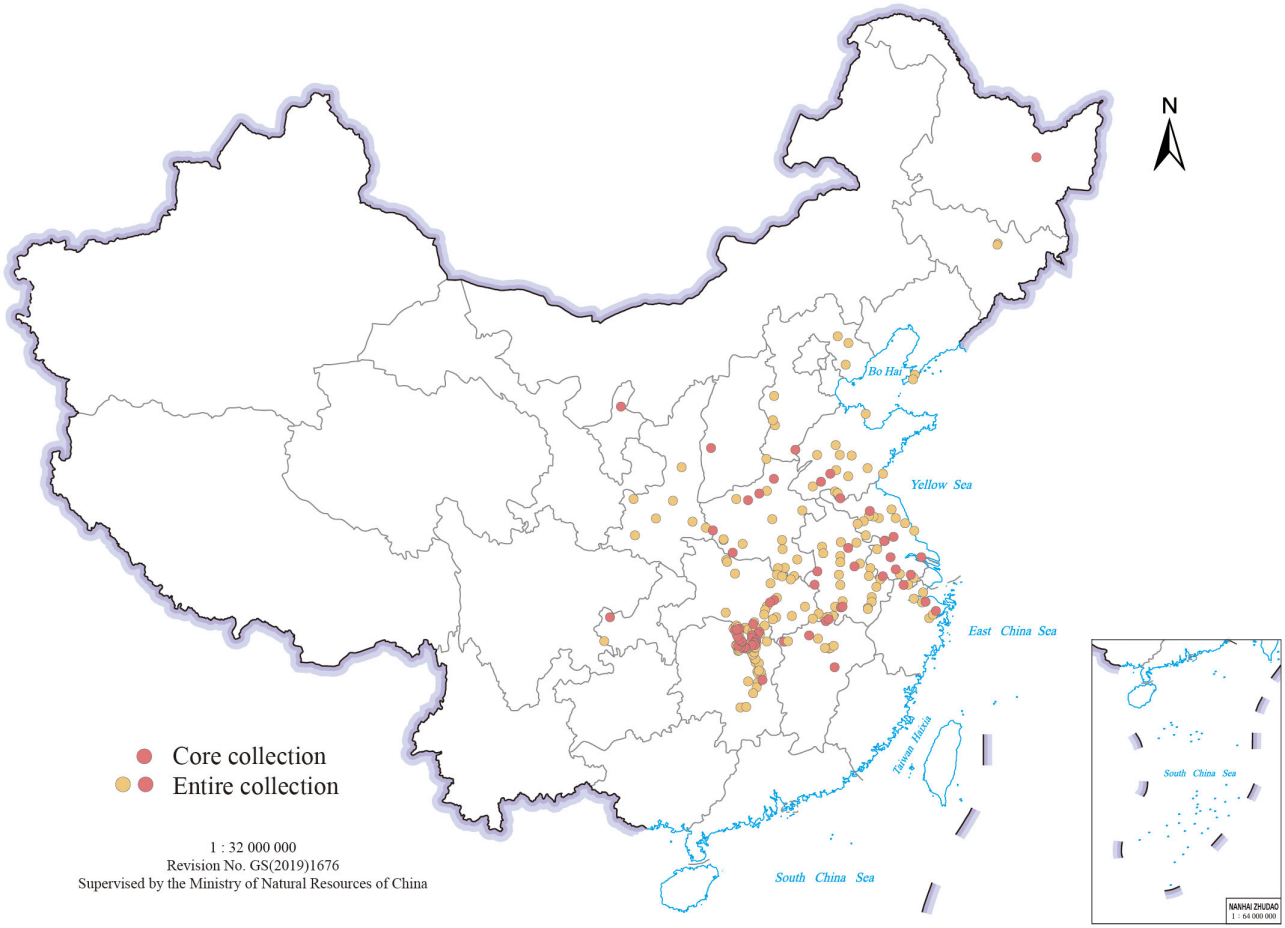
Collection sites of 318 *M. sacchariflorus* and *M. lutarioriparius* genotypes (excluding *M.* × *giganteus*) from different regions of China.

The study material comprised 318 *Miscanthus* genotypes, including 230 *M. lutarioriparius*, 87 *M. sacchariflorus*, and one *M.* × *giganteus* (Mxg), a natural allotriploid hybrid between *M. sacchariflorus* and *M. sinensis*. The genotypes were collected from a range of regions in China and cultivated in *Miscanthus* Germplasm Nursery of Hunan Agricultural University (N28.18°, E113.07°) in Changsha, Hunan, China. **Figure 1** and **Supplementary Table S1** show the geocoordinates of each collection site mapped using geographic information system (GIS) tools with the Krasovsky_1940 datum and geographic projection system. For each genotype, clones were subjected to vegetative propagation by dividing the rhizomes.

### 2.2 Determination of optimal salt stress concentration

A preliminary experiment was performed to determine the optimal salt-stress concentration. Ten genotypes (M87, M112, M129, M164, M228, M229, M245, M253, M275, and Mxg) were randomly selected from the 318 genotypes and used to determine the optimal salt concentration across a range of NaCl concentrations (0, 100, 150, 200, and 250 mM). To prepare the plant materials, rhizomes of each genotype were divided into small cuttings, with each cutting carrying 1-2 buds (approximately eight cuttings per treatment), and placed into plug trays filled with peat soil. After one month of growth in the greenhouse, plants of uniform height and growth were selected for each genotype. The plants were transferred to a hydroponic system following the removal of the attached soil and were subjected to a one-week acclimation period. The hydroponic system comprised a 24-hole floating planting plate (4 cm in diameter per hole), measuring 60 × 40 × 3 cm. This plate, which held a planting plug and a planting basket, was placed within a square plastic tray (61 × 42 × 9.5 cm) designed to accommodate up to 24 plants. A half-strength modified version of Hoagland’s solution was added to the square trays for growth support and was replaced every five days. Each container included eight genotypes in three replicates, totaling 24 plants. After one week of acclimation, NaCl was incrementally added to the nutrient solution at daily increments of 20, 30, 40, and 50 mM until the desired corresponding concentrations of 100, 150, 200, and 250 mM were reached, except for the 0 mM NaCl control. The hydroponic salt stress experiments were conducted in a growth chamber set at a constant temperature of 25°C, a relative humidity of 70%, and a light intensity of 650 μmol·m^-2^·s^-1^ (16 h light/8 h dark).

After 17 days of salt treatment, the plants from both the salt and control treatments were evaluated for 14 traits (**Table 1**): shoot growth rate (GR), leaves increased number (NIL), leaf expansion rate (LER), leaf senescence scale (Sen), shoot water content (SWC), root water content (RWC), shoot Na^+^ concentration (SNC), root Na^+^ concentration (RNC), the ratio of shoot Na^+^ concentration to root Na^+^ concentration (SN/RN), shoot K^+^ concentration (SKC), root K^+^ concentration (RKC), the ratio of shoot K^+^ concentration to root K^+^ concentration (SK/RK), the ratio of shoot K^+^ concentration to shoot Na^+^ concentration (SK/N), and the ratio of root K^+^ concentration to root Na^+^ concentration (RK/N).

**Table 1:** Description of abbreviations.

Code	Descriptors	Code	Descriptors
GR	Shoot growth rate	SKC	Shoot K^+^ concentration
GR_CK	Shoot growth rate under control	RKC	Root K^+^ concentration
GR_S	Shoot growth rate under 150 mM NaCl	SK/RK	The ratio of shoot K^+^ concentration to root K^+^ concentration
RGR	Salt-tolerance index of shoot growth rate	SK/N	The ratio of shoot K^+^ concentration to shoot Na^+^ concentration
NIL	Leaves increased number	RK/N	The ratio of root K^+^ concentration to root Na^+^ concentration
NIL_CK	Leaves increased number under control	STI	Salt tolerance index
NIL_S	Leaves increased number under 150 mM NaCl	SII	Salt-injury index
RNIL	Salt-tolerance index of leaves increased number	DAS	Day after starting the stress treatment
LER	Leaf expansion rate	MFV	Membership function value
LER_CK	Leaf expansion rate under control	HST	Highly salt tolerant
LER_S	Leaf expansion rate under 150 mM NaCl	ST	Salt tolerant
RLER	Salt-tolerance index of leaf expansion rate	MST	Moderately salt tolerant
Sen	Leaf senescence scale	SS	Salt sensitive
SWC	Shoot water content	HSS	Highly salt sensitive
RWC	Root water content	CR%	Coincidence rate of range between the entire and core collections
SNC	Shoot Na^+^ concentration	VR%	Variable rate between the entire and core collections
RNC	Root Na^+^ concentration	VD%	Variance difference percentage between the entire and core collections
SN/RN	The ratio of shoot Na^+^ concentration to root Na^+^ concentration	MD %	Mean difference percentage between the entire and core collections

The salt tolerance index (STI) for GR, NIL, and LER was calculated for each as the ratio of the value of stressed plants to the value of the control plants. For instance, the relative GR (RGR) was calculated as RGR = GR under salt stress/GR under control conditions. These ratios are referred to as RGR, Relative NIL (RNIL), and Relative LER (RLER), respectively. Additionally, salt-injury index (SII) is defined as SII = 1-STI, which quantifies the degree of injury from salt stress (Wu et al., 2019). The SII for GR, NIL and LER were calculated for the 10 genotypes at each concentration. The optimum stress concentration of NaCl was determined at the level where the SII was 50% of the control’s value and the diversity of Sen was the greatest.

### 2.3 Main experiment design

Following the above-mentioned methods, plants derived from a pool of 318 genotypes were vegetative propagation, washed, and transferred to a hydroponic system in a growth chamber for evaluation. Each treatment had three biological replicates and was treated with the optimal NaCl concentration (150 mM NaCl) and 0 mM NaCl (control). The growth chamber conditions were identical to those used in the preliminary experiment. After four days of acclimation in the hydroponics system, NaCl was added to the stress treatment group at 75 mM daily increments until the final concentration reached 150 mM NaCl. Treatments were carried out for 17 d, during which GR, NIL, and LER were determined under control and salt stress conditions, and STI was calculated for these traits (RGR, RNIL, and RLER). In addition, Sen, SWC, RWC, SNC, RNC, SN/RN, SKC, RKC, SK/RK, SK/N, and RK/N were determined under the salt treatment. The experiment was repeated three times.

### 2.4 Assessment of growth traits

Plant height, leaf expansion, and leaf number were recorded for all plants under control and saline conditions. Plant height (cm) was measured from the base of the plant to the tip of the tallest leaf on the second day after starting the stress treatment (DAS) and at 7 and 17 DAS. The GR, NIL, LER, Sen, SWC, and RWC were assessed according to the protocol described by Chen et al. (2017). GR was evaluated as the daily increase in height, with the unit of centimeters per day (cm/day); it was calculated as the mean of the daily increase in height between 7 and 2 DAS and between 17 and 2 DAS. Similarly, NIL (leaf) was obtained by subtracting the number of leaves at 1 DAS from the number of leaves at 17 DAS. For leaf expansion measurements, the youngest leaf of each plant was marked at 1 DAS and its length measured at 2, 7, and 12 DAS. LER was evaluated as the mean of the daily leaf expansion between 7 and 2 DAS and between 12 and 2 DAS (cm/day). Sen was measured by visually scoring all leaves of each plant under salt stress at 17 DAS, using a 1 to 9 scale according to the percentage of senesced area (1 = no senescence, 3 = 1-30% senesced area, 5 = 30-60% senesced area, 7 = 60-90% senesced areas, 9 = >90% senesced area). At harvest (17 DAS), all plants from the control and salt treatment groups were thoroughly washed, blotted dry, cut, and separated into shoots and roots. The fresh weights of the shoots and roots were then separately measured immediately. Subsequently, both plant parts were dried separately in a forced- air oven at 80°C for 3-4 days, and their dry weights were recorded to calculate the SWC (%) and RWC (%).

### 2.5 Measurement of Na^+^ and K^+^ concentrations

To determine the ion concentrations in the shoots and roots, three replicate samples of either shoots or roots for each genotype were pooled after the dry weights were measured. The samples were then processed by shearing and grinding to a fine powder using a sample grinder, followed by sieving with a 0.15 mm sieve. A 0.1 g sample of this dry powder was digested on a graphite digester using the H_2_SO_4_-H_2_O_2_ digestion method. Distilled water was added to the digested solution to obtain a final volume of 50 mL. The sample solutions were then filtered through a filter with a pore size of 0.22 µm, and the filtered solutions were diluted 160-fold for shoot samples and 100-fold for root samples before the Na^+^ and K^+^ content was assessed for each genotype’s root and leaf samples using a flame atomic absorption spectrophotometer (AA-7000, SHIMADZU, Japan).

### 2.6 Salt tolerance evaluation

The salt tolerance of *M. sacchariflorus* and *M. lutarioriparius* was evaluated by calculating the membership function value (MFV) of STI and traits such as RGR, RNIL, RLER, Sen, SWC, RWC, SNC, RNC, SN/RN, SKC, RKC, SK/RK, SK/N, and RK/N using a fuzzy comprehensive evaluation method. The MFV of salt tolerance was calculated using the following equation (Wassie et al., 2019; Weng et al., 2021; Wang et al., 2023):


(1) $${\text{F}}_{\text{ij}}=\;\left({\text{X}}_{\text{ij}}-{\text{ X}}_{\text{jmin}}\right)/\left({\text{X}}_{\text{jmax}}-{\text{ X}}_{\text{jmin}}\right)$$

(2) $${\text{F}}_{\text{ij}}=\;1\;-\;\left({\text{X}}_{\text{ij}}-{\text{ X}}_{\text{jmin}}\right)/\left({\text{X}}_{\text{jmax}}-{\text{ X}}_{\text{jmin}}\right)$$

(3) $${\text{D}}_{\text{i}}=\frac{1}{\text{n}}\sum_{\text{j}=1}^{\text{n}}{\text{F}}_{\text{ij}}$$

Where F_ij_ is the MFV of indicator (j) for genotype (i) for salt tolerance. X_ij_ is the value of indicator (j) for genotype (i). X_jmax_ and X_jmin_ are the maximum and minimum values of the indicator (j), respectively. The membership function reflects the positive correlation between a particular indicator variable and salt stress, as expressed in Equation 1, whereas Equation 2 expresses a negative correlation. D_i_ is the mean MFV of the 14 salt tolerance traits of genotype (i) by Equation 3 for salt tolerance. Higher values of D indicate higher salt tolerance.

### 2.7 Hierarchical cluster analysis

Hierarchical cluster analysis based on the Euclidean distance of D values was also used to evaluate salt tolerance. Salt tolerance was clustered into five different levels: highly salt tolerant (HST), salt tolerant (ST), moderately salt tolerant (MST), salt sensitive (SS), and highly salt sensitive (HSS).

Multiple regression analysis was performed on the 303 genotypes’ D values (taking as dependent variable Y) and indicator values (taking as independent variable X_i_). A mathematical evaluation model for salt tolerance was established as follows: Y = μ+β_1_X_1_+β_2_X_2_+β_3_X_3_+β_4_X_4_+β_5_X_5_+β_6_X_6_+β_7_X_7_, where Y is the D_i_, X_1_ is SNC, X_2_ is RNIL, X_3_ is RWC, X_4_ is RGR, X_5_ is RKC, X_6_ is Sen, X_7_ is RNC, β is the B of unstandardized coefficient, and μ is constant (random error). For model validation, another 15 genotypes were used, with three genotypes selected from each of the five salt tolerance levels.

### 2.8 Development and validation of mini-core collection

A mini-core collection was developed using 14 traits of salt tolerance (RGR, RNIL, RLER, Sen, SWC, RWC, SNC, RNC, SN/RN, SKC, RKC, SK/RK, SK/N, and RK/N) and the distance-based Core Hunter approach. Gower’s distance measure (Gower, 1971) was employed in the CH3 method (De Beukelaer et al., 2018) to calculate the pairwise distances between genotypes as entry-to-nearest-entry (E-NE) and access-to-nearest-entry (A-NE). The core set, which accounted for 20% of the entire collection, was generated by optimizing both E-NE (maximizing) and A-NE (minimizing) with equal weighting. The optimization process used the default parallel tempering search algorithm, which was terminated when no improvement was observed for 30 s.

To compare the mean, variance, median, and representativeness of the entire and core collections for the 14 traits, we applied the Newman-Keuls procedure (Newman, 1939; Keuls, 1952), Levene’s test (Levene, 1960), Wilcoxon rank-sum non-parametric test (Wilcoxon, 1945), and the Shannon-Weaver diversity index (Shannon and Weaver, 1949), respectively. In addition, we assessed the quality of the developed mini-core collection using various criteria proposed by Hu et al. (2000) and Kim et al. (2007). These criteria included the estimation of the mean difference percentage (MD %), variance difference percentage (VD %), coincidence rate of range (CR %), and variable rate (VR %) between the entire and core collections. The mini core collection was considered well represented by the entire collection if MD % was below 20%, VD % and VR % were sufficiently large, and CR % exceeded 80% (Hu et al., 2000). The similarity in genetic traits between the entire and core collections was also compared using correlation analysis. The contributions of different descriptor traits to multivariate polymorphisms and conservation in the core collection were assessed using principal component analysis (PCA).

### 2.9 Statistical analysis

Data are presented as means. The statistics and summarization of the data were conducted using Excel 365. All subsequent data analysis and visualization were performed by R 4.3.0, with the exception of generating the distribution map of germplasm using GIS. Specifically, the core collection tests were performed using the *EvaluateCore* package in R (Aravind et al., 2022).

## 3 Results

### 3.1 Determination of optimal salt concentration

**Figure 2:**
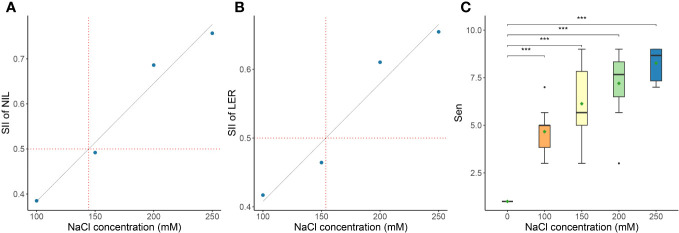
Determination of the optimal NaCl concentration for evaluating salt tolerance. The NaCl concentration of the salt-injury index (SII) is 0.5 of leaves increased number (NIL) **(A)**, leaf expansion rate (LER) **(B)** of ten *Miscanthus* genotypes under different NaCl concentrations, as well as the effect on leaf senescence scale (Sen) (significance of the t-test: ^***^ *P*<0.001) **(C)**. Data in the figure are means of ten *Miscanthus* genotypes for each trait under each concentration of NaCl.

We evaluated the GR, NIL, LER, Sen, SWC, RWC, SNC, RNC, SN/RN, SKC, RKC, SK/RK, SK/N, and RK/N of the genotypes at 17 DAS, as detailed in **Supplementary Table S2**, **Supplementary Figure S1**. The SII of GR, NIL, and LER were calculated from the data in **Supplementary Tables S2 and S3**. Linear regression analysis was conducted on the mean values of the three SII across the ten genotypes. The results indicated that according to the linear regression fitting, the SII for NIL would decrease to 50% of the control’s value at a NaCl concentration of 144.48 mM NaCl (**Figure 2A**), and the SII for LER would decrease to 50% of the control’s value at the predicted 153.70 mM (**Figure 2B**). Notably, Sen exhibited considerable variability under 150 mM NaCl treatment, indicating its suitability as a criterion for evaluating salt tolerance across different genotypes (**Figure 2C**). Therefore, 150 mM NaCl was used for the evaluation of the salt tolerance for the 318 genotypes in the present study.

### 3.2 Phenotypic variations for salt tolerance-related traits

**Figure 3:**
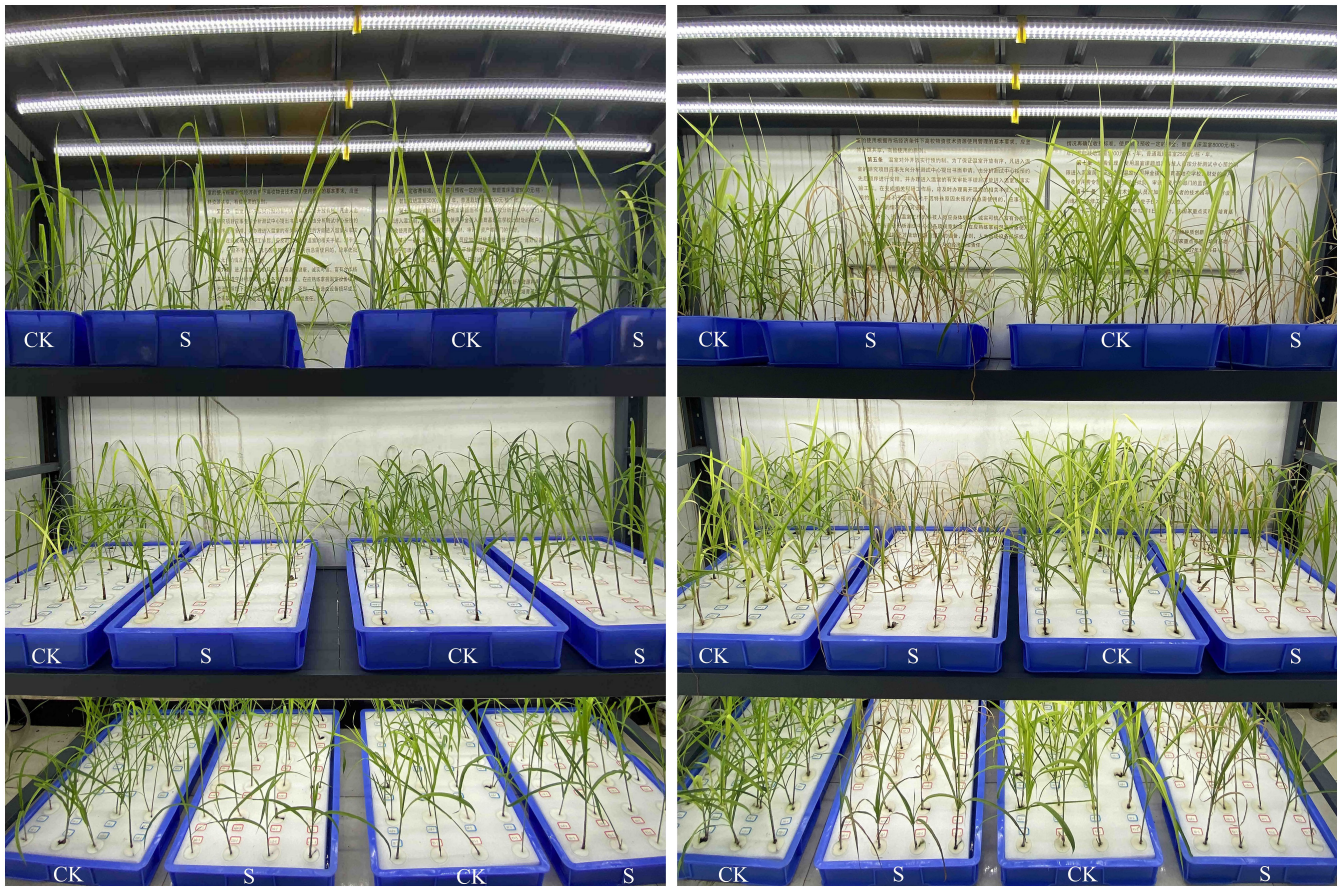
Plants were subjected to 150 mM NaCl (S) and control (CK) conditions for 0 (left panel) and 17 days (right panel). Each group of two adjacent hydroponic containers, from left to right, was used to represent the CK and S conditions, respectively. The position of each plant in these two hydroponic containers was matched one-to-one.

Salt tolerance of the 318 genotypes at the seedling stage was evaluated using 150 mM NaCl, as detailed in **Supplementary Table S4** and **Figure 3**. Descriptive statistics for the salt tolerance traits, including RG, RGR, NIL, RNIL, LER, RLER, Sen, SWC, RWC, SNC, RNC, SN/RN, SKC, RKC, SK/RK, SK/N, and RK/N, are presented in **Table 2** and **Supplementary Figure S2**. Continuous variation was observed across all traits with approximately normal distributions, as seen in **Supplementary Figure S3**. The 318 genotypes exhibited a wide variation in response to the 150 mM NaCl treatment. A Kruskal-Wallis test applied to salt tolerance-related traits indicated significant differences (*P*<0.001) among genotypes, suggesting that genetic effects explained a large proportion of the phenotypic variance. Significant differences in GR, NIL, and LER between the control and salt treatment (*P*<0.001) were also noted (**Supplementary Figure S2**). Furthermore, to account for background differences, salt tolerance indices of GR, NIL, and LER (RGR, RNIL, and RLER, respectively) were used to evaluate the salt stress response in *M. sacchariflorus* and *M. lutarioriparius*. RGR, RNIL, and RLER showed variations ranging from 0.03 to 1.77, 0.00 to 0.94, and 0.19 to 1.09, respectively, with RGR exhibiting the most significant coefficient of variation at 53%. The water content of the plants was affected by salt stress, leading to SWC varying from 4.37% to 28.78% and RWC from 3.57% to 22.62%. Sen exhibited a mean value of 6.33 with a range of variability from 3.67 to 8.78 across different genotypes, highlighting the diverse response of *M. sacchariflorus* and *M. lutarioriparius* genotypes to salt stress.

**Table 2:** Descriptive statistics of all traits for 318 *M. sacchariflorus* and *M. lutarioriparius* genotypes.

Traits	Min	Max	Mean	SE	CV	Skewness	Kurtosis
GR_CK (cm/day)	0.06	0.96	0.39	0.01	0.39	0.64	0.42
GR_S (cm/day)	0.01	0.59	0.16	0.01	0.55	1.30	3.14
RGR	0.03	1.77	0.49	0.01	0.53	1.09	2.29
NIL_CK (leaf)	0.78	3.33	2.30	0.02	0.18	-0.39	0.41
NIL_S (leaf)	0.00	2.11	1.06	0.02	0.34	0.10	-0.04
RNIL	0.00	0.94	0.47	0.01	0.33	0.18	0.07
LER_CK (cm/day)	0.56	2.46	1.22	0.02	0.26	0.60	0.65
LER_S (cm/day)	0.17	1.63	0.69	0.01	0.35	0.86	1.12
RLER	0.19	1.09	0.61	0.01	0.28	0.19	-0.27
SWC (%)	4.37	28.78	15.57	0.25	0.29	0.42	-0.36
RWC (%)	3.57	22.62	11.10	0.20	0.32	0.56	-0.03
Sen	3.67	8.78	6.33	0.06	0.16	-0.06	-0.32
SNC (mg/g)	53.20	220.31	126.00	1.45	0.21	0.33	0.35
RNC (mg/g)	23.97	82.13	45.22	0.60	0.24	0.47	-0.20
SN/RN	1.17	5.68	3.13	0.04	0.24	0.11	0.16
SKC (mg/g)	44.32	104.75	68.39	0.45	0.12	0.22	0.88
RKC (mg/g)	12.83	39.50	26.20	0.30	0.21	0.15	-0.31
SK/RK	1.60	6.03	2.94	0.05	0.28	1.13	1.58
SK/N	0.33	1.33	0.59	0.01	0.26	1.51	3.49
RK/N	0.26	1.22	0.63	0.01	0.30	0.48	-0.06

SE, standard error; CV, coefficient of variation.

GR_CK, Shoot growth rate under control; GR_S, Shoot growth rate under 150 mM NaCl; RGR, salt-tolerance index of shoot growth rate; NIL_CK, leaves increased number under control; NIL_S, leaves increased number under 150 mM NaCl; RNIL, salt-tolerance index of leaves increased number; LER_CK, leaf expansion rate under control; LER_S, leaf expansion rate under 150 mM NaCl; RLER, salt-tolerance index of leaf expansion rate; Sen, leaf senescence scale; SWC, shoot water content; RWC, root water content; SNC, shoot Na^+^ concentration; RNC, root Na^+^ concentration; SN/RN, the ratio of shoot Na^+^ concentration to root Na^+^ concentration; SKC, shoot K^+^ concentration; RKC, root K^+^ concentration; SK/RK, the ratio of shoot K^+^ concentration to root K^+^ concentration; SK/N, the ratio of shoot K^+^ concentration to shoot Na^+^ concentration; RK/N, the ratio of root K^+^ concentration to root Na^+^ concentration.

**Figure 4:**
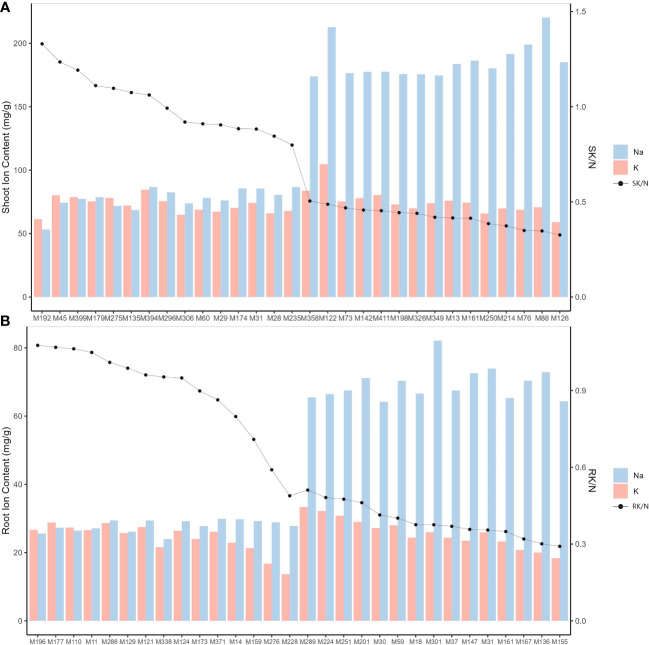
Na^+^ and K^+^ concentrations and K^+^/Na^+^ ratios in *M. sacchariflorus* and *M. lutarioriparius* under 150 mM NaCl. Ion concentrations for 15 genotypes with the highest shoot Na^+^ concentration (SNC) and 15 genotypes with the lowest shoot Na^+^ concentration **(A)**, and ion concentrations for 15 genotypes with the highest root Na^+^ concentration (RNC) and 15 genotypes with the lowest root Na^+^ concentration **(B)**. SK/N and RK/N are K^+^/Na^+^ ratios in shoots and roots, respectively.

The 318 genotypes showed significant differences (*P*<0.001) in their Na^+^ and K^+^ responses to salt stress (**Figure 4**, **Table 2**, **Supplementary Table S4**). Specifically, SNC ranged from 53.20 mg/g (in genotype M192) to 220.31 mg/g (in genotype M88), RNC ranged from 23.97 mg/g (in genotype M338) to 82.13 mg/g (in genotype M301), and SN/RN was notably high at 3.13, suggesting a greater accumulation of Na^+^ in the shoots of *M. sacchariflorus* and *M. lutarioriparius* under salt stress. In contrast, the range of K^+^ concentration was narrower; SKC was 44.32 mg/g (in genotype M19) - 104.75 mg/g (in genotype M122), and RKC was 12.83 mg/g (M203) - 39.50 mg/g (M349), but SK/RK also reached 2.94. Regarding the K^+^ to Na^+^ ratio, SK/N ranged from 0.33 (in genotype M126) to 1.33 (in genotype M192), and RK/N ranged from 0.26 (in genotype M123) to 1.22 (in genotype M405). Notably, a decline in SKC and RKC was not observed despite increased SNC, indicating the capacity of *M. sacchariflorus* and *M. lutarioriparius* to enhance K^+^ uptake under salt stress conditions. Additionally, among the 20 genotypes with the highest SNC, genotypes M283 and M349 exhibited Sen values lower than 7, specifically 6.56. Genotype M283 also displayed relatively high levels of RNIL, RLER, and SWC; M349 also exhibited relatively high RGR and SWC, and slightly higher average RNIL. These results suggest that these genotypes may utilize a tissue tolerance mechanism, possibly through the accumulation of Na^+^ in shoot vacuoles.

### 3.3 Correlation and principal component analysis of seedling traits under salt stress

**Figure 5:**
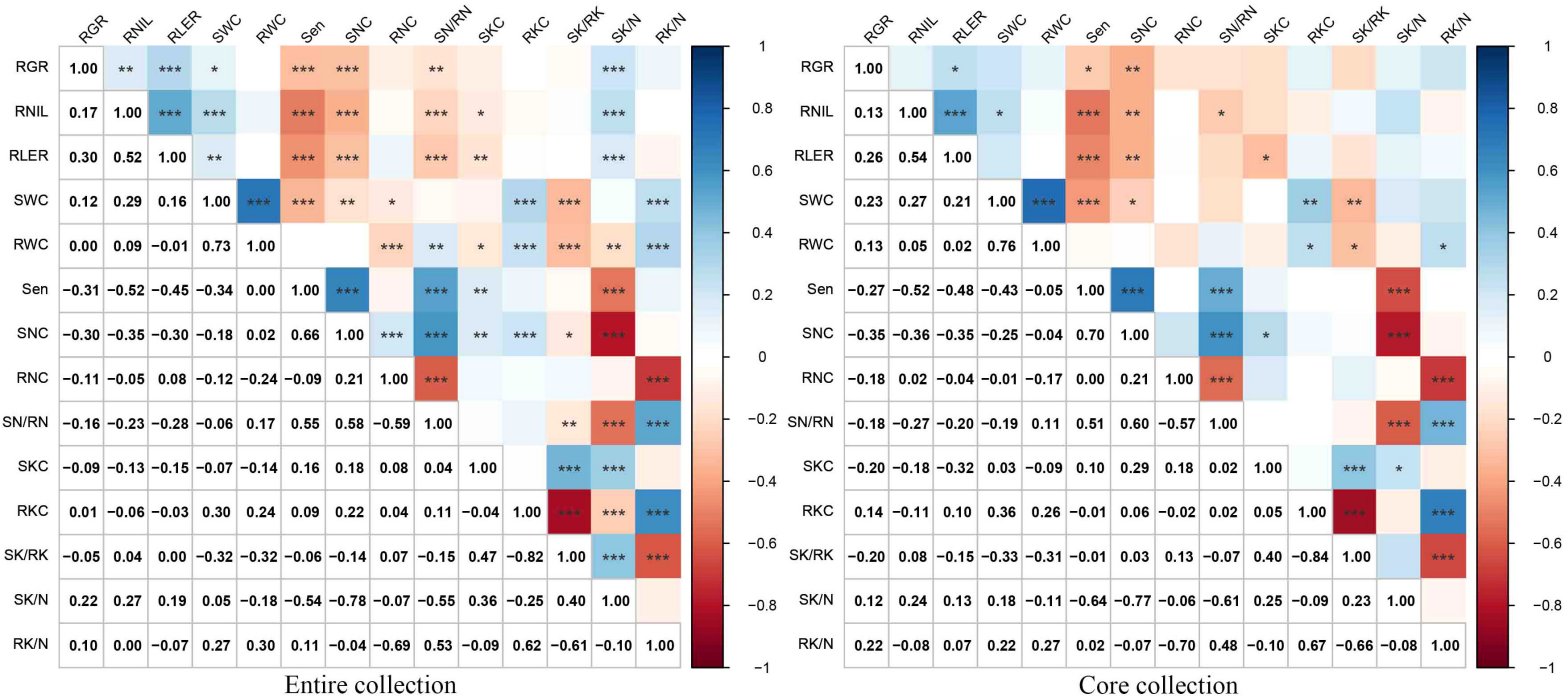
Pearson correlation analysis between 14 traits of the entire and core collections. *, **, and *** indicate significance at P<0.05, P<0.01, and P<0.001, respectively. Salt-tolerance index of leaves increased number (RNIL), salt-tolerance index of leaf expansion rate (RLER), leaf senescence scale (Sen), shoot water content (SWC), root water content (RWC), shoot Na^+^ concentration (SNC), root Na^+^ concentration (RNC), the ratio of shoot Na^+^ concentration to root Na^+^ concentration (SN/RN), shoot K^+^ concentration (SKC), root K^+^ concentration (RKC), the ratio of shoot K^+^ concentration to root K^+^ concentration (SK/RK), the ratio of shoot K^+^ concentration to shoot Na^+^ concentration (SK/N), and the ratio of root K^+^ concentration to root Na^+^ concentration (RK/N).

Correlation analyses were conducted to examine the relationships among various parameters under NaCl stress, including RGR, RNIL, RLER, Sen, SWC, RWC, SNC, RNC, SN/RN, SKC, RKC, SK/RK, SK/N, and RK/N (**Figure 5**). Significant positive correlations were found between RGR, RLER, RNIL, and SWC as well as between SKC, SK/RK, and SK/N. The highest positive correlation coefficient was observed between SWC and RWC (0.73), followed by SNC and Sen (0.66). Significant positive correlations were also noted between SN/RN and SNC (0.58), Sen (0.55), and RK/N (0.53). Notably, both SKC and RKC showed significant positive correlations with SNC. Conversely, significant negative correlations existed between Sen, SNC, and SN/RN with RGR, RNIL, RLER, and SK/N. The correlation coefficient between Sen and SK/N had the largest absolute value at -0.54, followed by that between Sen and RNIL at -0.52, and that between Sen and RLER at -0.45.

**Figure 6:**
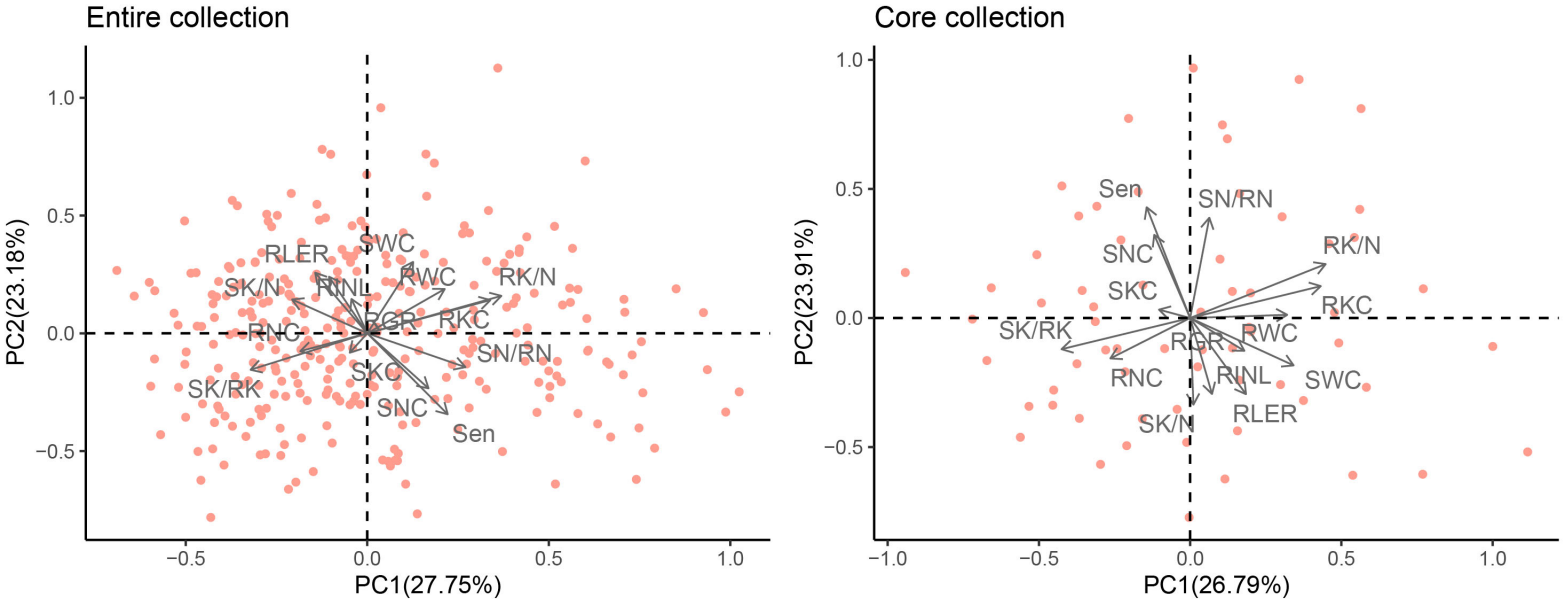
Principal component analysis plot of the entire and mini-core sets of *M. sacchariflorus* and *M. lutarioriparius* based on 14 traits. The angles captained by any of the two arrows less than 90° imply the two indices have a positive correlation, otherwise they represent a negative correlation between the two indices. Salt-tolerance index of shoot growth rate (RGR), salt-tolerance index of leaves increased number (RNIL), salt-tolerance index of leaf expansion rate (RLER), leaf senescence scale (Sen), shoot water content (SWC), root water content (RWC), shoot Na^+^ concentration (SNC), root Na^+^ concentration (RNC), the ratio of shoot Na^+^ concentration to root Na^+^ concentration (SN/RN), shoot K^+^ concentration (SKC), root K^+^ concentration (RKC), the ratio of shoot K^+^ concentration to root K^+^ concentration (SK/RK), the ratio of shoot K^+^ concentration to shoot Na^+^ concentration (SK/N), and the ratio of root K^+^ concentration to root Na^+^ concentration (RK/N).

PCA was used to synthesize these 14 normalized traits. Based on eigenvalues greater than one, five principal components (PCs) were extracted, which accounted for a cumulative variance contribution rate of 81.42% (**Table 3**, **Figure 6**). PC1 (27.75%) mainly reflected the effect of 150 mM NaCl stress on RK/N, RKC, SN/RN (positive loading), and SK/RK (negative loading), which are traits related to ion content. PC2 (23.18%) was significantly correlated with the growth traits of SWC, RLER, RNIL (positive loading), and Sen (negative loading) as well as the ion content-related traits of SNC (negative loading). PC3 (12.87%) was primarily associated with RNC, showing a significantly negative loading. PC4 (9.82%) included SWC and RWC, with positive loadings. Finally, PC5 (7.79%) mainly consisted of RLER with a positive loading.

**Table 3:** Principal component analysis of the entire and core sets showing the contributions of each trait to the variation in the germplasm.

Traits	Entire set					Core set				
	PC1	PC2	PC3	PC4	PC5	PC1	PC2	PC3	PC4	PC5
RGR	-0.02	0.06	0.02	-0.04	0.01	0.08	-0.06	-0.03	-0.02	0.00
RNIL	-0.04	**0.10**	0.01	0.00	0.06	0.03	**-0.13**	-0.03	0.00	0.09
RLER	-0.06	**0.11**	-0.02	-0.03	**0.12**	0.08	**-0.13**	-0.04	-0.07	**0.13**
SWC	0.05	**0.13**	-0.01	**0.10**	-0.02	**0.15**	-0.08	0.08	**0.12**	0.00
RWC	0.09	0.08	0.01	**0.13**	-0.01	**0.14**	0.01	0.07	**0.17**	0.03
Sen	0.09	**-0.14**	0.00	0.01	0.02	-0.06	**0.19**	0.03	0.00	0.02
SNC	0.07	**-0.10**	-0.05	0.03	0.05	-0.05	**0.14**	0.06	0.01	0.05
RNC	-0.08	-0.03	**-0.16**	0.02	-0.01	**-0.11**	-0.07	**0.21**	-0.05	0.03
SN/RN	**0.11**	-0.06	0.08	0.01	0.06	0.03	**0.17**	-0.08	0.04	0.05
SKC	-0.02	-0.03	0.02	0.01	-0.05	-0.04	0.01	0.03	0.04	-0.08
RKC	**0.14**	0.06	-0.09	-0.06	-0.04	**0.19**	0.05	**0.10**	-0.09	-0.05
SK/RK	**-0.14**	-0.07	0.08	0.05	0.00	**-0.19**	-0.05	-0.07	0.09	-0.01
SK/N	-0.09	0.06	0.05	-0.03	-0.08	0.00	**-0.15**	-0.04	0.01	**-0.12**
RK/N	**0.16**	0.07	0.07	-0.06	-0.02	**0.20**	0.09	-0.07	-0.03	-0.04
Eigenvalues	0.12	0.10	0.06	0.04	0.03	0.19	0.17	0.09	0.07	0.06
% of Variance	27.75	23.18	12.87	9.82	7.79	26.79	23.91	13.21	10.20	8.78
Cumulative %	27.75	50.93	63.80	73.62	81.42	26.79	50.70	63.91	74.11	82.90

Bold numbers indicate eigenvalues are significant ≥|0.1|.

RGR, salt-tolerance index of shoot growth rate; RNIL, salt-tolerance index of leaves increased number; RLER, salt-tolerance index of leaf expansion rate; Sen, leaf senescence scale; SWC, shoot water content; RWC, root water content; SNC, shoot Na^+^ concentration; RNC, root Na^+^ concentration; SN/RN, the ratio of shoot Na^+^ concentration to root Na^+^ concentration; SKC, shoot K^+^ concentration; RKC, root K^+^ concentration; SK/RK, the ratio of shoot K^+^ concentration to root K^+^ concentration; SK/N, the ratio of shoot K^+^ concentration to shoot Na^+^ concentration; RK/N, the ratio of root K^+^ concentration to root Na^+^ concentration.

### 3.4 Comprehensive evaluation of salt stress tolerance

**Figure 7:**
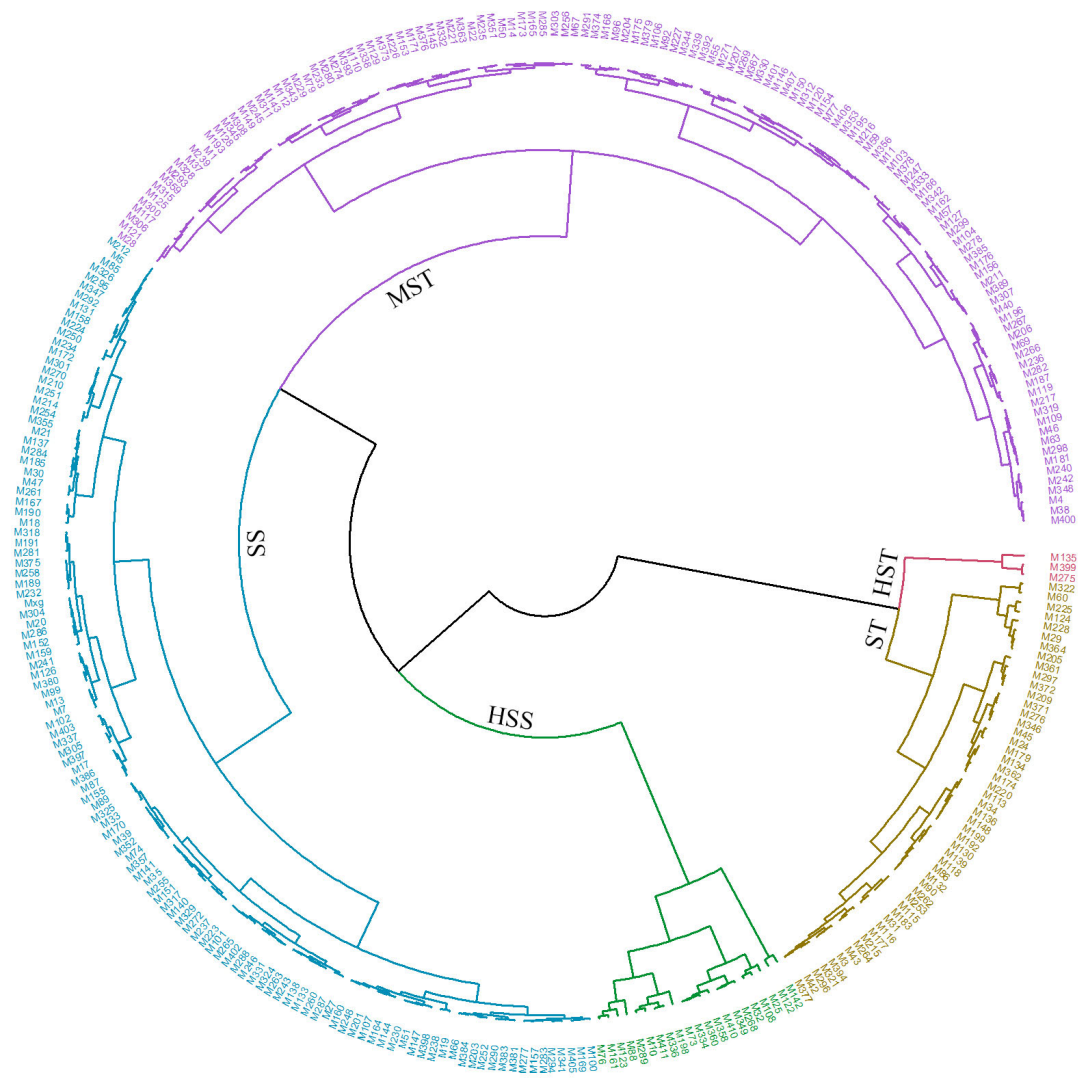
Hierarchical cluster analysis based on the Euclidean distance to evaluate the salt tolerance of 318 *M. sacchariflorus* and *M. lutarioriparius*. HST, highly salt tolerant; ST, salt tolerant; MST, moderately salt tolerant; SS, salt sensitive; and HSS, highly salt sensitive.

Based on the results of the pre-experiment for the ten genotypes and the subsequent correlation analysis, it was found that Sen, SNC, RNC, SN/RN, SKC, and RKC exhibited higher levels under salt stress than under the control conditions and that these traits showed a negative correlation with salt stress (**Supplementary Figure S1**, **Figure 5**). Consequently, Equation 2 was employed to calculate the MFV for Sen, SNC, RNC, SN/RN, SKC, and RKC, whereas Equation 1 was used for the remaining eight traits. The mean MFV (D value) was calculated for each genotype (see **Supplementary Table S5**). Hierarchical cluster analysis based on the D values was used to assess the salt tolerance of 318 *M. sacchariflorus* and *M. lutarioriparius* genotypes (**Figure 7**). Salt tolerance was classified into five levels at a Euclidean distance of 0.12: highly salt tolerant (HST), salt tolerant (ST), moderately salt tolerant (MST), salt sensitive (SS), and highly salt sensitive (HSS). Among the genotypes analyzed, three were classified as HST, 50 as ST, 127 as MST, 117 as SS, and 21 as HSS. Across these categories, a trend was observed whereas the level of salt tolerance decreased from HST to HSS, the values of RGR, RLER, and SK/N decreased, whereas Sen, SNC, RNC, and SN/RN exhibited an increasing trend. Notably, SNC was over two times as high in the HSS group (176.6 mg/g) compared to the HST (72.52 mg/g), as detailed in **Supplementary Table S6**. Genotypes classified in the higher salt tolerance categories exhibited superior growth characteristics, reduced leaf senescence, and lower Na^+^ content.

To screen and evaluate salt tolerance traits in *M. sacchariflorus* and *M. lutarioriparius*, the D values of 303 genotypes were used as a dependent variable and the values of the 14 traits as independent variables to develop the most predictive regression equation for salt tolerance. The random error term was 0.6895. The unstandardized coefficients for SNC, RNIL, RWC, RGR, RKC, Sen, and RNC were -0.0012, 0.1242, 0.0057, 0.0516, -0.0023, -0.0202, and -0.0010, respectively. The optimal regression equation is as follows: Y=0.6895-0.0012SNC+0.1242RNIL+0.0057RWC+0.0516RGR-0.0023RKC-0.0202Sen-0.0010RNC (R^2^ = 0.962, *P*<0.001) (**Table 4**).

**Table 4:** Multiple linear regression analysis of the comprehensive evaluation value of salt tolerance (D value) on seven traits.

	Unstandardized coefficients		Standardized coefficients		
Variables	B	Standard error	Beta	*t*	Sig.
(Constant)	0.6895	0.0108		63.58	0.00
SNC	-0.0012	0.0000	-0.4140	-25.48	0.00
RNIL	0.1242	0.0062	0.2689	20.17	0.00
RWC	0.0057	0.0002	0.2912	23.82	0.00
RGR	0.0516	0.0033	0.1884	15.63	0.00
RKC	-0.0023	0.0002	-0.1793	-14.90	0.00
Sen	-0.0202	0.0013	-0.2791	-16.06	0.00
RNC	-0.0010	0.0001	-0.1601	-12.39	0.00

SNC, shoot Na^+^ concentration; RNIL, salt-tolerance index of leaves increased number; RWC, root water content; RGR, salt-tolerance index of shoot growth rate; RKC, root K^+^ concentration; Sen, leaf senescence scale; RNC, root Na^+^ concentration.

To verify the predictability of the mathematical evaluation model for salt tolerance in *M. sacchariflorus* and *M. lutarioriparius*, three genotypes from each cluster were randomly selected and their corresponding Y values were calculated (**Table 5**). These results demonstrated that this formula can effectively evaluate the salt tolerance of *M. sacchariflorus* and *M. lutarioriparius* at the seedling stage. For instance, for genotype M411 which was classified as HSS, it had a Y value of 0.3167 (Y= 0.6895-0.0012*177.6243 + 0.1242*0.2667 + 0.0057*8.4277 + 0.0516*0.0350-0.0023*29.2614-0.0202*7.0000-0.0010*33.9501), which closely matched its D value of 0.317; for genotype M333 which was categorized as MST, it had a Y value of 0.4496, aligning well with its D value of 0.4513; for genotype M135 which was under the HST group, its Y value is 0.6504, which is very close to its D value of 0.6528. The close correspondence between the D and Y values indicates the reliability of our model and its applicability in predicting salt tolerance in *M. sacchariflorus* and *M. lutarioriparius* at the seedling stage.

**Table 5:** Verification of salinity tolerance values (Y value) from multiple regression analysis with the comprehensive evaluation value of salt tolerance (D value).

Category	Genotype	SNC (mg/g)	RNIL	RWC (%)	RGR	RKC (mg/g)	Sen	RNC (mg/g)	D value	Y value
HST	M135	68.5235	0.6722	10.6811	1.3153	21.8653	4.1111	35.7473	0.6528	0.6504
HST	M399	77.2951	0.5873	9.6401	0.4770	17.0181	4.7778	42.9012	0.6321	0.5707
HST	M275	71.7442	0.5595	7.4736	1.0119	15.0141	4.3333	42.2869	0.6307	0.6034
ST	M29	76.0937	0.7806	7.5559	1.0206	24.2159	3.6667	57.0360	0.5981	0.6041
ST	M372	109.2919	0.6699	19.1205	0.2283	24.4107	4.7778	44.9874	0.5663	0.5647
ST	M42	101.2471	0.6257	10.9623	0.7333	17.8049	5.2222	54.8780	0.5376	0.5447
MST	M235	86.6145	0.4393	6.5162	0.4582	20.1583	5.0000	53.5684	0.5143	0.5000
MST	M333	143.8789	0.5833	9.5685	0.4326	25.8235	5.6667	42.7207	0.4513	0.4496
MST	M166	128.6327	0.2922	15.0708	0.4771	29.3749	6.5556	43.3426	0.4477	0.4386
SS	M160	120.8193	0.4180	7.0272	0.4324	22.1833	7.2222	30.6579	0.4331	0.4312
SS	M397	135.6414	0.0000	6.0231	0.8124	23.4249	6.3333	42.5636	0.3975	0.3786
SS	M318	155.5016	0.4563	10.2639	0.4746	26.3178	7.8889	41.0854	0.3845	0.3816
HSS	M268	148.0509	0.0741	9.1441	0.4711	28.2644	7.0000	55.8626	0.3481	0.3352
HSS	M336	161.1194	0.2323	8.8758	0.1580	22.0328	7.6667	50.8735	0.3180	0.3273
HSS	M411	177.6243	0.2667	8.4277	0.0350	29.2614	7.0000	33.9501	0.3172	0.3167

HST, highly salt tolerant; ST, salt tolerant; MST, moderately salt tolerant; SS, salt sensitive; HSS, highly salt sensitive.

SNC, shoot Na^+^ concentration; RNIL, salt-tolerance index of leaves increased number; RWC, root water content; RGR, salt-tolerance index of shoot growth rate; RKC, root K^+^ concentration; Sen, leaf senescence scale; RNC, root Na^+^ concentration.

### 3.5 Development and validation of the mini-core collection

The mini-core collection displayed strong representativeness of the entire collection, as determined by the Core Hunter algorithm. Of the 318 genotypes used for the entire collection, 64 (20%) were selected to constitute the mini-core collection (**Supplementary Table S4**). The CR % was high for all studied traits except for RWC and RNC, which fell slightly below 80%. CR % values were 100% for Sen, SNC, and RK/N. The mean CR % was calculated to be 94.28% (**Table 6**). The VR % values for all studied traits were high, ranging from 101.32% for RWC to 175.87% for RGR, with an average VR % value of 141.16. The highest and lowest VD % values were observed for the RGR and RWC, respectively. The MD % analysis indicated a minimal difference (0.18-8.69%) between the mini-core and entire collections for all the traits. The Newman-Keuls test and Wilcoxon rank-sum test showed no significant differences in the means and medians between the entire and core collections for the studied traits. Furthermore, Levene’s test results indicated that there were no significant differences (*P*>0.05) between the core and entire collections for all traits except RGR, SN/RN, and RK/N, demonstrating that the central tendency and variability of phenotypic traits in the mini-core collection closely matched those in the entire collection. The Shannon-Weaver diversity index, which measures the diversity of individual phenotypic traits, ranged from 3.97 to 4.15 in the mini-core collection and from 5.63 to 5.76 in the entire collection, averaging 4.10 and 5.72, respectively. These results imply a moderate loss of diversity, about 28.32%, in the core collection, which could be attributed to missing genetic data.

**Table 6:** Evaluation parameters of the core collection.

Traits	CR %	VR %	VD %	MD %	Shannon-Weiner diversity index^a^	Newman-Keuls test	Levene’s test	Wilcoxon rank-sum test
RGR	99.97%	175.87%	43.14%	8.69%	3.97 (5.63)	0.22 ns	0.03^*^	0.59 ns
RNIL	91.18%	128.54%	22.20%	3.57%	4.09 (5.70)	0.43 ns	0.22 ns	0.43 ns
RLER	94.08%	137.70%	27.38%	2.38%	4.10 (5.72)	0.54 ns	0.08 ns	0.62 ns
SWC	92.25%	132.98%	24.80%	4.48%	4.10 (5.72)	0.30 ns	0.20 ns	0.24 ns
RWC	79.34%	101.32%	1.30%	5.60%	4.10 (5.71)	0.23 ns	0.79 ns	0.22 ns
Sen	100.00%	131.25%	23.81%	0.18%	4.14 (5.75)	0.94 ns	0.42 ns	0.90 ns
SNC	100.00%	142.09%	29.62%	0.30%	4.13 (5.74)	0.92 ns	0.21 ns	0.88 ns
RNC	79.75%	121.40%	17.63%	0.45%	4.13 (5.73)	0.89 ns	0.15 ns	0.94 ns
SN/RN	99.58%	157.12%	36.35%	0.64%	4.11 (5.73)	0.85 ns	0.03^*^	0.57 ns
SKC	94.49%	145.92%	31.47%	1.86%	4.15 (5.76)	0.25 ns	0.12 ns	0.40 ns
RKC	94.93%	137.77%	27.41%	0.56%	4.13 (5.74)	0.85 ns	0.07 ns	0.83 ns
SK/RK	96.39%	146.88%	31.92%	3.29%	4.11 (5.73)	0.39 ns	0.12 ns	0.71 ns
SK/N	97.92%	172.77%	42.12%	4.64%	4.11 (5.73)	0.19 ns	0.09 ns	0.64 ns
RK/N	100.00%	144.69%	30.89%	0.77%	4.10 (5.72)	0.86 ns	0.05^*^	0.86 ns

^ns^ *P*>0.05, ^*^ *P*<0.05; ^a^Values in parentheses are the Shannon-Weiner diversity index of the entire collection.

CR %, VR %, VD %, and MD % are coincidence rate of range, variable rate, variance difference percentage, and mean difference percentage for 14 traits between the entire and mini-core collections, respectively.

RGR, salt-tolerance index of shoot growth rate; RNIL, salt-tolerance index of leaves increased number; RLER, salt-tolerance index of leaf expansion rate; Sen, leaf senescence scale; SWC, shoot water content; RWC, root water content; SNC, shoot Na^+^ concentration; RNC, root Na^+^ concentration; SN/RN, the ratio of shoot Na^+^ concentration to root Na^+^ concentration; SKC, shoot K^+^ concentration; RKC: root K^+^ concentration; SK/RK, the ratio of shoot K^+^ concentration to root K^+^ concentration; SK/N, the ratio of shoot K^+^ concentration to shoot Na^+^ concentration; RK/N, the ratio of root K^+^ concentration to root Na^+^ concentration.

In the mini-core collection of *M. sacchariflorus* and *M. lutarioriparius*, significant positive correlations were observed between SWC and RWC (r=0.76, r=0.73 in the entire set), as well as between Sen and SNC (r=0.70, r=0.66 in the entire set). Additionally, significant positive correlations were found between SKC and SNC, whereas the correlation between RKC and SNC was insignificant. Sen exhibited a significantly negative correlation with RNIL (r=-0.52, r=-0.52 in the entire set) and RLER (r=-0.48, r=-0.45 in the entire set). The correlation trends between traits in the core set resembled those observed in the entire set (**Figure 5**). Furthermore, the PCA conducted on the core set resulted in the extraction of five PCs, which explained 82.90% of the total variation in the mini core set. PC1, PC2, PC3, PC4, and PC5 accounted for 26.79%, 23.91%, 13.21%, 10.20%, and 8.78% of total variation, respectively (**Table 3**). PC1 displayed significant correlations with RK/N, RKC, SWC, RWC (positive loading), SK/RK and RNC (negative loading), whereas PC2 primarily included Sen, SN/RN and SNC with a positive loading, and SK/N, RNIL and RLER with a negative loading (**Figure 6**). These values were comparable to those obtained for the entire set.

## 4 Discussion

### 4.1 Screening system

The susceptibility of plants to environmental conditions during the seedling stage highlights the importance of evaluating salt tolerance based on plant responses at the seedling stage as a crucial screening criterion. However, the genetic diversity of salt tolerance in these species remains largely unknown due to the self- incompatibility of *Miscanthus* (Jiang et al., 2017) and the high heterozygosity of their genetic background. This complicated the use of plants at the seedling stage for stress experiments in subsequent genomic studies, as it is costly to obtain plants through asexual propagation (O’Loughlin et al., 2018). Previous studies by Chen et al. (2017) explored salt tolerance of 70 *Miscanthus* genotypes at the seedling stage (including 57 *M. sinensis*, 5 *M. sacchariflorus*, and 8 hybrids) using an indoor hydroponic system. The present study represents the first attempt to assess the genetic diversity of salt tolerance in *M. sacchariflorus* and *M. lutarioriparius* at the seedling stage using a large sample size of 318 genotypes. Our methodology involved planting rhizomes, each with 1-2 buds of a specific genotype, in plug trays prior to stress application. Hydroponics offer a favorable alternative for maintaining consistent screening conditions within a large population by providing uniform root environments and accommodating a high capacity of different genotypes (Tavakkoli et al., 2012; Nguyen et al., 2013). Single plants of similar heights from each genotype were selected and transferred to a homemade hydroponic setup after thorough root cleaning to ensure the uniformity of the tested plant materials. It is reported that employing such a similar system has allowed for successful assessment of salt tolerance and cadmium accumulation in different rapeseed germplasms, with related QTLs identified (Wan et al., 2017; Chen et al., 2018). Moreover, to account for the inherent differences among genotypes, our methodology also adopted the usage of relative values of growth traits, a widely-used approach (Wu et al., 2019; Luo et al., 2021), to evaluate the performance of different *M. sacchariflorus* and *M. lutarioriparius* genotypes under salt stress.

### 4.2 Mechanisms of salt tolerance

Studies have demonstrated that high concentrations of soil salts damage plants by causing ion (mainly Na^+^) toxicity and osmotic stress (Munns and Tester, 2008). Therefore, osmotic tolerance and ion exclusion need to be considered together when improving salt tolerance in plants (Genc et al., 2010). Low Na^+^ concentrations in shoots have been successfully used as selection criteria for breeding salt-tolerant cultivars of durum wheat (Munns et al., 2012), barley (Nguyen et al., 2013), and rice (Lin et al., 2004). Our results indicated that salt-tolerant genotypes exhibited significantly lower SNC, which is consistent with previous studies on salt tolerance in *M. sinensis* (Sun et al., 2014; Chen et al., 2017; Sun et al., 2021), *M.* × *giganteus* (Plazek et al., 2014; Stavridou et al., 2020), and polyploid *M. lutarioriparius* (Duan et al., 2018). These findings suggested that the mechanisms of Na^+^ exclusion utilized to improve salt tolerance in cereals were also employed by *M. sacchariflorus* and *M. lutarioriparius*. Notably, our study revealed that two genotypes (M283 and M349) exhibited lower Sen and higher RGR, RNIL, RLER, and SWC despite having high shoot Na^+^ concentrations. This observation may indicate the existence of a tissue tolerance mechanism, with Na^+^ being compartmentalized within vacuoles to prevent toxic concentrations in the cytoplasm (Munns and James, 2003).

However, under salt stress, high Na^+^ concentrations interfere with K^+^ uptake and function (Shabala and Cuin, 2008). Maintaining a high K^+^ concentration is another important mechanism in response to relatively high Na^+^ levels under salt stress (Munns and James, 2003; Krishnamurthy et al., 2007). In the present study, the evaluation of ten genotypes based on different salt concentrations showed that most genotypes had higher SKC and RKC under salt stress than under the control. Moreover, our evaluation of 318 genotypes under 150 mM NaCl revealed that genotypes with high Na^+^ concentrations displayed higher K^+^ concentrations than those with low Na^+^ concentrations. These results can be attributed to the adaptive response of *M. sacchariflorus* and *M. lutarioriparius* to excessive Na^+^ levels in their surrounding environments, leading to increased uptake and transport of K^+^ possibly through the enhanced activity of K^+^ channels in the cell membrane and transporter proteins. This response allows the maintenance of stable levels of intracellular K^+^ and Na^+^, ultimately enabling normal growth and metabolic functions. Previous studies have reported a higher K^+^ content under salt stress than under the control in *M.* × *giganteus* (Plazek et al., 2014; Stavridou et al., 2020) and polyploid *M. lutarioriparius* (Duan et al., 2018). Plazek et al. (2014) concluded that a high accumulation of K^+^ in leaves reduces the Na^+^ effect and determines the salinity tolerance of *Miscanthus*. Our study showed a strong positive correlation between the ability of plants to retain K^+^ after exposure to NaCl and salinity tolerance, which is consistent with previous reports in a wide range of plants, including wheat (Cuin et al., 2008), Hami melon (Xiong et al., 2018), *Nitraria sibirica* (Tang et al., 2018), pumpkin (Huang et al., 2019) and *Puccinellia nuttalliana* (Vaziriyeganeh et al., 2022).

### 4.3 Evaluation of salt tolerance of *M. sacchariflorus* and *M. lutarioriparius* by multivariate analysis

The stress resistance capacity of plants results from their responses to adverse environmental conditions and long-term evolution. As it is a quantitative trait controlled by multiple genes, using a single index to evaluate the stress resistance of plants is unreliable. For a more comprehensive assessment of plant stress resistance, employing multidimensional indices is considered more scientifically sound (Wu et al., 2019; Weng et al., 2021). Membership function analysis has gained a wider use in recent years for evaluating plant stress tolerance in that it addresses the limitations of selecting plant varieties based on a single index (Wassie et al., 2019; Weng et al., 2021; Wang et al., 2023). In the present study, through membership function analysis, 14 individual indicators were integrated into a comprehensive evaluation index of salt tolerance (D value) for different genotypes of *M. sacchariflorus* and *M. lutarioriparius* at the seedling stage, with a higher D value indicating stronger salt tolerance ability. Cluster analysis based on the D value would lead to a more objective selection of three HST, 50 ST, 127 MST, 117 SS and 21 HSS genotypes. This outcome diverges from what would be obtained if any single indicator were used, suggesting that salt tolerance evaluation of *M. sacchariflorus* and *M. lutarioriparius* could not rely on a single indicator. All genotypes of *M. sacchariflorus* and *M. lutarioriparius* were evaluated for salt tolerance for the first time. Further studies are needed to compare differences in physiological indicators among genotypes of *M. sacchariflorus* and *M. lutarioriparius* with different salt tolerance levels and their performance in saline soils. Our findings may provide materials with extreme salt tolerance for further studies on the mechanisms of *M. sacchariflorus* and *M. lutarioriparius* in response to salt stress using comparative transcriptome and selective sweep analysis.

Determining the salt tolerance of one or some *M. sacchariflorus* and *M. lutarioriparius* genotypes is challenging without a large number of other genotypes for comparison. Assessing 14 traits and calculating the D value for salt tolerance evaluation is laborious and complicated. To evaluate the salt tolerance of *M. sacchariflorus* and *M. lutarioriparius* in a more convenient and reliable manner, a mathematical evaluation model for the salt tolerance was established using multiple regression analysis. The larger the Y value, the higher the salt tolerance. As a result, it is only necessary to measure seven traits: SNC, RNIL, RWC, RGR, RKC, Sen, and RNC to calculate the Y value for estimating salt tolerance in any genotypes of *M. sacchariflorus* and *M. lutarioriparius*. To our best knowledge, this is the first time a mathematical evaluation model is established to predict the salt tolerance of *M. sacchariflorus* and *M. lutarioriparius* during the seedling stage.

### 4.4 Development of a mini-core collection

This study represents a pioneering effort to establish a core collection for the salt tolerance of *M. sacchariflorus* and *M. lutarioriparius* at the seedling stage. The salt-tolerant traits used were able to generate a mini-core collection that captured the full range of trait variability that existed in the entire collection. This provides the foundation for studying the salt tolerance mechanism through marker-trait associations, which is essential for the conservation of genetic resources and the breeding of salt-tolerant *M. sacchariflorus* and *M. lutarioriparius* lines.

The mini-core collection constituted approximately 20% of the evaluated entire collection, falling within the recommended size range of 10% to 30% for a well-representative core collection (Tchokponhoue et al., 2020; Uba et al., 2023). Additionally, a mini-core collection with a CR % exceeding 80% is deemed suitable for breeding (Hu et al., 2000). High values of VR % and VD % indicate the successful preservation of diversity from the entire set in the core set (Mahmoodi et al., 2019). The MD % for all studied traits was below 20%, indicating that the mini-core collection effectively represented the entire collection. Notably, no significant differences were observed between the core and entire collections in terms of means, variances (for all traits except for RGR, SN/RN, and RK/N), and medians, further validating the representativeness of the mini-core collection.

When establishing a core set, it is crucial to preserve the phenotypic associations within the entire collection to maintain co-adapted genetic complexes and enable efficient germplasm utilization (Tripathi et al., 2022). The results of the Pearson’s correlation analysis for the 14 traits showed that the correlation coefficients between all combinations of traits remained similar in both the core and entire sets. This preservation of trait associations in the core set aligns with findings from previous studies on core collection development in various crops, such as turnips (Li et al., 2021), wheat (Phogat et al., 2021), mustards (Nanjundan et al., 2022), and lentils (Tripathi et al., 2022). Furthermore, five PCs were identified in the core and entire sets, which collectively explained 82.90% and 81.42% of the total variation, respectively. This indicates that the mini-core collection established in this study effectively represents the overall variability of the entire collection. The use of PCA to assess the spatial distribution of entries and to explain the variance serves as an exploratory criterion for evaluating the core set, as has been reported in previous studies (Li et al., 2021; Tripathi et al., 2022).

## 5 Conclusion

This study established that the optimal NaCl concentration for evaluating the salt tolerance of *M. sacchariflorus* and *M. lutarioriparius* is 150 mM. Significant genotype-dependent differences in salt tolerance were observed at the seedling stage in both species. *M. sacchariflorus* and *M. lutarioriparius* adapt to salt stress by regulating ion homeostasis primarily through enhanced K^+^ uptake, shoot Na^+^ exclusion, and Na^+^ sequestration in shoot vacuoles. A total of 318 genotypes were evaluated, resulting in the identification of three HST, 50 ST, 127 MST, 117 SS, and 21 HSS genotypes of *M. sacchariflorus* and *M. lutarioriparius* at the seedling stage. A mathematical evaluation model was proposed to assess salt tolerance by using fourteen traits in the 318 *M. sacchariflorus* and *M. lutarioriparius* genotypes, leading to the development of a representative mini core set of 64 genotypes. These findings significantly contribute to the evaluation and breeding of salt-tolerant *M. sacchariflorus* and *M. lutarioriparius*. Moreover, these also provide valuable resources for an in-depth understanding of the adaptive mechanisms and molecular regulatory networks of *M. sacchariflorus* and *M. lutarioriparius* in response to salt stress, thereby offering scientific support for future efforts to enhance salt tolerance and improve stress resilience in plants. This study is of great implication for the utilization and improvement of marginal lands.

## Data availability statement

The original contributions presented in the study are included in the article/**Supplementary Material**. Further inquiries can be directed to the corresponding authors.

## Author contributions

YT: Conceptualization, Data curation, Formal Analysis, Investigation, Methodology, Software, Visualization, Writing – original draft, Writing – review & editing. SL: Investigation, Software, Writing – original draft. DZ: Writing – review & editing. ZZ: Writing – review & editing. SY: Resources, Writing – original draft. XZ: Writing – review & editing. SX: Resources, Writing – original draft. XK: Writing – review & editing. ML: Methodology, Writing – original draft. XH: Investigation, Writing – original draft. ZY: Conceptualization, Funding acquisition, Resources, Writing – review & editing. LX: Project administration, Supervision, Writing – review & editing, Writing – original draft.
