# Supplementary material for: Salt tolerance evaluation and mini-core collection development in Miscanthus sacchariflorus and M. lutarioriparius
Source: Front Plant Sci. 2024 Mar 5;15:1364826. doi: 10.3389/fpls.2024.1364826 (PMC10948507; doi:10.3389/fpls.2024.1364826)
Supplement: Supplementary file 1 [file DataSheet_1.pdf]

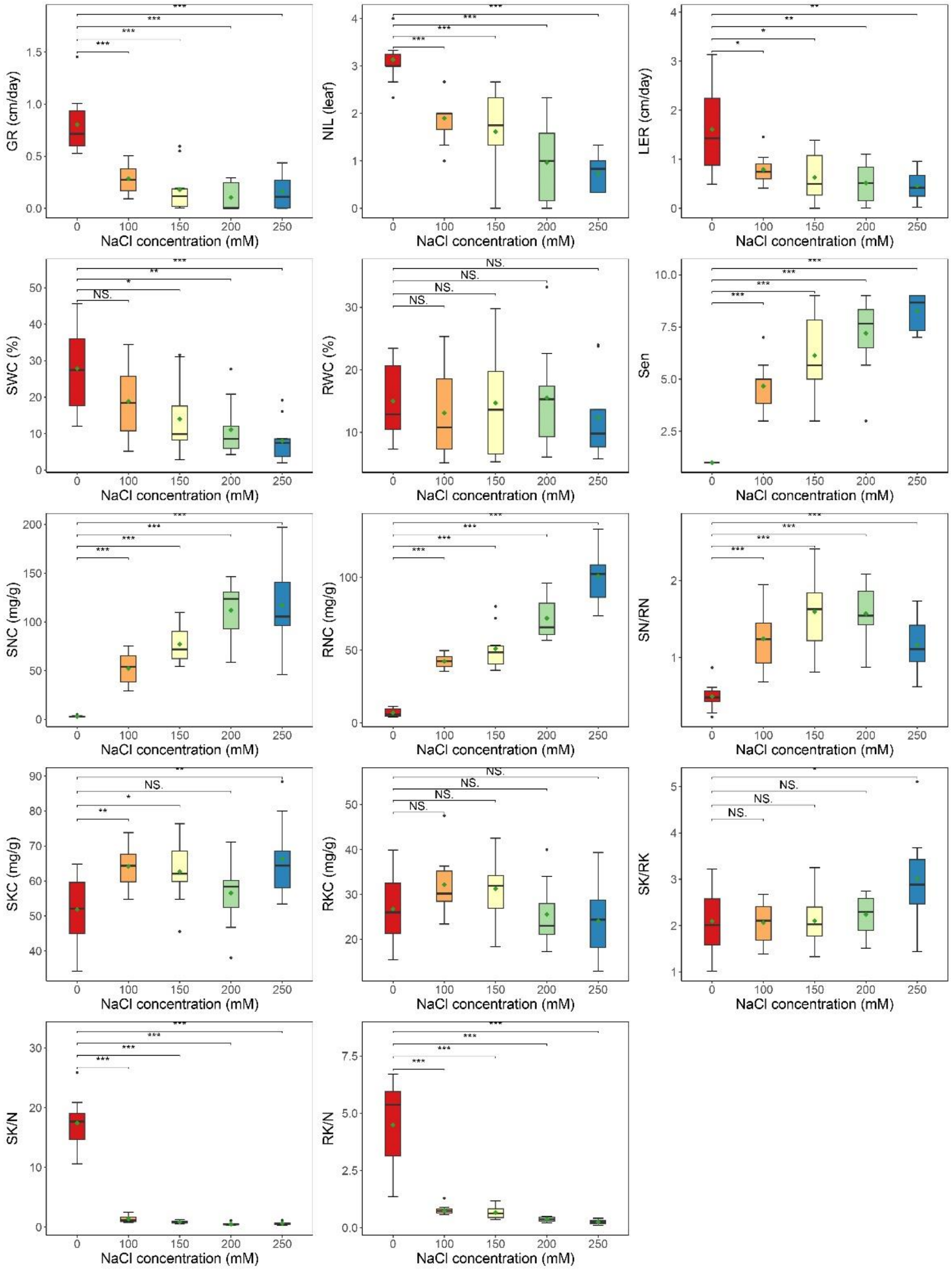

Supplementary Figure S1. Effect of different concentrations of NaCl treatments on the traits of ten *M. sacchariflorus* and *M. lutarioriparius* seedlings. Significance of the t-test: $^{NS.}$ p>0.05, $^{*}p$<0.05, $^{**}p$<0.01, $^{***}p$<0.001. GR: Shoot growth rate, NIL: leaves increased number, LER: leaf expansion rate, Sen: leaf senescence scale, SWC: shoot water content, RWC: root water content, SNC: shoot Na^+ concentration, RNC: root Na^+ concentration, SN/RN: the ratio of shoot Na^+ concentration to root Na^+ concentration, SKC: shoot K^+ concentration, RKC: root K^+ concentration, SK/RK: the ratio of shoot K^+ concentration to root K^+ concentration, SK/N: the ratio of shoot K^+ concentration to shoot Na^+ concentration, RK/N: the ratio of root K^+ concentration to root Na^+ concentration.

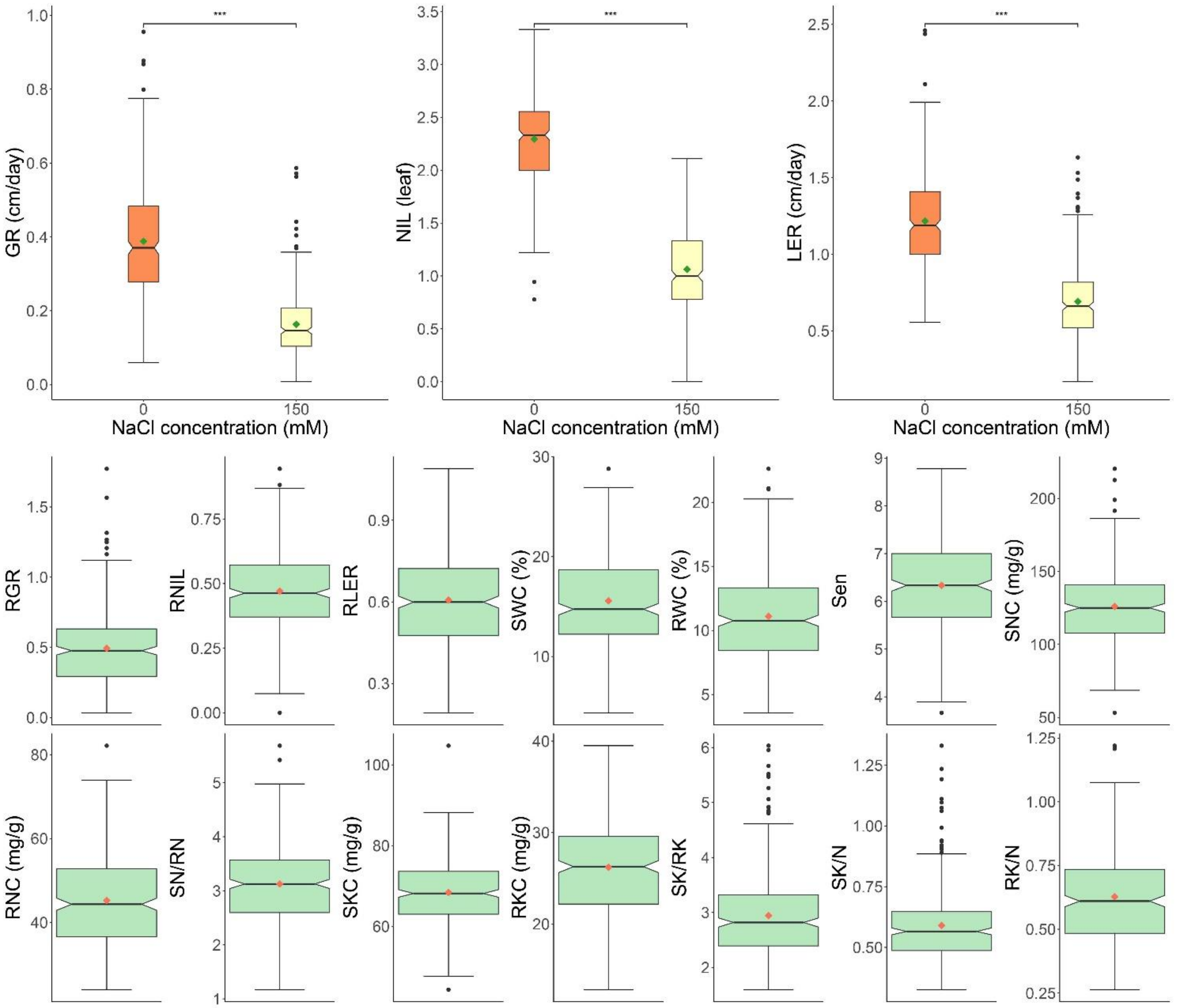

Supplementary Figure S2. Characterization of 14 indicators under 150 mM NaCl treatment, and three traits (GR, NIL, LER) under 0 and 150 mM NaCl treatment in 318 *M. sacchariflorus* and *M. lutarioriparius* seedlings. Significance of the t-test: $^{***}p<0.001$. GR: Shoot growth rate, NIL: leaves increased number, LER: leaf expansion rate, RGR: salt-tolerance index of shoot growth rate, RNIL: salt-tolerance index of leaves increased number, RLER: salt-tolerance index of leaf expansion rate, Sen: leaf senescence scale, SWC: shoot water content, RWC: root water content, SNC: shoot Na^+ concentration, RNC: root Na^+ concentration, SN/RN: the ratio of shoot Na^+ concentration to root Na^+ concentration, SKC: shoot K^+ concentration, RKC: root K^+ concentration, SK/RK: the ratio of shoot K^+ concentration to root K^+ concentration, SK/N: the ratio of shoot K^+ concentration to shoot Na^+ concentration, RK/N: the ratio of root K^+ concentration to root Na^+ concentration.

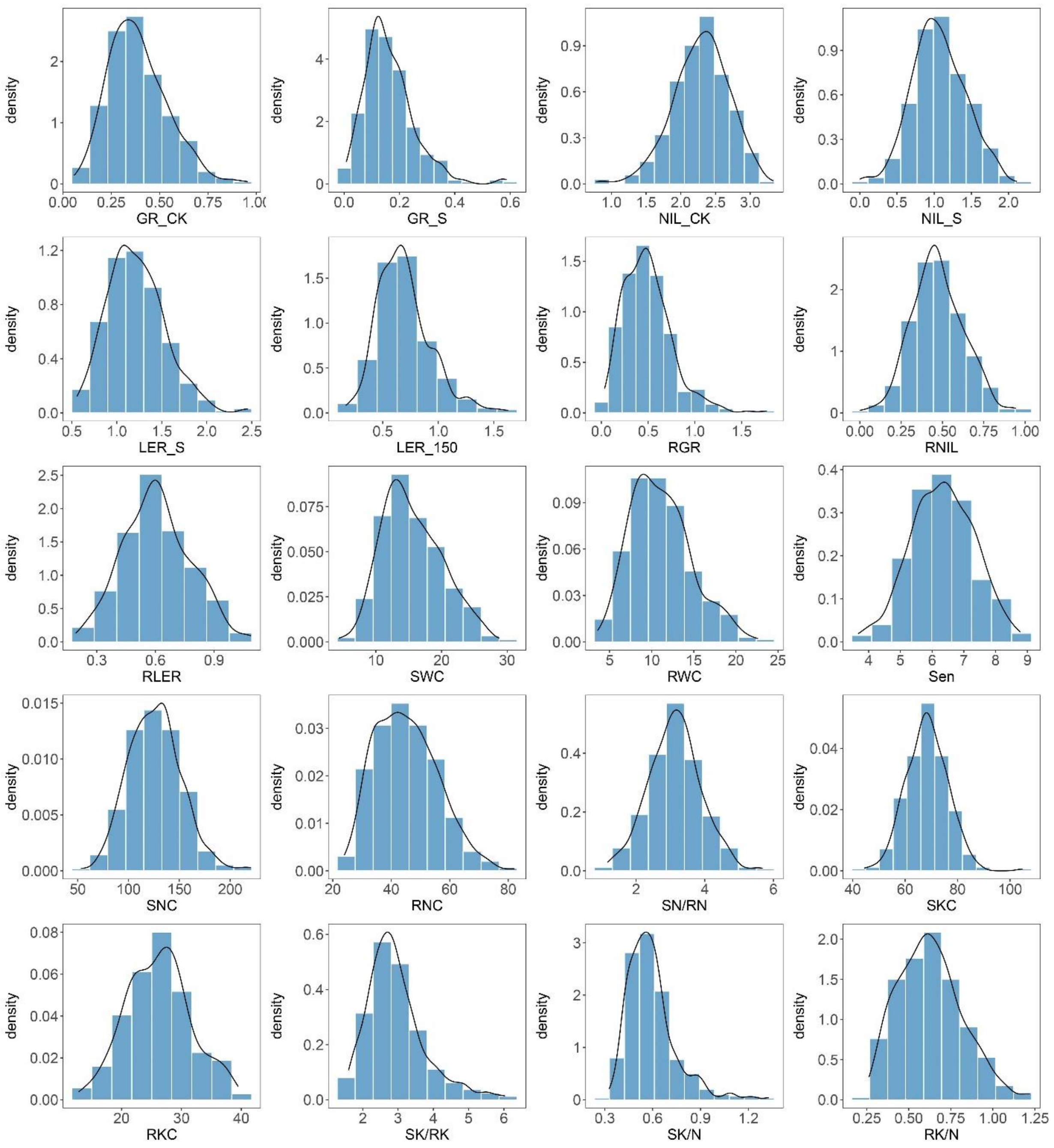

Supplementary Figure S3. The frequency distribution of all traits in 318 *M. sacchariflorus* and *M. lutarioripa* genotypes. GR_CK: Shoot growth rate under control, GR_S: Shoot growth rate under 150 mM NaCl, RGR: salt-tolerance index of shoot growth rate, NIL_CK: leaves increased number under control, NIL_S: leaves increased number under 150 mM NaCl, RNIL: salt-tolerance index of leaves increased number, LER_CK: leaf expansion rate under control, LER_S: leaf expansion rate under 150 mM NaCl, RLER: salt-tolerance index of leaf expansion rate, Sen: leaf senescence scale, SWC: shoot water content, RWC: root water content, SNC: shoot Na^+ concentration, RNC: root Na^+ concentration, SN/RN: the ratio of shoot Na^+ concentration to root Na^+ concentration, SKC: shoot K^+ concentration, RKC: root K^+ concentration, SK/RK: the ratio of shoot K^+ concentration to root K^+ concentration, SK/N: the ratio of shoot K^+ concentration to shoot Na^+ concentration, RK/N: the ratio of root K^+ concentration to root Na^+ concentration.
