# Supplementary material for: Salt tolerance evaluation and mini-core collection development in Miscanthus sacchariflorus and M. lutarioriparius
Source: Front Plant Sci. 2024 Mar 5;15:1364826. doi: 10.3389/fpls.2024.1364826 (PMC10948507; doi:10.3389/fpls.2024.1364826)
Supplement: Supplementary file 2 [file DataSheet_2.pdf]

Table S1. Information on collection sites for 318 *M. sacchariflorus* and *M. lutarioriparius* accessions.

Genotype	Species name	Province	Country	Longitude/E°	Latitude/N°	Altitude/m
M1	*M. lutarioriparius*	Hunan	Changsha	113.03	28.18	80.00
M3	*M. lutarioriparius*	Hunan	Changsha	113.03	28.18	80.00
M4	*M. sacchariflorus*	Zhejiang	Shaoxing	121.06	29.49	199.38
M5	*M. lutarioriparius*	Jiangxi	Hukou	116.21	29.74	9.00
M7	*M. lutarioriparius*	Hunan	Changsha	112.89	28.23	54.00
M10	*M. lutarioriparius*	Hunan	Yueyang	112.56	29.53	34.00
M11	*M. lutarioriparius*	Hunan	Yueyang	112.84	29.39	6.00
M13	*M. lutarioriparius*	Hunan	Yuanjiang	112.36	28.85	113.00
M14*	*M. lutarioriparius*	Hunan	Yuanjiang	112.36	28.85	113.00
M17	*M. sacchariflorus*	Shaanxi	Chenggu	107.19	33.27	704.00
M18	*M. sacchariflorus*	Henan	Zhoukou	115.42	34.15	31.07
M19	*M. lutarioriparius*	Hunan	Anxiang	112.14	29.43	15.67
M20*	*M. lutarioriparius*	Hunan	Anxiang	112.15	29.16	17.82
M21	*M. lutarioriparius*	Hunan	Xiangyin	112.89	28.80	20.66
M22	*M. lutarioriparius*	Jiangsu	Wuxi	119.90	31.35	11.33
M24	*M. sacchariflorus*	Zhejiang	Ningbo	121.19	29.60	82.36
M25	*M. sacchariflorus*	Anhui	Anqing	117.01	31.13	42.86
M27	*M. lutarioriparius*	Hubei	Huanggang	115.63	29.86	4.63
M28	*M. sacchariflorus*	Jiangsu	Huaian	118.90	33.71	10.89
M29*	*M. sacchariflorus*	Jiangsu	Wuxi	119.70	31.53	6.17
M30	*M. sacchariflorus*	Anhui	Bengbu	117.21	32.80	19.48
M31	*M. sacchariflorus*	Anhui	Hefei	117.10	32.02	56.33
M32*	*M. sacchariflorus*	Zhejiang	Huzhou	120.02	30.90	40.62
M33	*M. lutarioriparius*	Hunan	Yiyang	112.73	29.16	11.55
M34	*M. lutarioriparius*	Hunan	Yiyang	112.20	29.15	19.92
M35*	*M. lutarioriparius*	Hunan	Changde	112.08	29.28	16.34
M37*	*M. sacchariflorus*	Shandong	Jiaxiang	116.43	35.25	27.94
M38	*M. lutarioriparius*	Hunan	Yiyang	112.23	29.14	12.01
M39	*M. sacchariflorus*	Hubei	Tianmen	113.49	30.44	24.28
M40	*M. lutarioriparius*	Hunan	Yiyang	112.46	28.92	14.63
M42	*M. sacchariflorus*	Henan	Xinyang	114.34	33.04	29.83
M43	*M. sacchariflorus*	Shandong	Laiwu	116.32	36.30	28.20
M45*	*M. lutarioriparius*	Hunan	Yiyang	112.30	29.07	12.44
M46	*M. lutarioriparius*	Hunan	Yiyang	112.19	29.17	19.29
M47	*M. lutarioriparius*	Jiangsu	Huaian	119.15	33.64	16.38
M50	*M. lutarioriparius*	Hunan	Yiyang	112.34	28.97	9.38
M51	*M. lutarioriparius*	Hunan	Yiyang	112.28	29.07	12.09
M55	*M. lutarioriparius*	Hunan	Yueyang	112.95	29.00	13.08
M57	*M. lutarioriparius*	Hubei	Xianning	113.76	29.92	15.68
M59	*M. sacchariflorus*	Jiangsu	Suqian	118.67	33.76	14.44
M60	*M. lutarioriparius*	Hunan	Yueyang	112.79	28.63	15.66
M63	*M. sacchariflorus*	Jiangsu	Yancheng	119.82	33.93	4.92
M66*	*M. lutarioriparius*	Hunan	Yiyang	112.27	29.09	17.93
M67	*M. sacchariflorus*	Zhejiang	Jiaxing	120.91	30.53	16.34
M69*	*M. lutarioriparius*	Hunan	Yueyang	112.76	29.22	17.27
M73	*M. sacchariflorus*	Liaoning	Dalian	121.72	39.10	970.00
M74*	*M. sacchariflorus*	Heilongjiang	Jiamusi	130.32	46.80	117.00
M76	*M. sacchariflorus*	Liaoning	Dalian	121.61	38.91	978.00
M77	*M. sacchariflorus*	Jilin	Jiaohe	127.21	43.71	410.00
M79	*M. lutarioriparius*	Hunan	Yueyang	113.43	29.06	105.00
M85	*M. lutarioriparius*	Hunan	Changde	112.18	28.83	16.09

M86	*M. lutarioriparius*	Hunan	Yueyang	113.14	29.35	98.00
M87	*M. lutarioriparius*	Hubei	Jianli	112.89	29.82	107.00
M88*	*M. sacchariflorus*	Ningxia	Yongning	106.27	38.33	1080.00
M89	*M. sacchariflorus*	Shandong	Rizhao	119.56	35.37	0.00
M90	*M. sacchariflorus*	Shaanxi	Zhouzhi	108.17	33.97	700.00
M92	*M. sacchariflorus*	Jiangsu	Dafeng	120.81	32.99	2.00
M96	*M. sacchariflorus*	Hubei	Anlu	113.71	31.35	0.00
M99	*M. lutarioriparius*	Henan	Nanyang	112.42	32.94	0.00
M100*	*M. sacchariflorus*	Hunan	Yueyang	112.74	29.32	6.94
M101	*M. lutarioriparius*	Hunan	Yueyang	112.88	28.89	10.57
M102	*M. sacchariflorus*	Henan	Nanyang	111.48	33.12	0.00
M103	*M. lutarioriparius*	Henan	Xinyang	114.45	31.93	0.00
M104	*M. sacchariflorus*	Jilin	Jiaohe	127.18	43.68	390.00
M106	*M. lutarioriparius*	Anhui	Mingguang	117.95	32.75	0.00
M107	*M. lutarioriparius*	Anhui	Huoqiu	116.31	32.43	0.00
M108*	*M. lutarioriparius*	Anhui	Chuzhou	117.51	32.55	0.00
M109	*M. lutarioriparius*	Hubei	Gucheng	111.62	32.29	0.00
M110	*M. lutarioriparius*	Hubei	Yicheng	111.56	30.26	0.00
M112	*M. sacchariflorus*	Chongqing	Yongchuan	105.89	29.05	0.00
M113	*M. sacchariflorus*	Hubei	Gucheng	111.67	32.25	0.00
M115	*M. lutarioriparius*	Anhui	Shucheng	117.00	31.54	0.00
M116*	*M. lutarioriparius*	Jiangsu	Gaoyou	119.76	32.83	0.00
M117	*M. lutarioriparius*	Hubei	Wuhan	113.99	30.76	0.00
M118	*M. lutarioriparius*	Hubei	Xiangyang	112.01	31.78	0.00
M119	*M. lutarioriparius*	Hubei	Gucheng	111.62	32.29	0.00
M120	*M. lutarioriparius*	Henan	Dengzhou	111.95	32.59	0.00
M121	*M. lutarioriparius*	Hunan	Changde	112.15	28.77	13.29
M122*	*M. lutarioriparius*	Henan	Dengzhou	111.95	32.59	0.00
M123*	*M. lutarioriparius*	Jiangsu	Gaoyou	119.30	32.69	0.00
M124	*M. lutarioriparius*	Hunan	Changde	112.15	28.80	9.66
M125	*M. lutarioriparius*	Anhui	Quanjiao	118.04	31.91	0.00
M126	*M. lutarioriparius*	Hunan	Changde	112.02	29.41	13.72
M127	*M. lutarioriparius*	Hunan	Changde	112.07	29.36	10.80
M128	*M. sacchariflorus*	Shandong	Laiwu	117.48	36.22	159.70
M129	*M. lutarioriparius*	Hunan	Changde	112.10	28.70	61.77
M130	*M. lutarioriparius*	Hunan	Changde	112.05	29.38	10.73
M131	*M. lutarioriparius*	Hubei	Xianning	113.91	29.99	17.89
M132	*M. lutarioriparius*	Hunan	Yueyang	113.06	29.45	11.39
M133	*M. lutarioriparius*	Hubei	Jingzhou	113.35	30.12	10.07
M134	*M. lutarioriparius*	Jiangsu	Yancheng	119.95	33.56	9.43
M135*	*M. sacchariflorus*	Hubei	Xiaogan	113.62	30.59	16.01
M136	*M. sacchariflorus*	Shandong	Zibo	118.06	36.19	313.96
M137	*M. lutarioriparius*	Hunan	Yueyang	112.78	29.26	17.14
M138	*M. lutarioriparius*	Hunan	Yueyang	113.11	29.44	18.15
M139*	*M. sacchariflorus*	Shanxi	Xixian	110.95	36.74	980.00
M140	*M. lutarioriparius*	Hunan	Changde	112.07	29.31	8.30
M141	*M. lutarioriparius*	Hunan	Yuanjiang	112.36	28.85	35.00
M142	*M. lutarioriparius*	Hunan	Yuanjiang	112.36	28.85	35.00
M143	*M. sacchariflorus*	Shaanxi	Qianyang	107.06	34.71	734.00
M144	*M. lutarioriparius*	Hunan	Changsha	112.87	28.36	18.48
M145	*M. sacchariflorus*	Henan	Linying	113.91	33.89	82.00
M146	*M. sacchariflorus*	Hebei	Lincheng	114.27	37.55	208.00
M147	*M. lutarioriparius*	Henan	Linzhou	113.75	36.26	554.00
M148	*M. sacchariflorus*	Hebei	Kuancheng	118.40	40.59	276.00

M149*	*M. sacchariflorus*	Hebei	Guantao	115.25	36.54	26.00
M150	*M. sacchariflorus*	Hebei	Tangshan	118.15	39.76	37.00
M151	*M. sacchariflorus*	Hebei	Chengde	117.84	40.90	351.00
M152	*M. sacchariflorus*	Hubei	Dawu	114.27	31.61	106.00
M153*	*M. sacchariflorus*	Henan	Weihui	114.07	35.45	76.00
M154	*M. sacchariflorus*	Henan	Huangchuan	115.04	32.23	49.00
M155	*M. sacchariflorus*	Hebei	Fuping	114.29	38.71	391.00
M156	*M. sacchariflorus*	Henan	Xinxian	114.85	31.60	108.00
M157	*M. sacchariflorus*	Hubei	Hongan	114.67	31.46	84.00
M158	*M. sacchariflorus*	Shaanxi	Fuxian	109.45	35.99	1246.00
M159*	*M. lutarioriparius*	Jiangxi	Hukou	116.38	29.80	18.00
M160	*M. lutarioriparius*	Hubei	Huangmei	115.91	30.05	16.00
M161	*M. sacchariflorus*	Hebei	Jingxing	114.17	37.76	657.00
M162	*M. sacchariflorus*	Shaanxi	Shangluo	110.64	33.59	424.42
M164	*M. lutarioriparius*	Shandong	Weishan	117.08	34.82	26.00
M165*	*M. sacchariflorus*	Anhui	Jinzhai	115.80	31.20	480.00
M166*	*M. lutarioriparius*	Jiangxi	Hukou	116.21	29.74	9.00
M167*	*M. sacchariflorus*	Jiangsu	Xuzhou	117.32	34.53	36.00
M168*	*M. sacchariflorus*	Anhui	Jinzhai	115.96	31.73	88.00
M169	*M. sacchariflorus*	Anhui	Anshan	118.53	31.67	14.00
M170	*M. sacchariflorus*	Shandong	Jinan	117.30	36.63	861.00
M171	*M. lutarioriparius*	Anhui	Huangshan	118.24	30.13	314.00
M172	*M. lutarioriparius*	Anhui	Xuancheng	118.72	30.92	93.00
M173	*M. lutarioriparius*	Anhui	Jingde	118.46	30.35	148.00
M174	*M. lutarioriparius*	Anhui	Jingxian	118.45	30.54	113.00
M175	*M. lutarioriparius*	Anhui	Jingxian	118.45	30.54	113.00
M176	*M. sacchariflorus*	Shandong	Dongying	118.98	37.76	0.00
M177*	*M. lutarioriparius*	Anhui	Dongzhi	116.96	30.22	30.00
M179	*M. sacchariflorus*	Henan	Xinxiang	113.70	35.00	66.08
M181	*M. lutarioriparius*	Hunan	Yueyang	112.81	29.43	8.45
M183	*M. sacchariflorus*	Shaanxi	Shangluo	109.98	33.84	649.02
M185	*M. lutarioriparius*	Hunan	Yiyang	112.28	29.05	15.65
M187	*M. lutarioriparius*	Hubei	Huangshi	114.66	30.01	39.06
M189*	*M. lutarioriparius*	Hubei	Xiaogan	113.83	30.68	14.49
M190	*M. lutarioriparius*	Hunan	Yiyang	112.33	28.85	12.72
M191	*M. sacchariflorus*	Shandong	Linyi	117.80	35.38	128.41
M192*	*M. sacchariflorus*	Shandong	Jining	116.91	35.52	44.78
M193	*M. lutarioriparius*	Hunan	Yueyang	113.11	29.44	18.15
M195	*M. sacchariflorus*	Anhui	Hefei	117.10	32.27	41.88
M196	*M. lutarioriparius*	Anhui	Anqing	116.16	29.86	5.02
M198	*M. lutarioriparius*	Hunan	Changde	112.11	28.73	29.48
M199	*M. lutarioriparius*	Anhui	Anqing	116.72	30.27	15.89
M201	*M. sacchariflorus*	Shandong	Rizhao	118.81	35.62	110.60
M203*	*M. sacchariflorus*	Henan	Luoyang	112.76	34.66	99.08
M204	*M. lutarioriparius*	Hunan	Changde	112.04	29.52	14.36
M205*	*M. lutarioriparius*	Hunan	Changde	112.11	28.88	19.07
M206	*M. lutarioriparius*	Anhui	Huangshan	118.21	30.10	294.00
M207	*M. lutarioriparius*	Hunan	Changde	112.03	28.91	17.44
M209	*M. lutarioriparius*	Henan	Xichuan	111.48	33.11	168.00
M210	*M. lutarioriparius*	Anhui	Anqing	116.34	29.91	7.12
M211	*M. lutarioriparius*	Hunan	Changde	112.09	28.91	14.53
M212	*M. lutarioriparius*	Hunan	Yiyang	112.21	29.20	19.50
M214	*M. lutarioriparius*	Jiangxi	Jinxian	116.29	28.63	20.00
M215	*M. lutarioriparius*	Anhui	Wuhu	118.40	31.38	7.00

M216	*M. lutarioriparius*	Anhui	Wuhu	118.40	31.38	7.00
M217	*M. lutarioriparius*	Hunan	Yueyang	113.02	29.50	11.67
M220	*M. lutarioriparius*	Anhui	Dongzhi	117.06	30.49	20.00
M221	*M. lutarioriparius*	Hunan	Changde	112.19	28.80	11.61
M223	*M. lutarioriparius*	Shandong	Weishan	117.22	34.73	23.00
M224	*M. sacchariflorus*	Henan	Luoyang	112.18	34.72	192.09
M225	*M. lutarioriparius*	Hunan	Changde	112.03	28.91	17.44
M226	*M. lutarioriparius*	Jiangxi	Yugan	116.51	28.74	29.00
M227	*M. lutarioriparius*	Hunan	Yueyang	112.81	28.61	19.74
M228*	*M. lutarioriparius*	Jiangxi	Xiushui	114.23	29.00	140.00
M229	*M. lutarioriparius*	Jiangxi	Xiushui	114.41	29.03	124.00
M230	*M. sacchariflorus*	Shandong	Jining	117.23	35.66	94.35
M232	*M. sacchariflorus*	Jiangxi	Yongxiu	115.81	29.04	44.00
M233*	*M. lutarioriparius*	Jiangsu	Zhenjiang	119.53	32.04	8.36
M234*	*M. lutarioriparius*	Jiangxi	Wuning	115.40	29.21	87.00
M235	*M. sacchariflorus*	Shandong	Heze	115.99	35.08	26.93
M236	*M. lutarioriparius*	Hunan	Changde	112.10	29.64	24.73
M237*	*M. sacchariflorus*	Shaanxi	Shangluo	110.99	33.50	467.34
M238	*M. lutarioriparius*	Hunan	Changde	112.10	29.25	17.68
M239	*M. sacchariflorus*	Shaanxi	Xianyang	109.02	34.68	384.99
M240	*M. lutarioriparius*	Jiangsu	Huaian	118.42	32.99	21.20
M241	*M. lutarioriparius*	Hubei	Huanggang	115.29	30.36	33.41
M242	*M. lutarioriparius*	Hunan	Yueyang	113.08	29.43	16.07
M243*	*M. sacchariflorus*	Zhejiang	Ningbo	121.40	29.73	12.56
M245	*M. lutarioriparius*	Jiangsu	Suzhou	120.51	31.12	0.00
M246	*M. sacchariflorus*	Hubei	Xiaogan	114.04	31.68	85.88
M247*	*M. lutarioriparius*	Hunan	Changde	112.05	29.57	15.23
M248*	*M. sacchariflorus*	Henan	Xinxiang	113.31	34.91	117.35
M250	*M. lutarioriparius*	Hunan	Yueyang	112.77	28.68	15.53
M251*	*M. lutarioriparius*	Zhejiang	Yuyao	120.97	30.15	0.00
M252*	*M. lutarioriparius*	Anhui	Chaohu	117.75	31.80	0.00
M253	*M. sacchariflorus*	Zhejiang	Shaoxing	120.79	30.12	0.00
M254	*M. lutarioriparius*	Hunan	Changde	112.20	28.83	13.68
M255	*M. lutarioriparius*	Zhejiang	Huzhou	120.22	30.90	0.00
M256	*M. lutarioriparius*	Hunan	Changde	112.13	28.88	20.24
M258*	*M. lutarioriparius*	Hunan	Changde	112.07	29.49	14.54
M260*	*M. lutarioriparius*	Anhui	Dongzhi	117.06	30.27	0.00
M261	*M. lutarioriparius*	Hunan	Changsha	112.85	28.37	21.57
M262	*M. lutarioriparius*	Jiangxi	Pengze	116.59	29.93	0.00
M263	*M. lutarioriparius*	Hunan	Changde	112.06	29.69	21.84
M264	*M. lutarioriparius*	Hunan	Yueyang	113.06	29.42	8.07
M265	*M. lutarioriparius*	Hunan	Changde	112.04	29.65	15.45
M266	*M. lutarioriparius*	Hunan	Yueyang	112.84	28.64	13.64
M267	*M. lutarioriparius*	Hunan	Yueyang	112.74	29.32	10.04
M268	*M. lutarioriparius*	Jiangsu	Suqian	118.07	33.47	0.00
M269	*M. lutarioriparius*	Jiangsu	Suqian	118.40	33.57	0.00
M270	*M. lutarioriparius*	Hunan	Yueyang	113.10	29.44	16.80
M271	*M. lutarioriparius*	Jiangsu	Nanjing	118.92	32.42	0.00
M272	*M. lutarioriparius*	Hunan	Yueyang	112.98	29.41	20.07
M274	*M. lutarioriparius*	Hunan	Yueyang	113.02	29.50	11.67
M275	*M. sacchariflorus*	Jiangsu	Yancheng	120.40	33.32	0.00
M276	*M. lutarioriparius*	Hunan	Yiyang	112.33	28.99	12.20
M277	*M. lutarioriparius*	Anhui	Fuyang	116.28	32.68	0.00
M278	*M. lutarioriparius*	Hunan	Yueyang	112.92	28.83	12.19

M280	*M. lutarioriparius*	Hunan	Xiangtan	112.95	27.43	0.00
M281	*M. lutarioriparius*	Hunan	Yiyang	112.52	28.93	15.00
M282	*M. sacchariflorus*	Henan	Xinyang	114.09	31.92	110.47
M283	*M. lutarioriparius*	Hunan	Xiangtan	112.52	27.49	0.00
M284	*M. lutarioriparius*	Hubei	Jingzhou	113.34	29.69	8.39
M285	*M. lutarioriparius*	Hunan	Changde	112.12	29.47	22.05
M286	*M. lutarioriparius*	Hunan	Changde	112.16	28.86	13.15
M287	*M. lutarioriparius*	Hunan	Changde	112.00	28.93	18.56
M288*	*M. lutarioriparius*	Hunan	Yueyang	112.88	28.92	7.73
M289	*M. lutarioriparius*	Hunan	Xiangtan	113.01	27.90	0.00
M290	*M. lutarioriparius*	Hunan	Hengshan	112.88	27.23	0.00
M291	*M. lutarioriparius*	Hunan	Changde	112.05	29.71	18.68
M292	*M. lutarioriparius*	Hunan	Changde	112.04	29.40	7.01
M293	*M. lutarioriparius*	Hunan	Zhuzhou	113.14	27.69	0.00
M294*	*M. lutarioriparius*	Hunan	Zhuzhou	113.17	27.53	0.00
M295	*M. lutarioriparius*	Hunan	Yueyang	113.10	29.46	13.35
M296	*M. sacchariflorus*	Hunan	Hengdong	112.73	27.00	0.00
M297	*M. sacchariflorus*	Henan	Xinyang	114.35	32.68	47.00
M298	*M. lutarioriparius*	Jiangsu	Yangzhou	119.35	32.72	12.23
M299	*M. lutarioriparius*	Hunan	Qiyang	112.13	26.48	0.00
M300*	*M. lutarioriparius*	Jiangxi	Fuzhou	116.48	27.87	0.00
M301	*M. lutarioriparius*	Hunan	Zhuzhou	113.10	27.85	0.00
M303	*M. lutarioriparius*	Hunan	Xiangtan	112.95	27.88	0.00
M304	*M. sacchariflorus*	Chongqing	Yongchuan	105.89	29.05	0.00
M305	*M. lutarioriparius*	Hunan	Xiangtan	112.98	27.80	0.00
M306	*M. sacchariflorus*	Hunan	Changning	112.40	26.47	0.00
M307	*M. sacchariflorus*	Hunan	Changde	111.91	29.57	14.17
M308*	*M. lutarioriparius*	Hunan	Changde	112.23	28.86	13.07
M311*	*M. lutarioriparius*	Hunan	Yueyang	112.84	29.77	3.08
M312	*M. lutarioriparius*	Hunan	Yiyang	112.42	28.81	6.20
M315	*M. lutarioriparius*	Hunan	Yiyang	112.54	28.84	10.22
M317*	*M. lutarioriparius*	Hunan	Yiyang	112.92	29.04	12.14
M318*	*M. lutarioriparius*	Hunan	Changde	112.10	28.92	8.08
M319*	*M. lutarioriparius*	Hunan	Yiyang	112.88	29.02	8.47
M321	*M. lutarioriparius*	Hunan	Yiyang	112.39	28.86	14.55
M322*	*M. lutarioriparius*	Hunan	Changde	112.18	29.58	8.64
M324	*M. sacchariflorus*	Hunan	Yueyang	112.82	28.89	16.20
M325	*M. lutarioriparius*	Hunan	Yiyang	112.64	28.85	4.35
M326	*M. lutarioriparius*	Hunan	Changde	112.02	28.95	6.68
M328*	*M. lutarioriparius*	Hunan	Yuanjiang	112.40	28.84	8.86
M329	*M. lutarioriparius*	Hunan	Yiyang	112.53	28.88	6.90
M330*	*M. lutarioriparius*	Hunan	Yueyang	113.01	29.36	9.09
M331	*M. lutarioriparius*	Hunan	Changde	112.79	29.46	8.41
M332	*M. lutarioriparius*	Hunan	Changde	112.10	28.92	8.08
M333	*M. lutarioriparius*	Hunan	Yueyang	112.95	29.12	9.61
M334	*M. lutarioriparius*	Hunan	Yueyang	112.92	29.53	6.91
M336	*M. lutarioriparius*	Hunan	Changde	112.03	29.56	9.71
M337	*M. lutarioriparius*	Hunan	Yuanjiang	112.47	28.79	10.13
M338*	*M. lutarioriparius*	Hunan	Yiyang	112.73	28.91	18.20
M339	*M. lutarioriparius*	Hunan	Yueyang	112.98	29.48	17.85
M341	*M. lutarioriparius*	Hunan	Yuanjiang	112.54	28.87	9.23
M342	*M. lutarioriparius*	Hunan	Yueyang	113.01	29.37	7.49
M343	*M. lutarioriparius*	Hunan	Yueyang	112.94	29.09	9.67
M344	*M. lutarioriparius*	Hunan	Changde	112.13	28.88	13.47

M345	*M. lutarioriparius*	Hunan	Changde	112.17	28.86	11.46
M346	*M. lutarioriparius*	Hunan	Yiyang	112.66	28.90	13.58
M347	*M. lutarioriparius*	Hunan	Yiyang	112.59	28.94	13.68
M348	*M. lutarioriparius*	Hunan	Yueyang	112.91	29.65	7.96
M349	*M. lutarioriparius*	Hunan	Changde	112.43	29.63	11.97
M351	*M. lutarioriparius*	Jiangxi	Jinxian	116.34	28.65	0.00
M352	*M. lutarioriparius*	Hunan	Yueyang	112.85	28.86	17.65
M353*	*M. lutarioriparius*	Jiangsu	Nanjing	119.08	31.32	0.00
M355	*M. lutarioriparius*	Hunan	Yueyang	112.89	28.83	7.49
M356	*M. lutarioriparius*	Hunan	Yiyang	112.54	28.84	10.22
M357	*M. lutarioriparius*	Hunan	Yuanjiang	112.45	28.89	9.99
M358*	*M. lutarioriparius*	Hunan	Yueyang	112.86	29.00	6.57
M359	*M. lutarioriparius*	Hunan	Yiyang	112.51	28.81	5.53
M360	*M. lutarioriparius*	Hunan	Yiyang	112.51	28.90	7.94
M361	*M. lutarioriparius*	Hunan	Changde	112.11	28.90	12.78
M362*	*M. lutarioriparius*	Hunan	Yueyang	112.94	29.11	14.10
M363	*M. lutarioriparius*	Hunan	Changde	112.17	28.86	11.46
M364	*M. lutarioriparius*	Jiangsu	Suzhou	120.45	31.22	0.00
M367	*M. lutarioriparius*	Hunan	Yiyang	112.39	28.88	11.03
M371	*M. lutarioriparius*	Hunan	Yiyang	112.30	28.84	13.71
M372	*M. lutarioriparius*	Hunan	Yiyang	112.41	28.82	7.68
M373*	*M. sacchariflorus*	Jiangsu	Siyang	118.72	33.94	0.00
M374	*M. lutarioriparius*	Hunan	Yueyang	112.77	28.56	17.65
M375	*M. lutarioriparius*	Hunan	Yueyang	112.89	28.87	12.50
M376	*M. lutarioriparius*	Hunan	Changde	112.21	28.81	23.52
M377	*M. lutarioriparius*	Anhui	Xuancheng	118.94	31.22	0.00
M378	*M. sacchariflorus*	Hunan	Changde	112.07	29.36	8.88
M379*	*M. lutarioriparius*	Jiangsu	Suzhou	120.39	31.26	0.00
M380	*M. lutarioriparius*	Jiangxi	Nanchang	116.10	28.67	0.00
M381	*M. sacchariflorus*	Hubei	Xiantao	113.41	30.32	4.47
M383	*M. sacchariflorus*	Hunan	Yiyang	112.46	28.92	15.26
M384	*M. lutarioriparius*	Hunan	Changde	112.79	29.46	6.24
M385	*M. lutarioriparius*	Hunan	Yiyang	112.69	28.88	6.46
M386	*M. lutarioriparius*	Hunan	Yiyang	112.70	28.97	14.27
M389	*M. lutarioriparius*	Hunan	Yiyang	112.73	28.88	6.44
M392	*M. lutarioriparius*	Hunan	Yiyang	112.38	28.88	11.81
M393*	*M. lutarioriparius*	Hunan	Changde	111.96	29.54	13.05
M394	*M. lutarioriparius*	Jiangsu	Hangzhou	120.42	30.31	0.00
M397	*M. lutarioriparius*	Hunan	Yiyang	112.41	28.83	6.08
M398	*M. lutarioriparius*	Hunan	Changde	112.10	28.92	8.08
M399*	*M. lutarioriparius*	Jiangsu	Nantong	120.99	31.91	0.00
M400	*M. lutarioriparius*	Hunan	Changde	111.91	29.62	11.98
M401*	*M. lutarioriparius*	Hunan	Yiyang	112.43	28.83	4.80
M402*	*M. sacchariflorus*	Chongqing	Tongliang	106.11	30.00	0.00
M403	*M. lutarioriparius*	Hunan	Changsha	112.81	28.48	35.71
M405*	*M. lutarioriparius*	Hunan	Anxiang	112.15	29.16	17.82
M406	*M. lutarioriparius*	Hunan	Yueyang	112.70	28.64	19.97
M407	*M. lutarioriparius*	Hunan	Changde	112.16	28.80	8.91
M410	*M. lutarioriparius*	Hunan	Yueyang	113.07	29.43	17.45
M411*	*M. lutarioriparius*	Hunan	Yueyang	113.10	29.44	15.92
Mxg	*M. x giganteus*					

*64 genotypes of mini-core collection developed using the Core Hunter algorithm.

Table S2. Effect of different concentrations of NaCl treatments on the traits of ten *M. sacchariflorus* and *M. lutarioriparius* seedlings.

	GR (cm/day)					NIL (leaf)					LER (cm/day)					Sen				
Genotype	NaCl levels (mM)																			
	0	100	150	200	250	0	100	150	200	250	0	100	150	200	250	0	100	150	200	250
M87	1.45	0.49	0.10	0.29	0.44	3.00	2.67	2.67	1.67	1.33	2.91	1.46	1.39	1.10	0.96	1.00	5.00	5.67	7.00	7.00
M112	0.65	0.31	0.17	0.21	0.18	3.00	2.00	2.33	1.33	0.33	1.54	0.78	0.87	0.58	0.18	1.00	4.33	5.67	7.67	9.00
M129	0.60	0.19	0.55	0.00	0.24	4.00	2.00	2.00	0.67	1.00	0.50	0.41	1.15	0.45	0.52	1.00	5.00	5.00	8.33	8.33
M164	0.93	0.09	0.00	0.00	0.01	4.00	1.67	1.33	0.00	0.33	3.13	0.85	0.00	0.01	0.58	1.00	5.67	9.00	9.00	9.00
M228	1.01	0.40	0.14	0.26	0.42	3.00	2.00	2.33	1.33	1.00	1.31	0.93	0.51	0.83	0.75	1.00	3.67	5.00	5.67	8.33
M229	0.94	0.51	0.60	0.28	0.28	3.00	2.67	2.33	2.33	1.00	2.41	1.04	1.19	1.00	0.71	1.00	3.00	3.00	3.00	7.00
M245	0.53	0.24	0.19	0.00	0.00	2.33	1.00	0.00	0.00	1.00	0.84	0.47	0.23	0.23	0.02	1.00	7.00	8.33	7.67	9.00
M253	0.78	0.16	0.02	0.00	0.00	3.00	1.33	1.50	0.00	0.67	1.75	0.57	0.38	0.14	0.23	1.00	5.00	5.00	8.33	9.00
M275	0.60	0.32	0.01	0.00	0.00	2.67	2.00	0.33	0.67	0.33	0.69	0.68	0.09	0.00	0.30	1.00	3.00	8.33	9.00	7.00
Mxg	0.55	0.13	0.03	0.00	0.04	3.33	1.67	1.33	1.67	0.33	1.02	0.71	0.49	0.84	0.32	1.00	5.00	6.33	6.33	9.00
Mean	0.80	0.29	0.18	0.11	0.16	3.13	1.90	1.62	0.97	0.73	1.61	0.79	0.63	0.52	0.46	1.00	4.67	6.13	7.20	8.27
Min	0.53	0.09	0.00	0.00	0.00	2.33	1.00	0.00	0.00	0.33	0.50	0.41	0.00	0.00	0.02	1.00	3.00	3.00	3.00	7.00
Max	1.45	0.51	0.60	0.29	0.44	4.00	2.67	2.67	2.33	1.33	3.13	1.46	1.39	1.10	0.96	1.00	7.00	9.00	9.00	9.00
Std.dev	0.29	0.15	0.22	0.14	0.18	0.53	0.52	0.90	0.82	0.38	0.93	0.31	0.49	0.41	0.30	0.00	1.23	1.89	1.83	0.91
Coef.var	0.36	0.51	1.21	1.29	1.09	0.17	0.27	0.55	0.85	0.52	0.58	0.39	0.78	0.80	0.65	0.00	0.26	0.31	0.25	0.11

	SWC (%)					RWC (%)					SNC (mg/g)					RNC (mg/g)				
Genotype	NaCl levels (mM)																			
	0	100	150	200	250	0	100	150	200	250	0	100	150	200	250	0	100	150	200	250
M87	45.63	30.71	31.05	20.86	19.14	21.43	25.32	29.77	33.26	23.76	3.16	59.78	90.27	125.77	130.68	6.71	40.37	53.44	80.29	109.18
M112	27.63	22.07	18.08	12.23	5.30	14.01	12.97	20.78	15.43	13.65	3.18	48.42	109.92	127.69	104.46	11.32	41.17	50.99	91.06	127.86
M129	27.29	19.72	15.94	9.16	8.19	23.46	23.32	28.24	17.43	24.00	5.11	43.13	72.62	117.86	92.86	10.56	35.56	48.21	60.29	75.19
M164	45.01	27.00	8.27	5.56	8.68	23.09	19.91	16.68	22.62	13.66	2.51	75.11	70.31	72.94	144.39	6.00	43.85	37.26	62.16	83.34
M228	23.95	17.14	9.48	11.51	7.77	10.23	7.10	5.30	9.71	7.64	2.61	60.23	71.09	132.09	102.07	4.25	47.69	44.13	64.51	106.88
M229	38.76	34.45	31.58	27.71	16.11	18.42	14.43	15.16	17.41	10.94	2.85	29.36	95.06	58.70	94.49	5.59	38.21	39.47	66.95	100.12
M245	15.12	5.17	2.91	7.99	2.03	11.79	8.60	5.87	8.99	8.68	2.60	71.66	59.73	131.65	153.79	11.33	36.79	36.28	83.00	95.23
M253	27.68	9.61	8.28	4.27	2.41	11.27	8.04	12.09	15.15	7.15	2.64	36.98	58.49	84.90	106.76	5.49	43.56	71.93	56.65	104.53
M275	15.47	14.30	4.21	4.66	7.11	9.05	5.15	6.68	6.06	7.75	2.51	31.52	54.50	121.71	45.80	4.32	46.02	48.81	58.39	73.52
Mxg	12.09	8.11	10.21	6.92	3.14	7.30	6.19	6.49	9.19	5.78	3.62	67.00	90.07	146.34	197.25	4.17	49.77	80.08	96.17	133.08
Mean	27.86	18.83	14.00	11.09	7.99	15.01	13.10	14.71	15.53	12.30	3.08	52.32	77.21	111.97	117.25	6.97	42.30	51.06	71.95	100.89
Min	12.09	5.17	2.91	4.27	2.03	7.30	5.15	5.30	6.06	5.78	2.51	29.36	54.50	58.70	45.80	4.17	35.56	36.28	56.65	73.52
Max	45.63	34.45	31.58	27.71	19.14	23.46	25.32	29.77	33.26	24.00	5.11	75.11	109.92	146.34	197.25	11.33	49.77	80.08	96.17	133.08
Std.dev	12.06	9.85	10.22	7.62	5.67	6.08	7.41	9.17	8.02	6.65	0.80	16.74	18.26	29.13	41.33	2.95	4.72	14.48	14.43	20.09
Coef.var	0.43	0.52	0.73	0.69	0.71	0.41	0.57	0.62	0.52	0.54	0.26	0.32	0.24	0.26	0.35	0.42	0.11	0.28	0.20	0.20

Genotype	SN/RN					SKC (mg/g)					RKC (mg/g)					SK/RK				
	NaCl levels (mM)																			
	0	100	150	200	250	0	100	150	200	250	0	100	150	200	250	0	100	150	200	250
M87	0.47	1.48	1.69	1.57	1.20	61.26	58.53	60.07	58.86	80.01	39.92	36.31	34.00	34.02	26.88	1.53	1.61	1.77	1.73	2.98
M112	0.28	1.18	2.16	1.40	0.82	44.02	62.58	59.75	46.73	65.98	15.48	23.41	18.38	19.45	12.92	2.84	2.67	3.25	2.40	5.11
M129	0.48	1.21	1.51	1.95	1.24	54.27	59.98	60.80	57.69	61.88	16.85	28.18	30.30	22.40	22.24	3.22	2.13	2.01	2.57	2.78
M164	0.42	1.71	1.89	1.17	1.73	64.90	59.71	45.54	38.05	68.64	35.97	36.08	34.29	17.33	29.42	1.80	1.66	1.33	2.20	2.33
M228	0.61	1.26	1.61	2.05	0.96	54.57	54.81	54.82	65.10	56.26	24.61	30.43	38.36	23.71	17.78	2.22	1.80	1.43	2.75	3.17
M229	0.51	0.77	2.41	0.88	0.94	49.95	73.83	68.28	71.12	88.39	20.27	29.97	28.95	20.76	31.66	2.46	2.46	2.36	2.75	2.79
M245	0.23	1.95	1.65	1.59	1.61	34.21	66.15	76.40	60.52	56.79	33.67	47.55	42.53	39.97	39.38	1.02	1.39	1.80	1.51	1.44
M253	0.48	0.85	0.81	1.50	1.02	47.63	66.31	63.35	50.76	62.84	27.27	29.40	26.24	27.64	26.57	1.75	2.26	2.41	1.84	2.36
M275	0.58	0.68	1.12	2.08	0.62	43.00	71.69	69.37	58.81	53.45	28.96	27.84	26.32	28.09	14.54	1.48	2.58	2.64	2.09	3.68
Mxg	0.87	1.35	1.12	1.52	1.48	64.74	68.18	68.61	57.91	68.44	24.76	32.68	33.54	22.32	19.50	2.62	2.09	2.05	2.59	3.51
Mean	0.49	1.24	1.60	1.57	1.16	51.85	64.18	62.70	56.55	66.27	26.78	32.19	31.29	25.57	24.09	2.09	2.06	2.10	2.24	3.01
Min	0.23	0.68	0.81	0.88	0.62	34.21	54.81	45.54	38.05	53.45	15.48	23.41	18.38	17.33	12.92	1.02	1.39	1.33	1.51	1.44
Max	0.87	1.95	2.41	2.08	1.73	64.90	73.83	76.40	71.12	88.39	39.92	47.55	42.53	39.97	39.38	3.22	2.67	3.25	2.75	5.11
Std.dev	0.18	0.41	0.49	0.38	0.36	10.06	6.09	8.66	9.35	10.92	8.08	6.63	6.84	7.01	8.26	0.69	0.44	0.58	0.44	0.97
Coef.var	0.36	0.33	0.31	0.24	0.31	0.19	0.09	0.14	0.17	0.16	0.30	0.21	0.22	0.27	0.34	0.33	0.21	0.28	0.20	0.32

Genotype	SK/N					RK/N				
	NaCl levels (mM)									
	0	100	150	200	250	0	100	150	200	250
M87	19.36	0.98	0.67	0.47	0.61	5.95	0.90	0.64	0.42	0.25
M112	13.86	1.29	0.54	0.37	0.63	1.37	0.57	0.36	0.21	0.10
M129	10.61	1.39	0.84	0.49	0.67	1.60	0.79	0.63	0.37	0.30
M164	25.88	0.79	0.65	0.52	0.48	5.99	0.82	0.92	0.28	0.35
M228	20.89	0.91	0.77	0.28	0.55	5.79	0.64	0.87	0.37	0.17
M229	17.51	2.51	0.72	1.11	0.94	3.62	0.78	0.73	0.31	0.32
M245	13.16	0.92	1.28	0.46	0.37	2.97	1.29	1.17	0.48	0.41
M253	18.04	1.79	1.08	0.60	0.59	4.97	0.68	0.36	0.49	0.25
M275	17.13	2.27	1.27	0.48	1.17	6.71	0.60	0.54	0.48	0.20
Mxg	17.88	1.02	0.76	0.40	0.35	5.94	0.66	0.42	0.23	0.15
Mean	17.43	1.39	0.86	0.52	0.63	4.49	0.77	0.66	0.36	0.25
Min	10.61	0.79	0.54	0.28	0.35	1.37	0.57	0.36	0.21	0.10
Max	25.88	2.51	1.28	1.11	1.17	6.71	1.29	1.17	0.49	0.41
Std.dev	4.28	0.61	0.26	0.23	0.25	1.96	0.21	0.26	0.10	0.10
Coef.var	0.25	0.44	0.31	0.44	0.39	0.44	0.27	0.40	0.28	0.39

GR: Shoot growth rate, NIL: leaves increased number, LER: leaf expansion rate, Sen: leaf senescence scale, SWC: shoot water content, RWC: root water content, SNC: shoot Na^+ concentration, RNC: root Na^+ concentration, SN/RN: the ratio of shoot Na^+ concentration to root Na^+ concentration, SKC: shoot K^+ concentration, RKC: root K^+ concentration, SK/RK: the ratio of shoot K^+ concentration to root K^+ concentration, SK/N: the ratio of shoot K^+ concentration to shoot Na^+ concentration, RK/N: the ratio of root K^+ concentration to root Na^+ concentration.

Table S3. Salt-injury index traits of ten *M. sacchariflorus* and *M. lutarioriparius* seedlings under different NaCl stresses.

Genotype	SII of GR				SII of NIL				SII of LER			
	NaCl concentration (mM)											
	100	150	200	250	100	150	200	250	100	150	200	250
M87	0.66	0.93	0.80	0.70	0.11	0.11	0.44	0.56	0.50	0.52	0.62	0.67
M112	0.52	0.74	0.67	0.73	0.33	0.22	0.56	0.89	0.49	0.44	0.62	0.88
M129	0.68	0.08	0.99	0.60	0.50	0.50	0.83	0.75	0.17	-1.32	0.10	-0.05
M164	0.90	1.00	1.00	0.99	0.58	0.67	1.00	0.92	0.73	1.00	1.00	0.81
M228	0.60	0.86	0.75	0.58	0.33	0.22	0.56	0.67	0.29	0.61	0.36	0.43
M229	0.46	0.36	0.70	0.70	0.11	0.22	0.22	0.67	0.57	0.51	0.59	0.71
M245	0.55	0.63	1.00	1.00	0.57	1.00	1.00	0.57	0.44	0.73	0.73	0.98
M253	0.79	0.98	1.00	1.00	0.56	0.50	1.00	0.78	0.67	0.78	0.92	0.87
M275	0.46	0.98	1.00	1.00	0.25	0.88	0.75	0.88	0.01	0.86	0.99	0.57
Mxg	0.76	0.95	1.00	0.92	0.50	0.60	0.50	0.90	0.30	0.52	0.17	0.69
Mean	0.64	0.75	0.89	0.82	0.38	0.49	0.69	0.76	0.42	0.46	0.61	0.65

SII of GR: the salt-injury index (SII) of shoot growth rate (GR), SII of NIL: the salt-injury index of leaves increased number (NIL), SII of LER: the salt-injury index of leaf expansion rate (LER).

Table S4. The salt tolerance-related traits in 318 *M. sacchariflorus* and *M. lutarioriparius* genotypes.

Genotype	GR_CK (cm/day)	GR_S (cm/day)	RGR	INL_CK (leaf)	INL_S (leaf)	RINL	LER_CK (cm/day)	LER_S (cm/day)	RLER	SWC (%)	RWC (%)	Sen	SNC (mg/g)	RNC (mg/g)	SN/RN	SKC (mg/g)	RKC (mg/g)	SK/RK	SK/N	RK/N
M1	0.41	0.21	0.50	2.67	1.89	0.72	1.41	0.74	0.56	23.86	12.22	5.67	121.09	34.16	3.56	74.94	23.56	3.18	0.62	0.70
M3	0.38	0.11	0.45	2.33	1.44	0.61	1.18	0.83	0.70	17.36	8.36	5.44	89.06	39.51	2.44	58.79	20.49	2.92	0.69	0.54
M4	0.20	0.17	0.76	2.00	0.78	0.46	0.99	0.72	0.69	11.60	9.62	6.11	131.31	45.08	3.08	61.95	24.12	2.88	0.57	0.52
M5	0.16	0.02	0.23	0.94	0.11	0.11	0.77	0.17	0.26	9.27	8.24	8.33	128.89	40.72	3.29	81.64	20.24	4.55	0.70	0.48
M7	0.20	0.10	0.53	1.89	0.78	0.44	0.89	0.53	0.69	12.22	10.05	7.89	156.08	41.94	3.90	66.40	29.87	2.23	0.43	0.74
M10	0.53	0.06	0.08	1.67	0.44	0.22	1.12	0.42	0.34	16.53	10.65	8.78	160.62	41.41	3.88	76.69	26.28	2.87	0.46	0.63
M11	0.27	0.10	0.51	2.33	1.00	0.43	1.06	0.66	0.64	9.22	8.83	6.33	101.37	27.11	4.06	58.77	26.58	2.25	0.64	1.05
M13	0.23	0.02	0.24	1.78	0.89	0.50	0.83	0.30	0.50	13.29	13.51	7.44	183.76	40.38	4.75	75.90	19.76	3.84	0.42	0.52
M14*	0.23	0.14	0.73	2.67	1.56	0.61	1.16	0.77	0.91	15.83	11.12	6.56	130.56	29.78	4.60	63.00	22.88	2.82	0.48	0.80
M17	0.35	0.19	0.49	2.44	1.00	0.38	1.43	0.63	0.43	14.22	11.85	5.67	147.73	50.20	3.25	75.03	30.99	2.94	0.59	0.59
M18	0.38	0.33	0.63	2.56	1.11	0.41	0.82	0.58	0.61	9.89	3.57	6.56	137.17	66.61	2.55	65.28	24.41	2.82	0.64	0.38
M19	0.57	0.15	0.24	2.78	0.89	0.34	0.91	0.43	0.46	15.80	11.58	6.33	116.78	39.00	3.00	44.32	27.22	1.73	0.39	0.70
M20*	0.40	0.13	0.28	2.22	0.44	0.25	0.83	0.47	0.60	13.32	12.87	8.11	140.95	52.87	2.88	63.15	21.69	3.13	0.47	0.44
M21	0.49	0.04	0.14	2.78	0.67	0.23	1.52	0.64	0.43	15.69	12.37	7.00	146.74	41.71	3.57	70.07	30.58	2.43	0.48	0.77
M22	0.30	0.14	0.52	2.22	0.78	0.34	1.07	0.69	0.66	16.76	14.01	5.44	87.24	41.09	2.30	71.84	32.31	2.25	0.83	0.85
M24	0.29	0.27	0.75	2.78	1.44	0.52	1.46	0.77	0.55	16.76	10.32	4.78	88.72	43.17	2.15	65.09	19.26	3.95	0.74	0.42
M25	0.34	0.06	0.21	2.33	0.78	0.31	1.17	0.41	0.33	11.63	7.60	6.33	159.69	45.10	3.83	58.86	35.24	1.80	0.40	0.81
M27	0.48	0.14	0.22	1.83	0.89	0.44	1.14	0.77	0.75	18.91	11.44	6.78	134.88	55.42	2.66	53.73	32.79	1.74	0.41	0.60
M28	0.32	0.35	0.99	2.33	1.17	0.45	1.30	1.07	0.72	17.19	8.97	4.22	80.46	63.85	1.53	65.88	31.21	2.19	0.85	0.53
M29*	0.35	0.28	1.02	2.28	1.78	0.78	1.04	0.95	0.87	16.81	7.56	3.67	76.09	57.04	1.35	67.23	24.22	3.02	0.90	0.41
M30	0.22	0.09	0.30	2.44	0.89	0.36	0.75	0.48	0.58	7.77	5.01	6.11	120.25	64.18	2.04	66.50	27.18	2.85	0.61	0.41
M31	0.55	0.30	0.56	2.22	1.89	0.88	1.23	0.97	0.89	14.63	7.75	4.11	85.46	73.90	1.17	74.07	25.94	2.91	0.88	0.35
M32*	0.22	0.06	0.18	2.11	0.89	0.40	1.04	0.43	0.39	7.14	6.30	7.44	154.56	59.99	2.58	57.31	21.42	2.67	0.42	0.40
M33	0.27	0.14	0.65	2.22	0.89	0.42	1.07	0.65	0.61	17.64	15.50	7.89	161.81	45.97	3.55	73.78	28.94	2.67	0.45	0.62
M34	0.55	0.22	0.37	2.33	1.22	0.52	1.35	0.76	0.58	23.73	14.09	5.44	99.68	35.44	2.89	66.64	22.01	3.20	0.68	0.66
M35*	0.87	0.11	0.10	2.56	1.00	0.37	1.99	0.71	0.38	16.17	12.68	6.11	124.62	49.62	2.88	67.45	27.21	2.71	0.55	0.56
M37*	0.74	0.57	0.69	2.89	1.17	0.40	1.99	1.63	0.76	21.56	10.77	4.78	87.26	67.49	1.52	73.42	24.39	3.37	0.85	0.37
M38	0.61	0.23	0.34	2.89	1.33	0.47	1.59	0.97	0.65	19.65	13.17	6.33	143.43	43.46	3.74	76.26	23.89	3.38	0.63	0.55
M39	0.55	0.31	0.66	2.22	0.67	0.29	1.92	0.90	0.50	15.86	8.80	7.22	141.72	55.59	2.82	60.13	20.53	3.12	0.43	0.38
M40	0.75	0.23	0.56	2.11	1.00	0.48	1.52	0.71	0.47	21.50	18.48	6.11	134.73	47.62	3.08	51.70	29.25	2.01	0.39	0.59
M42	0.26	0.19	0.73	2.67	1.67	0.63	1.49	0.97	0.65	13.09	10.96	5.22	101.25	54.88	1.95	75.69	17.80	4.82	0.75	0.31
M43	0.37	0.23	0.65	2.33	1.33	0.57	1.15	1.02	0.95	13.49	9.21	5.22	87.17	62.98	1.51	73.34	21.86	3.73	0.89	0.36
M45*	0.96	0.19	0.21	3.00	1.33	0.46	2.11	0.88	0.40	26.39	12.29	5.44	74.32	40.31	1.79	80.19	25.65	3.17	1.23	0.64
M46	0.34	0.05	0.21	2.67	1.56	0.58	1.13	0.99	0.87	19.13	14.28	5.89	131.89	50.19	2.90	55.08	36.33	1.72	0.42	0.73
M47	0.51	0.06	0.10	3.00	0.67	0.24	1.47	0.44	0.26	11.48	10.14	7.44	130.15	32.96	4.38	67.30	22.40	3.07	0.53	0.69
M50	0.15	0.09	0.61	1.78	1.00	0.58	0.71	0.60	0.83	17.77	11.08	5.56	135.32	47.90	2.96	78.50	18.95	4.18	0.61	0.40
M51	0.29	0.11	0.34	2.89	1.22	0.43	1.16	0.69	0.66	16.07	13.54	6.56	133.82	50.12	2.93	71.29	30.42	2.44	0.57	0.67
M55	0.49	0.17	0.41	2.67	1.22	0.43	1.41	0.83	0.61	19.22	17.11	5.44	119.85	49.49	2.96	72.60	29.69	2.68	0.60	0.64
M57	0.48	0.14	0.37	2.44	0.67	0.31	1.06	0.47	0.46	16.92	10.36	6.78	120.91	37.21	3.62	61.84	19.11	3.23	0.61	0.51
M59	0.55	0.21	0.21	2.22	1.44	0.67	1.46	1.28	0.92	15.86	7.89	4.78	103.62	70.33	1.58	59.54	27.99	2.32	0.65	0.40

M60	0.40	0.15	0.45	1.67	1.11	0.67	1.26	1.01	0.83	17.64	11.75	5.44	78.12	34.91	2.27	68.80	28.54	2.48	0.91	0.87
M63	0.28	0.05	0.37	2.44	1.67	0.69	1.10	0.65	0.87	10.94	5.66	5.44	110.20	57.58	2.17	68.74	27.23	2.98	0.63	0.47
M66*	0.22	0.12	0.97	0.78	0.33	0.44	0.59	0.26	0.42	10.68	8.07	8.22	123.52	49.71	2.46	65.13	18.75	3.51	0.55	0.38
M67	0.31	0.24	0.75	2.56	1.33	0.52	0.79	0.73	0.94	14.17	9.71	5.22	104.02	57.63	2.10	71.96	25.31	3.51	0.69	0.42
M69*	0.20	0.04	1.11	2.44	0.67	0.31	1.24	0.51	0.61	19.21	14.74	7.22	132.50	34.62	3.83	68.07	31.05	2.26	0.51	0.90
M73	0.30	0.20	0.62	2.22	1.00	0.43	1.31	0.66	0.49	10.07	9.25	6.78	176.52	57.22	3.48	75.28	30.18	2.88	0.47	0.52
M74*	0.26	0.04	0.21	1.89	0.89	0.44	0.87	0.62	0.88	10.06	7.45	6.44	122.26	58.75	2.77	66.14	28.06	2.66	0.59	0.47
M76	0.34	0.14	0.57	2.00	0.78	0.36	1.00	0.47	0.49	8.93	9.02	7.89	199.06	56.50	4.01	68.70	29.59	2.92	0.35	0.49
M77	0.50	0.16	0.35	2.11	1.00	0.48	1.36	0.69	0.55	14.56	12.95	5.00	99.24	48.27	2.24	61.20	28.13	2.35	0.62	0.59
M79	0.67	0.30	0.47	2.44	0.78	0.33	1.32	0.55	0.46	18.83	13.41	5.67	97.54	35.81	2.91	57.87	27.07	2.18	0.63	0.76
M85	0.16	0.03	0.19	1.67	0.44	0.29	0.98	0.24	0.27	12.26	12.01	7.67	146.25	38.20	4.22	69.44	25.66	3.32	0.46	0.62
M86	0.30	0.34	1.16	2.00	1.00	0.46	1.38	0.77	0.58	17.21	17.25	5.89	107.26	35.74	3.16	59.87	23.58	2.65	0.57	0.65
M87	0.43	0.20	0.49	2.33	1.00	0.33	1.14	0.63	0.65	15.09	12.20	7.67	148.82	57.50	2.87	61.18	22.70	2.95	0.42	0.40
M88*	0.24	0.13	0.39	2.00	0.89	0.44	1.29	0.61	0.42	9.79	7.24	7.89	220.31	53.80	4.53	70.76	22.58	3.26	0.35	0.43
M89	0.43	0.13	0.35	2.22	1.33	0.55	1.39	0.85	0.55	14.05	8.59	6.78	118.12	57.95	2.89	71.91	30.26	2.90	0.67	0.54
M90	0.63	0.30	0.78	2.33	1.00	0.46	1.59	1.11	0.81	12.78	12.98	5.00	98.37	54.79	1.93	54.86	18.50	3.15	0.67	0.36
M92	0.35	0.12	0.22	2.00	1.33	0.64	0.97	0.72	0.89	10.98	6.95	5.89	118.21	54.95	2.56	77.47	20.63	5.47	0.65	0.40
M96	0.21	0.13	0.74	2.56	1.67	0.63	1.42	0.79	0.57	12.93	5.34	4.78	111.85	38.64	3.49	61.66	22.48	3.21	0.59	0.57
M99	0.33	0.06	0.14	2.56	1.22	0.49	1.63	0.85	0.56	14.48	12.42	7.00	154.97	50.32	3.29	64.03	31.31	2.28	0.43	0.66
M100*	0.25	0.11	0.46	1.67	0.67	0.40	1.00	0.65	0.65	10.82	6.61	6.78	102.28	40.77	2.49	63.79	38.14	1.77	0.66	0.97
M101	0.37	0.35	0.95	2.78	1.11	0.38	1.60	0.86	0.52	17.36	13.92	6.56	157.18	58.27	2.94	65.27	27.65	2.62	0.42	0.50
M102	0.48	0.09	0.20	2.44	0.78	0.31	1.36	0.74	0.54	15.21	12.06	6.78	140.48	43.01	3.45	58.86	35.36	1.76	0.42	0.83
M103	0.23	0.17	0.80	2.00	1.33	0.67	1.05	0.71	0.68	13.43	8.50	6.33	134.59	33.67	4.08	67.14	22.10	3.19	0.51	0.65
M104	0.50	0.11	0.23	2.56	1.56	0.59	1.61	1.00	0.51	12.65	9.65	6.33	136.58	51.97	3.22	65.42	17.42	4.53	0.48	0.33
M106	0.41	0.23	0.53	1.44	0.67	0.44	0.98	0.71	0.77	14.64	10.21	6.33	111.96	50.76	2.50	76.39	21.86	3.99	0.80	0.43
M107	0.53	0.13	0.34	2.44	0.78	0.26	1.24	0.55	0.55	9.63	10.30	7.22	115.47	33.65	3.48	76.18	21.11	3.59	0.66	0.65
M108*	0.43	0.13	0.26	2.33	0.44	0.22	0.89	0.44	0.54	15.08	12.28	7.22	167.25	49.80	3.67	68.12	37.03	1.84	0.41	0.82
M109	0.45	0.37	0.75	2.22	1.56	0.74	0.87	0.65	1.09	11.64	7.23	6.78	154.62	35.62	4.49	68.99	28.26	2.61	0.45	0.80
M110	0.54	0.30	0.65	2.33	0.89	0.39	1.28	0.72	0.67	19.85	11.42	5.89	127.51	26.46	4.85	71.34	27.33	2.74	0.61	1.06
M112	0.27	0.21	0.65	1.89	1.00	0.56	1.11	0.73	0.73	18.49	9.74	5.67	116.48	41.49	3.08	66.87	26.70	2.55	0.62	0.72
M113	0.30	0.12	0.36	2.56	1.78	0.71	1.25	0.67	0.50	14.43	7.80	5.00	95.57	46.05	2.07	80.42	14.37	6.03	0.85	0.31
M115	0.42	0.24	0.64	1.78	1.39	0.76	0.71	0.54	0.83	10.73	5.78	5.11	92.57	39.26	2.49	64.35	25.37	2.58	0.71	0.72
M116*	0.10	0.14	1.57	2.22	1.11	0.49	1.03	0.62	0.64	14.10	8.48	6.33	91.51	36.17	2.55	53.88	27.85	1.96	0.63	0.77
M117	0.31	0.21	0.71	2.56	1.78	0.69	1.03	0.78	0.82	15.22	5.26	5.67	103.85	41.89	2.55	55.09	21.96	2.61	0.53	0.52
M118	0.20	0.14	0.62	1.56	1.11	0.64	0.98	0.65	0.53	20.08	12.10	5.33	106.68	38.48	2.71	68.19	22.11	3.08	0.78	0.60
M119	0.34	0.20	0.44	2.44	1.33	0.55	0.91	0.60	0.66	12.46	7.39	6.56	117.45	40.12	3.01	61.81	22.38	3.22	0.54	0.61
M120	0.49	0.31	0.65	2.56	1.22	0.47	1.27	0.86	0.70	22.39	13.94	6.11	125.39	46.60	3.11	63.79	31.06	2.07	0.51	0.76
M121	0.36	0.16	0.58	2.89	1.56	0.53	0.99	0.53	0.50	14.08	11.97	5.67	90.81	29.41	3.39	56.41	27.50	2.25	0.62	0.96
M122*	0.33	0.08	0.23	2.11	0.44	0.17	1.40	0.38	0.29	9.49	9.19	8.33	212.64	63.39	3.36	104.75	26.42	4.50	0.49	0.42
M123*	0.41	0.04	0.18	2.44	0.11	0.08	1.05	0.26	0.25	4.37	8.08	8.78	158.98	56.71	3.21	71.27	14.06	5.06	0.45	0.26
M124	0.44	0.12	0.31	2.22	1.33	0.77	1.13	0.66	0.62	23.55	21.10	6.11	97.78	29.19	3.40	58.14	26.36	2.35	0.60	0.95
M125	0.42	0.16	0.40	2.06	1.33	0.63	1.03	0.58	0.59	14.77	11.35	5.67	104.25	33.11	3.31	67.82	23.87	3.11	0.73	0.72
M126	0.42	0.15	0.49	2.44	0.89	0.35	1.80	0.77	0.46	16.01	17.84	7.44	185.07	45.96	4.14	59.06	27.38	2.33	0.33	0.59

M127	0.67	0.17	0.21	2.44	1.44	0.59	1.00	0.66	0.66	17.22	15.03	7.00	132.74	49.86	2.75	66.27	25.42	2.79	0.50	0.50
M128	0.61	0.28	0.98	2.78	1.56	0.54	2.46	1.49	0.59	19.51	10.76	4.78	86.90	52.47	1.71	57.64	33.41	1.76	0.68	0.66
M129	0.64	0.19	0.30	3.33	1.22	0.36	1.08	0.40	0.45	15.95	11.34	6.33	89.41	26.15	3.43	78.26	25.71	3.50	0.88	0.99
M130	0.27	0.15	0.30	2.44	1.33	0.53	0.89	0.77	0.88	19.00	14.03	6.33	109.26	30.50	3.63	60.87	18.89	3.52	0.58	0.60
M131	0.38	0.11	0.30	2.56	1.00	0.30	1.01	0.49	0.46	11.34	6.67	7.22	157.68	38.13	4.18	81.33	25.17	3.33	0.54	0.68
M132	0.36	0.12	1.12	2.56	1.56	0.68	1.28	0.83	0.75	21.95	19.42	5.22	136.35	41.98	3.49	55.68	34.45	1.79	0.41	0.84
M133	0.36	0.16	0.35	1.89	1.22	0.64	1.50	1.00	0.66	14.18	11.65	7.22	150.53	50.94	3.04	73.39	26.24	2.81	0.52	0.53
M134	0.41	0.24	0.71	2.33	1.56	0.71	1.48	1.40	0.96	25.76	15.40	5.22	124.15	44.30	3.18	57.87	30.37	1.92	0.46	0.75
M135*	0.26	0.19	1.32	2.44	1.67	0.67	1.61	1.01	0.65	19.97	10.68	4.11	68.52	35.75	1.97	72.04	21.87	3.46	1.07	0.61
M136	0.42	0.35	0.90	2.67	1.44	0.55	1.35	1.13	0.88	18.22	9.04	3.89	96.52	72.87	1.43	58.21	20.01	3.04	0.67	0.30
M137	0.22	0.11	0.45	2.56	0.89	0.26	1.00	0.38	0.55	13.05	12.77	7.67	162.26	53.71	3.42	70.95	23.86	3.62	0.44	0.45
M138	0.29	0.08	0.51	2.56	1.00	0.38	1.36	0.47	0.34	16.39	7.77	7.22	113.98	35.43	3.21	64.96	34.02	2.07	0.59	0.96
M139*	0.48	0.22	0.56	2.00	1.44	0.75	1.67	0.91	0.63	16.62	7.01	4.11	100.67	63.58	1.66	72.87	17.59	4.80	0.80	0.27
M140	0.42	0.08	0.21	2.17	0.33	0.15	1.25	0.46	0.44	14.15	14.60	7.67	114.45	35.50	3.26	70.29	28.32	2.56	0.61	0.81
M141	0.23	0.02	0.13	2.44	0.67	0.28	1.05	0.59	0.66	9.76	12.42	7.89	130.47	52.90	2.87	67.09	16.37	4.26	0.52	0.34
M142	0.33	0.02	0.05	2.44	0.44	0.18	1.17	0.32	0.24	8.54	7.42	8.11	177.52	60.23	3.04	77.91	28.28	2.76	0.46	0.48
M143	0.37	0.21	0.61	2.22	1.44	0.63	1.23	1.01	0.84	16.52	6.23	5.89	106.57	58.18	2.02	66.59	20.66	3.80	0.62	0.35
M144	0.24	0.11	0.48	2.56	1.11	0.43	1.15	0.66	0.58	13.71	12.46	6.56	148.66	46.70	3.59	64.78	19.73	3.47	0.44	0.43
M145	0.43	0.16	0.42	1.89	1.22	0.64	1.41	0.96	0.78	14.10	8.00	5.22	104.47	49.07	2.14	76.17	22.56	3.39	0.75	0.46
M146	0.35	0.33	0.63	2.11	1.11	0.52	1.51	0.79	0.54	13.05	8.85	5.44	101.56	47.16	2.41	64.74	23.72	3.01	0.65	0.51
M147	0.66	0.12	0.22	2.89	1.11	0.40	1.43	0.79	0.70	19.28	11.62	5.44	135.10	72.57	2.19	62.87	23.48	2.68	0.48	0.36
M148	0.62	0.24	0.52	2.33	1.44	0.70	1.30	1.05	0.70	14.53	7.15	4.78	88.57	50.27	2.36	77.36	21.94	4.83	0.91	0.48
M149*	0.57	0.28	0.45	2.44	1.44	0.62	1.72	1.31	0.83	15.72	12.61	5.00	111.37	57.10	2.26	62.31	21.37	3.53	0.58	0.38
M150	0.20	0.11	0.62	1.78	0.89	0.53	0.84	0.65	0.81	13.70	8.39	5.44	106.91	51.66	2.37	58.39	25.62	2.52	0.55	0.50
M151	0.33	0.10	0.35	2.11	0.89	0.40	0.96	0.60	0.78	9.58	5.22	7.00	131.14	51.46	2.74	71.26	21.52	3.71	0.59	0.41
M152	0.39	0.11	0.25	2.22	1.11	0.50	1.30	0.72	0.60	8.51	9.62	6.56	139.14	57.17	2.93	59.22	22.33	2.90	0.43	0.39
M153*	0.53	0.42	0.72	2.11	1.56	0.76	1.09	0.96	0.96	11.62	6.12	5.67	142.38	52.05	2.83	69.17	16.56	4.85	0.49	0.31
M154	0.33	0.12	0.29	2.00	1.00	0.48	1.15	0.75	0.62	14.58	11.06	5.67	98.23	52.02	2.09	67.13	23.55	3.02	0.78	0.45
M155	0.43	0.18	0.41	2.00	0.67	0.27	1.38	0.84	0.55	11.55	7.52	6.11	137.74	64.34	2.74	57.57	18.33	3.82	0.44	0.29
M156	0.49	0.06	0.14	2.56	1.22	0.45	1.48	1.01	0.77	17.17	6.85	4.78	121.66	54.57	2.59	67.36	27.16	2.60	0.57	0.50
M157	0.33	0.08	0.25	2.67	1.33	0.49	1.12	0.72	0.70	12.36	8.68	5.89	131.04	55.93	2.50	69.33	26.04	3.38	0.57	0.44
M158	0.28	0.09	0.24	1.67	0.56	0.48	0.88	0.49	0.60	9.83	10.00	7.89	158.03	47.92	3.54	57.78	31.03	2.21	0.39	0.65
M159*	0.08	0.05	0.45	1.67	0.33	0.17	0.78	0.40	0.46	6.90	8.98	7.67	125.87	29.25	4.46	48.48	21.28	2.55	0.43	0.71
M160	0.52	0.24	0.43	2.44	1.11	0.42	1.11	0.65	0.58	9.99	7.03	7.22	120.82	30.66	3.96	50.87	22.18	2.69	0.44	0.72
M161	0.52	0.12	0.26	2.78	0.67	0.23	1.51	0.48	0.43	10.52	6.36	7.44	186.30	65.31	2.96	74.36	23.21	3.71	0.41	0.35
M162	0.27	0.25	1.04	2.22	1.11	0.47	1.69	0.96	0.59	17.34	12.43	6.33	139.72	49.05	2.89	68.57	30.40	2.37	0.49	0.61
M164	0.40	0.18	0.53	2.11	0.78	0.38	0.96	0.34	0.40	10.96	8.03	7.89	112.44	33.17	3.66	73.64	20.77	3.79	0.65	0.72
M165*	0.21	0.13	0.69	2.56	1.33	0.52	1.47	0.85	0.61	13.75	9.31	6.11	115.13	44.48	2.57	81.19	17.61	4.91	0.76	0.44
M166*	0.39	0.15	0.48	3.11	0.89	0.29	1.25	0.72	0.72	17.29	15.07	6.56	128.63	43.34	3.22	75.43	29.37	2.64	0.59	0.67
M167*	0.16	0.02	0.07	2.11	1.56	0.69	1.30	0.49	0.35	9.61	8.29	6.56	139.33	70.35	2.67	81.12	20.72	4.28	0.64	0.32
M168*	0.25	0.19	0.82	2.11	1.22	0.59	0.76	0.36	0.44	8.14	6.79	6.00	95.39	36.55	2.58	63.02	22.20	2.86	0.74	0.65
M169	0.21	0.11	0.41	1.44	0.89	0.61	0.82	0.53	0.64	10.15	9.20	7.44	146.57	38.07	3.72	55.68	20.65	2.69	0.43	0.56
M170	0.25	0.06	0.20	2.67	1.11	0.40	1.08	0.75	0.71	11.89	7.34	6.78	131.06	54.75	2.91	68.46	24.85	3.11	0.60	0.52

M171	0.31	0.10	0.56	2.33	1.00	0.43	0.90	0.64	0.74	15.09	7.85	6.11	113.77	34.15	3.37	72.35	16.71	4.35	0.64	0.50
M172	0.41	0.12	0.44	2.78	1.00	0.40	1.10	0.42	0.54	10.02	8.55	8.11	165.24	41.24	4.44	67.07	21.84	3.32	0.41	0.60
M173	0.36	0.18	0.51	2.22	1.11	0.57	1.24	0.71	0.42	13.12	7.70	5.89	87.04	27.80	3.11	58.15	23.99	2.51	0.66	0.90
M174	0.24	0.22	1.27	2.00	1.00	0.44	0.92	0.73	0.79	12.32	7.20	4.78	85.62	43.08	2.50	70.25	22.72	3.50	0.88	0.57
M175	0.21	0.17	1.03	2.56	0.89	0.35	1.09	0.53	0.59	9.75	10.44	6.78	113.43	31.66	3.54	58.67	17.82	3.27	0.55	0.57
M176	0.31	0.20	0.60	1.78	1.33	0.69	1.28	0.83	0.62	14.82	9.04	6.56	138.55	46.62	3.09	68.84	26.84	2.68	0.52	0.57
M177*	0.13	0.10	1.09	2.44	1.22	0.49	0.80	0.62	0.77	11.86	13.28	5.89	102.99	27.30	3.75	47.65	28.81	1.72	0.47	1.07
M179	0.29	0.24	0.86	2.44	1.56	0.64	1.28	1.02	0.81	16.60	8.02	5.00	78.70	62.10	1.37	75.34	27.66	3.35	1.11	0.44
M181	0.41	0.14	0.35	2.22	0.89	0.46	1.05	0.60	0.60	18.90	12.65	5.44	143.89	40.66	3.86	67.81	26.80	3.36	0.47	0.66
M183	0.68	0.56	0.82	2.78	1.11	0.41	1.68	1.37	0.87	20.42	18.35	5.22	123.51	53.95	2.38	62.22	25.46	2.57	0.53	0.48
M185	0.37	0.06	0.19	2.67	0.89	0.37	1.30	0.56	0.42	15.03	11.61	7.67	126.28	55.90	2.60	67.40	28.39	2.64	0.53	0.52
M187	0.23	0.07	0.49	2.00	0.78	0.42	1.25	0.74	0.58	12.31	9.44	6.33	105.02	45.84	2.51	88.16	23.06	4.18	0.87	0.52
M189*	0.44	0.10	0.26	2.11	0.89	0.39	1.35	0.55	0.42	19.43	16.56	7.44	161.43	47.71	3.38	72.97	29.49	2.49	0.45	0.64
M190	0.48	0.13	0.42	1.94	0.56	0.30	1.23	0.45	0.48	16.69	14.42	8.11	138.00	49.92	3.12	76.17	36.50	2.76	0.57	0.73
M191	0.33	0.12	0.24	2.22	1.44	0.60	1.48	0.68	0.47	11.70	8.80	7.44	152.43	58.23	3.00	68.66	19.05	3.64	0.46	0.37
M192*	0.32	0.06	0.12	1.78	1.11	0.69	0.64	0.43	0.67	8.55	6.22	4.78	53.20	48.21	1.19	61.29	28.16	2.79	1.33	0.55
M193	0.12	0.10	0.74	2.22	0.89	0.38	1.19	0.60	0.47	17.30	17.03	6.33	103.11	32.73	3.14	64.08	21.59	2.99	0.66	0.66
M195	0.61	0.20	0.49	2.22	1.11	0.54	1.19	0.64	0.59	12.00	6.28	5.22	124.43	46.35	2.76	74.60	20.87	4.52	0.76	0.45
M196	0.23	0.11	0.78	1.67	0.56	0.33	0.85	0.35	0.55	13.17	12.02	7.22	114.19	25.58	4.65	70.20	26.69	2.64	0.63	1.08
M198	0.39	0.06	0.20	2.56	0.89	0.34	1.32	0.49	0.37	12.13	9.51	7.22	175.74	53.17	3.73	72.98	31.95	2.83	0.44	0.59
M199	0.44	0.26	0.73	2.44	1.56	0.69	1.28	0.86	0.68	22.31	18.77	5.67	131.20	32.26	4.08	70.34	27.28	2.66	0.57	0.85
M201	0.80	0.35	0.46	2.78	1.22	0.44	1.99	1.10	0.54	18.69	8.94	5.22	112.37	71.15	1.85	70.06	29.02	2.51	0.65	0.46
M203*	0.16	0.04	0.22	1.22	0.78	0.64	0.61	0.18	0.30	5.49	5.11	7.22	128.70	39.01	3.55	73.10	12.83	5.96	0.58	0.35
M204	0.34	0.17	0.48	3.11	1.44	0.45	1.37	0.76	0.58	18.60	11.53	6.33	114.82	32.27	3.62	71.47	23.52	3.20	0.63	0.73
M205*	0.26	0.10	0.66	1.56	1.44	0.94	0.63	0.58	0.94	23.16	18.08	6.11	107.95	32.49	3.33	61.44	33.12	1.87	0.58	1.02
M206	0.15	0.10	0.70	2.22	0.67	0.30	1.09	0.52	0.50	12.31	10.00	7.00	108.55	48.17	2.31	78.98	15.93	4.92	0.73	0.34
M207	0.48	0.13	0.36	2.06	0.89	0.48	1.23	0.46	0.33	14.80	11.82	6.56	98.14	33.07	3.25	68.93	23.70	2.98	0.72	0.74
M209	0.24	0.21	1.25	2.72	1.78	0.61	1.01	0.75	0.78	19.54	12.44	5.89	102.55	31.01	3.58	60.58	29.59	2.21	0.59	0.95
M210	0.55	0.03	0.07	2.67	0.56	0.22	1.77	0.67	0.47	14.48	11.34	7.44	141.88	44.48	3.84	70.66	26.71	2.65	0.50	0.70
M211	0.45	0.09	0.21	2.33	1.89	0.81	0.77	0.41	0.49	13.51	12.07	6.33	139.08	40.92	3.58	67.43	24.37	2.85	0.49	0.60
M212	0.38	0.11	0.28	2.17	0.67	0.31	1.58	0.57	0.36	14.27	9.07	6.67	157.27	49.96	3.26	79.66	25.58	3.21	0.53	0.51
M214	0.40	0.12	0.57	2.44	1.22	0.49	1.54	0.53	0.52	15.95	11.65	7.44	191.55	46.41	4.29	69.86	28.36	2.52	0.37	0.66
M215	0.65	0.35	0.68	2.44	0.78	0.36	1.55	0.52	0.33	23.11	16.41	5.67	97.00	31.79	3.13	54.65	19.89	2.73	0.56	0.64
M216	0.34	0.06	0.16	2.44	1.56	0.56	1.03	0.50	0.54	11.96	7.81	5.67	108.03	38.43	3.08	78.36	18.15	4.43	0.75	0.49
M217	0.48	0.22	0.53	2.00	1.11	0.57	0.90	0.63	0.75	13.27	8.63	6.11	135.23	32.89	4.28	81.96	25.70	3.29	0.63	0.81
M220	0.42	0.17	0.41	1.78	1.67	0.94	1.04	0.82	0.78	22.41	18.08	6.33	123.94	34.95	3.56	67.23	26.76	2.63	0.56	0.78
M221	0.34	0.17	0.44	2.78	1.22	0.49	1.51	0.68	0.69	19.74	14.64	5.22	119.65	42.58	3.22	70.24	27.21	2.86	0.59	0.72
M223	0.56	0.12	0.21	2.33	1.00	0.49	1.50	0.65	0.46	18.51	14.85	7.00	137.83	44.40	3.14	85.42	29.62	2.92	0.61	0.70
M224	0.34	0.18	0.52	2.22	0.67	0.29	1.06	0.61	0.61	11.50	8.18	6.78	142.94	66.43	2.23	72.08	32.19	2.46	0.54	0.48
M225	0.30	0.13	0.57	2.33	1.78	0.79	1.45	1.00	0.73	24.32	19.53	3.67	98.27	55.06	2.10	53.76	26.80	2.16	0.55	0.51
M226	0.06	0.04	0.73	1.56	0.78	0.53	0.70	0.69	0.92	11.01	8.15	6.33	103.44	39.83	3.12	57.63	20.75	3.34	0.56	0.50
M227	0.52	0.09	0.18	2.33	1.00	0.43	1.47	1.14	0.77	18.62	11.52	5.67	109.93	40.48	2.73	74.66	27.78	2.72	0.69	0.68
M228*	0.27	0.21	0.77	2.33	1.00	0.46	0.96	0.67	0.79	15.10	11.13	4.78	101.73	27.80	3.61	72.75	13.67	5.67	0.80	0.49

M229	0.65	0.06	0.09	2.22	0.89	0.48	1.03	0.67	0.68	21.29	13.39	5.67	115.41	33.99	3.32	67.04	30.03	2.30	0.62	0.88
M230	0.38	0.21	0.51	2.78	1.11	0.37	1.13	0.74	0.55	14.59	5.19	5.89	110.83	64.01	2.15	76.84	22.23	3.92	0.73	0.37
M232	0.55	0.23	0.42	1.89	0.67	0.30	1.87	0.68	0.31	12.88	8.64	7.22	138.91	39.46	3.83	63.75	20.84	3.23	0.51	0.53
M233*	0.36	0.17	0.50	2.22	0.67	0.22	1.03	0.49	0.53	15.06	8.27	4.78	90.38	34.22	2.64	81.16	28.02	3.01	0.94	0.85
M234*	0.29	0.05	0.19	2.33	0.78	0.33	1.41	0.80	0.55	11.51	8.80	7.22	158.50	43.89	3.83	63.10	25.18	2.59	0.40	0.61
M235	0.41	0.24	0.46	2.22	0.89	0.44	1.03	0.79	0.85	12.68	6.52	5.00	86.61	53.57	1.78	67.84	20.16	3.43	0.80	0.40
M236	0.39	0.13	0.48	2.89	1.89	0.67	1.60	0.93	0.59	20.14	11.23	6.11	136.59	43.85	3.24	70.15	29.44	2.79	0.53	0.64
M237*	0.27	0.21	0.71	2.56	1.11	0.40	1.35	0.81	0.56	12.84	8.87	6.11	146.45	54.37	3.15	65.95	25.84	2.99	0.51	0.47
M238	0.29	0.14	0.63	2.72	1.11	0.46	1.18	0.61	0.47	11.91	8.97	5.67	134.19	31.56	4.23	63.66	29.52	2.19	0.48	0.94
M239	0.45	0.30	0.62	2.44	1.78	0.72	1.46	0.93	0.68	12.44	6.24	4.33	101.67	50.65	2.37	60.02	19.75	4.04	0.60	0.37
M240	0.42	0.23	0.50	2.33	0.89	0.41	1.16	0.62	0.63	11.15	10.24	6.00	120.39	38.91	3.03	67.64	25.30	2.70	0.60	0.66
M241	0.22	0.06	0.36	2.22	0.67	0.32	1.17	0.45	0.42	11.89	8.31	6.33	148.66	57.08	2.81	70.26	22.34	3.75	0.50	0.39
M242	0.18	0.09	0.74	1.22	0.56	0.67	0.63	0.54	0.84	11.66	6.44	6.33	143.44	43.33	3.32	81.59	26.06	3.13	0.69	0.60
M243*	0.24	0.06	0.28	1.89	1.00	0.53	1.01	0.67	0.65	10.35	9.50	6.56	138.17	58.09	3.20	61.75	19.12	3.50	0.47	0.36
M245	0.55	0.14	0.29	2.56	1.11	0.48	0.84	0.41	0.59	13.23	12.36	5.44	98.65	37.10	2.68	67.53	27.79	3.01	0.71	0.77
M246	0.25	0.11	0.37	2.33	0.56	0.23	0.80	0.69	0.90	9.41	8.73	7.44	125.84	55.87	2.45	70.41	20.87	3.97	0.56	0.35
M247*	0.36	0.28	0.79	2.67	1.33	0.53	1.51	1.02	0.79	25.86	15.65	6.56	154.10	60.28	2.78	71.06	36.67	2.14	0.47	0.62
M248*	0.23	0.06	0.64	2.44	1.44	0.57	1.02	0.81	0.74	11.54	3.87	6.56	125.49	62.31	2.10	74.95	21.95	3.68	0.67	0.36
M250	0.37	0.21	0.53	2.78	1.17	0.40	1.53	0.89	0.59	15.40	11.99	7.44	180.23	46.91	4.67	65.78	34.07	1.93	0.39	0.89
M251*	0.15	0.07	0.55	2.44	0.67	0.27	0.81	0.45	0.62	12.98	7.95	7.22	127.64	67.52	2.03	70.12	30.82	2.33	0.55	0.48
M252*	0.61	0.19	0.31	2.56	0.67	0.25	1.26	0.53	0.46	12.74	13.85	7.22	115.53	36.30	3.64	67.52	25.43	2.90	0.59	0.71
M253	0.23	0.21	0.80	1.89	1.00	0.47	1.05	0.63	0.61	11.52	6.29	5.89	95.97	46.91	2.51	75.21	14.61	5.52	0.94	0.33
M254	0.69	0.17	0.26	2.33	1.00	0.51	0.74	0.74	0.87	13.82	7.89	7.67	157.48	53.37	3.35	70.86	33.53	2.39	0.45	0.65
M255	0.50	0.15	0.28	2.00	1.00	0.36	1.49	0.82	0.64	10.85	10.59	6.78	139.68	48.32	3.43	55.72	20.71	3.04	0.42	0.45
M256	0.32	0.11	0.48	2.28	1.33	0.62	1.01	0.54	0.61	17.35	13.91	5.56	111.72	30.83	4.01	66.49	33.96	1.96	0.60	1.21
M258*	0.49	0.05	0.13	1.78	1.11	0.64	0.67	0.66	0.99	11.47	5.41	7.00	151.22	45.70	3.42	80.28	34.10	2.43	0.56	0.74
M260*	0.36	0.24	0.68	2.11	0.78	0.37	1.66	0.49	0.25	15.91	11.25	6.56	115.05	40.28	3.18	84.24	38.14	2.20	0.74	1.06
M261	0.57	0.29	0.71	2.00	0.89	0.39	1.65	0.67	0.40	14.30	8.89	7.44	152.18	44.98	4.41	63.99	28.03	2.45	0.44	0.72
M262	0.59	0.27	0.59	2.89	1.22	0.44	1.78	1.00	0.54	23.57	17.75	6.78	98.83	32.54	3.09	63.83	23.56	2.87	0.66	0.72
M263	0.29	0.07	0.35	1.89	0.56	0.25	1.19	0.48	0.64	16.09	17.19	7.44	152.47	42.37	3.66	53.70	26.35	2.02	0.35	0.65
M264	0.32	0.16	0.49	2.44	1.11	0.44	1.32	0.84	0.62	26.00	18.35	5.67	111.12	34.67	3.20	59.00	29.68	2.03	0.54	0.87
M265	0.26	0.08	0.30	2.56	0.89	0.34	1.39	0.67	0.50	20.44	13.60	6.11	134.04	53.88	2.69	62.79	30.02	2.19	0.46	0.56
M266	0.16	0.12	0.70	2.00	0.89	0.48	1.44	0.73	0.56	21.65	13.15	6.11	123.89	42.41	3.11	61.59	37.38	1.68	0.50	0.89
M267	0.22	0.14	0.70	2.56	1.33	0.51	0.96	0.67	0.60	14.11	11.03	6.33	133.28	33.02	4.08	66.22	24.89	2.91	0.52	0.77
M268	0.19	0.09	0.47	2.28	0.11	0.07	0.85	0.34	0.55	13.00	9.14	7.00	148.05	55.86	2.93	76.64	28.26	3.11	0.52	0.50
M269	0.67	0.25	0.38	2.44	0.89	0.38	1.23	0.70	0.62	14.41	10.02	6.56	114.99	44.35	3.05	71.23	18.26	4.85	0.62	0.40
M270	0.38	0.19	0.37	2.44	0.78	0.30	1.20	0.42	0.32	17.47	14.35	7.22	134.20	56.50	2.48	61.84	37.90	1.82	0.48	0.63
M271	0.40	0.15	0.56	2.11	1.00	0.39	1.14	0.71	0.69	11.76	6.47	5.44	94.83	60.37	2.47	79.46	20.66	4.09	0.86	0.51
M272	0.41	0.15	0.38	2.22	1.11	0.49	1.20	0.54	0.45	13.20	10.53	7.00	135.94	41.54	3.56	62.63	27.96	2.49	0.53	0.68
M274	0.43	0.20	0.48	2.67	1.44	0.57	1.29	0.72	0.60	23.53	22.62	5.89	130.00	52.26	2.58	70.16	31.89	2.39	0.54	0.59
M275	0.18	0.19	1.01	2.11	1.17	0.56	0.84	0.67	0.87	12.43	7.47	4.33	71.74	42.29	2.11	78.11	15.01	5.50	1.10	0.40
M276	0.51	0.19	0.67	3.00	1.44	0.48	1.40	0.85	0.61	20.27	16.63	5.67	114.35	28.89	4.03	69.64	16.73	4.24	0.61	0.59
M277	0.41	0.11	0.50	2.11	0.94	0.46	0.96	0.54	0.66	12.60	9.62	6.89	136.34	32.48	4.49	70.15	28.09	2.64	0.52	0.86

M278	0.46	0.25	0.55	2.00	0.78	0.41	0.93	0.51	0.58	21.64	13.30	5.67	138.27	47.59	3.23	79.44	36.26	2.30	0.58	0.84
M280	0.34	0.20	0.68	2.22	1.44	0.60	1.46	0.96	0.76	19.54	13.57	7.00	132.09	42.76	3.38	66.55	21.79	3.13	0.51	0.56
M281	0.36	0.20	0.52	2.11	1.00	0.47	1.05	0.65	0.60	14.27	9.78	7.44	151.96	37.66	4.20	74.26	35.21	2.25	0.50	0.93
M282	0.28	0.06	0.25	2.44	1.44	0.53	1.39	0.83	0.60	13.23	10.89	5.67	118.08	48.76	2.65	65.23	22.37	3.51	0.55	0.44
M283	0.58	0.15	0.22	1.89	1.44	0.75	1.44	0.90	0.78	18.34	12.83	6.56	164.93	47.84	3.61	73.07	32.43	2.25	0.46	0.73
M284	0.27	0.16	0.61	2.00	0.67	0.38	0.95	0.47	0.55	10.60	7.44	7.00	137.56	50.24	3.00	65.95	29.85	2.34	0.49	0.59
M285	0.14	0.05	0.42	2.44	1.11	0.48	1.21	0.78	0.75	25.07	17.38	6.56	121.23	42.79	3.25	73.49	26.33	2.80	0.61	0.67
M286	0.70	0.13	0.18	1.67	0.56	0.37	1.27	0.25	0.19	9.00	10.64	7.44	105.37	31.29	3.49	64.55	28.11	2.23	0.59	0.88
M287	0.57	0.21	0.37	2.06	1.22	0.55	1.39	1.07	0.74	17.75	8.96	7.22	158.87	38.64	4.23	66.75	28.25	2.38	0.43	0.73
M288*	0.72	0.18	0.45	2.78	0.72	0.27	1.66	0.56	0.31	18.46	9.74	6.67	137.35	29.43	4.61	88.21	28.63	3.21	0.72	1.01
M289	0.16	0.10	0.57	2.22	0.44	0.25	0.77	0.46	0.62	13.85	11.12	8.11	166.47	65.48	2.62	76.40	33.35	2.24	0.45	0.51
M290	0.59	0.23	0.47	2.00	0.56	0.28	1.08	0.46	0.39	16.49	12.66	7.00	129.35	35.05	3.76	74.17	26.99	2.90	0.58	0.76
M291	0.46	0.19	0.47	2.44	1.11	0.45	1.28	0.77	0.62	20.36	11.18	5.00	120.75	33.89	3.96	65.85	27.87	2.49	0.58	0.83
M292	0.54	0.10	0.19	2.00	0.44	0.25	1.25	0.44	0.37	11.54	13.79	7.00	137.87	53.16	3.53	68.13	29.59	2.59	0.52	0.62
M293	0.37	0.08	0.22	2.78	2.11	0.77	1.53	1.24	0.89	22.23	17.04	5.67	110.61	50.19	2.40	60.33	32.78	1.90	0.54	0.68
M294*	0.39	0.12	0.35	2.33	1.22	0.53	1.48	0.86	0.59	16.23	9.64	6.33	137.79	41.90	3.55	60.34	29.61	2.15	0.44	0.74
M295	0.35	0.12	0.22	2.44	1.11	0.45	1.29	0.65	0.45	13.99	9.77	7.67	149.92	60.30	2.76	64.70	27.71	2.88	0.45	0.48
M296	0.31	0.11	0.33	1.67	1.00	0.61	1.20	0.48	0.40	17.23	11.34	5.89	82.54	35.57	2.35	75.58	24.28	3.53	0.99	0.69
M297	0.10	0.09	1.09	1.56	0.78	0.52	0.68	0.56	0.86	11.50	10.70	5.89	96.86	38.43	2.53	69.57	17.41	3.98	0.74	0.46
M298	0.40	0.16	0.39	2.78	1.00	0.37	1.17	0.81	0.83	15.26	10.42	5.67	126.38	59.13	2.36	74.34	22.15	3.36	0.67	0.41
M299	0.33	0.12	0.38	2.56	1.22	0.47	0.97	0.60	0.67	12.58	7.25	6.33	117.70	38.27	3.25	61.61	28.84	2.17	0.53	0.81
M300*	0.65	0.25	0.38	2.00	1.67	0.87	1.38	1.24	1.03	23.28	12.97	5.44	130.72	61.59	2.88	59.82	28.27	2.27	0.46	0.55
M301	0.47	0.12	0.32	2.67	0.78	0.28	1.77	0.65	0.41	17.32	13.74	6.78	135.80	82.13	2.23	68.66	25.99	2.68	0.51	0.38
M303	0.29	0.13	0.43	1.89	0.89	0.65	0.88	0.45	0.58	21.00	13.79	6.11	105.89	42.12	2.90	75.30	29.28	2.91	0.81	0.67
M304	0.39	0.15	0.27	3.11	1.22	0.38	1.82	1.22	0.64	16.01	10.09	6.33	152.49	45.45	4.38	57.75	29.75	2.18	0.39	0.71
M305	0.40	0.12	0.26	2.67	1.33	0.50	1.49	0.82	0.57	19.99	13.10	7.22	163.44	52.86	4.02	60.48	26.76	2.48	0.41	0.58
M306	0.26	0.13	0.52	1.33	0.89	0.67	1.01	0.47	0.47	14.01	7.68	5.22	73.80	63.46	1.76	64.82	18.99	3.73	0.92	0.37
M307	0.52	0.36	0.93	2.67	0.78	0.28	1.27	0.54	0.60	13.70	7.41	6.33	124.06	42.45	2.95	74.94	29.51	2.97	0.61	0.82
M308*	0.41	0.20	0.54	2.33	1.78	0.77	0.90	0.71	0.83	12.67	5.21	5.00	97.79	37.25	2.63	64.97	28.89	2.40	0.67	0.81
M311*	0.43	0.14	0.33	2.78	1.67	0.61	1.80	1.09	0.67	25.92	16.75	5.89	124.68	43.69	3.10	57.44	34.61	1.75	0.48	0.84
M312	0.69	0.40	0.54	3.00	1.22	0.44	1.81	1.25	0.73	28.78	15.72	5.22	134.31	53.62	2.87	75.52	36.30	2.11	0.58	0.72
M315	0.34	0.21	0.51	2.78	1.56	0.59	1.82	1.26	0.65	25.42	21.02	5.89	142.21	41.28	3.63	66.65	29.28	2.53	0.50	0.72
M317*	0.49	0.22	0.55	1.67	0.78	0.51	1.06	0.42	0.45	13.59	13.30	8.56	133.70	44.63	2.96	83.47	25.67	3.43	0.66	0.57
M318*	0.30	0.13	0.47	1.89	0.78	0.46	1.08	0.47	0.62	12.86	10.26	7.89	155.50	41.09	4.56	74.86	26.32	2.85	0.48	0.77
M319*	0.39	0.06	0.28	2.44	1.78	0.68	1.20	0.62	0.52	16.67	14.30	7.00	140.29	39.29	3.84	74.24	21.20	3.93	0.53	0.53
M321	0.34	0.12	0.36	2.11	1.56	0.73	0.94	0.96	1.06	20.04	13.92	5.00	119.00	49.27	2.63	60.22	28.45	2.24	0.51	0.59
M322*	0.58	0.20	0.28	2.00	1.44	0.75	1.34	1.03	0.78	24.16	18.98	4.33	107.59	46.90	2.35	63.54	25.42	2.55	0.65	0.57
M324	0.29	0.18	0.53	1.72	0.72	0.42	0.89	0.55	0.64	9.06	5.85	5.67	104.23	53.63	2.10	51.26	32.98	1.61	0.51	0.64
M325	0.27	0.12	0.59	2.44	1.00	0.42	1.11	0.50	0.47	12.68	10.11	8.33	131.05	42.00	3.40	76.72	24.74	3.11	0.73	0.60
M326	0.35	0.10	0.32	2.33	0.78	0.34	0.95	0.40	0.44	13.49	9.78	5.89	175.54	49.16	3.52	69.91	30.94	2.53	0.44	0.64
M328*	0.14	0.08	0.66	2.67	1.33	0.50	1.24	0.71	0.58	21.34	15.75	5.67	118.17	34.93	3.44	67.41	24.00	2.83	0.61	0.69
M329	0.37	0.22	0.61	3.00	1.11	0.29	1.20	0.98	0.67	17.06	10.95	6.56	138.95	46.32	4.10	71.12	32.83	2.39	0.55	0.79
M330*	0.20	0.10	0.50	1.89	0.89	0.56	0.84	0.49	0.78	11.98	9.76	6.78	110.53	37.41	3.29	65.28	23.97	3.32	0.61	0.63

M331	0.49	0.18	0.36	2.00	1.11	0.57	1.90	0.74	0.42	20.46	13.81	7.44	140.57	45.02	3.20	72.04	31.74	2.39	0.52	0.71
M332	0.38	0.12	0.28	2.67	1.17	0.47	1.16	0.65	0.72	14.01	9.36	5.89	113.17	35.01	3.45	65.64	15.78	4.61	0.58	0.45
M333	0.40	0.15	0.43	2.00	1.22	0.58	1.69	0.96	0.55	20.26	9.57	5.67	143.88	42.72	3.43	71.82	25.82	2.78	0.50	0.61
M334	0.31	0.12	0.53	2.56	1.00	0.42	1.25	0.71	0.55	13.81	8.06	7.44	163.68	50.48	3.31	84.03	34.64	2.45	0.51	0.69
M336	0.18	0.02	0.16	3.11	0.67	0.23	1.39	0.53	0.42	7.10	8.88	7.67	161.12	50.87	3.56	67.82	22.03	3.11	0.42	0.46
M337	0.61	0.27	0.36	2.00	0.78	0.39	1.54	1.05	0.46	19.27	11.02	7.22	144.31	47.25	3.48	67.43	28.41	2.68	0.49	0.59
M338*	0.26	0.19	0.49	2.56	1.33	0.51	1.09	0.76	0.62	18.59	14.81	6.33	136.40	23.97	5.68	67.37	21.59	3.14	0.52	0.95
M339	0.47	0.18	0.48	2.89	1.33	0.46	1.24	0.62	0.53	20.78	19.14	6.78	122.14	36.57	3.76	68.22	31.03	2.40	0.56	0.91
M341	0.34	0.09	0.14	2.67	0.78	0.29	1.41	0.57	0.42	20.25	13.39	7.89	124.14	32.98	3.98	66.18	23.80	2.88	0.55	0.72
M342	0.19	0.08	0.48	1.89	0.89	0.48	0.86	0.60	0.76	11.04	8.50	7.00	109.38	32.15	3.40	59.74	38.94	1.60	0.57	1.22
M343	0.49	0.37	0.80	2.33	1.44	0.59	1.98	1.30	0.63	23.99	15.97	5.67	155.85	48.29	3.40	64.66	22.96	2.82	0.45	0.52
M344	0.31	0.20	0.69	1.44	0.89	0.60	0.86	0.52	0.71	16.74	13.03	6.78	128.93	31.26	4.13	76.84	29.46	2.63	0.61	0.96
M345	0.88	0.59	0.65	2.56	1.22	0.44	1.87	1.53	0.75	20.63	13.00	5.44	106.28	44.20	2.40	66.33	25.81	2.56	0.62	0.58
M346	0.45	0.22	0.52	1.56	1.22	0.70	0.84	0.78	0.80	20.65	20.26	6.78	116.51	36.56	3.28	65.31	21.42	3.31	0.56	0.60
M347	0.38	0.01	0.03	2.78	0.67	0.30	1.34	0.61	0.62	12.06	13.33	7.67	167.89	50.50	3.52	81.91	23.24	3.48	0.49	0.47
M348	0.31	0.19	0.43	2.11	1.11	0.56	1.23	0.98	0.68	17.47	13.33	6.33	117.41	44.88	2.94	60.85	38.71	1.83	0.53	0.84
M349	0.48	0.27	0.67	2.78	1.33	0.48	1.06	0.63	0.60	18.22	9.37	6.56	174.62	63.48	3.00	73.91	39.50	1.98	0.42	0.63
M351	0.64	0.34	0.43	2.44	1.33	0.55	1.75	0.80	0.46	25.23	16.60	6.56	122.93	33.11	3.81	70.22	24.41	3.35	0.57	0.74
M352	0.26	0.10	0.36	2.00	0.67	0.33	1.06	0.46	0.40	12.41	7.98	7.00	118.62	37.31	3.41	69.35	27.51	2.79	0.63	0.73
M353*	0.32	0.14	0.65	2.22	1.00	0.49	1.17	0.58	0.55	14.70	11.32	6.33	118.28	29.93	3.94	69.62	29.94	2.44	0.62	1.07
M355	0.30	0.16	0.62	1.78	0.89	0.39	1.14	0.55	0.52	13.24	10.85	6.78	153.94	53.24	3.77	62.12	31.32	2.36	0.44	0.60
M356	0.41	0.25	0.63	2.00	1.22	0.71	1.39	0.94	0.72	19.40	11.95	7.22	115.37	40.02	2.98	71.35	37.43	2.13	0.62	0.92
M357	0.59	0.11	0.16	2.33	1.06	0.45	1.41	0.51	0.34	19.48	14.55	7.67	144.14	45.77	3.58	85.87	23.95	4.06	0.59	0.52
M358*	0.30	0.16	0.43	2.22	0.89	0.47	1.14	0.66	0.51	16.24	7.37	6.33	173.98	58.62	2.91	83.72	35.31	2.53	0.50	0.61
M359	0.58	0.14	0.43	2.33	1.44	0.58	1.38	0.88	0.62	26.46	14.90	5.00	96.94	47.82	2.12	61.65	34.92	1.81	0.64	0.74
M360	0.66	0.16	0.29	2.78	0.56	0.19	1.83	0.28	0.23	16.70	13.20	8.33	137.56	41.74	3.56	76.29	36.77	2.07	0.56	0.91
M361	0.31	0.17	0.58	3.00	1.44	0.47	1.03	0.67	0.67	21.45	12.33	5.44	93.24	33.96	2.86	70.21	20.75	3.75	0.77	0.64
M362*	0.21	0.14	1.21	2.22	1.11	0.62	1.03	0.68	0.89	17.15	14.05	6.33	105.11	34.18	3.09	71.63	32.02	2.25	0.68	0.94
M363	0.42	0.11	0.62	2.00	0.78	0.38	0.97	0.73	0.80	16.46	11.50	5.89	108.09	40.11	2.79	67.30	27.13	2.84	0.64	0.73
M364	0.28	0.11	0.41	1.44	1.11	0.77	0.76	0.62	0.84	18.33	18.08	5.67	87.10	34.74	2.59	70.90	25.47	2.98	0.82	0.72
M367	0.78	0.09	0.27	2.22	1.00	0.40	1.30	0.52	0.53	14.59	19.76	7.22	124.56	33.38	3.73	68.01	21.28	3.31	0.56	0.64
M371	0.56	0.44	0.75	2.33	1.33	0.57	1.57	1.03	0.85	22.18	13.84	5.67	115.03	29.90	4.00	70.55	26.09	2.77	0.69	0.86
M372	0.37	0.13	0.23	2.78	1.78	0.67	1.15	0.86	0.82	22.96	19.12	4.78	109.29	44.99	2.46	59.49	24.41	2.48	0.54	0.55
M373*	0.53	0.21	0.27	2.56	0.89	0.39	1.08	0.77	0.73	17.53	9.14	5.89	91.73	40.89	2.43	54.29	19.39	2.94	0.59	0.48
M374	0.28	0.13	0.81	1.39	0.56	0.46	0.79	0.38	0.56	12.37	8.88	6.33	93.14	32.71	2.88	59.12	30.04	2.24	0.64	0.93
M375	0.39	0.08	0.21	2.44	0.78	0.31	1.52	0.54	0.42	14.66	14.42	7.22	148.99	31.32	4.79	77.78	30.25	2.68	0.52	0.95
M376	0.28	0.17	0.60	2.22	1.56	0.73	1.06	0.68	0.69	20.58	13.71	6.78	128.35	33.03	3.98	62.30	29.59	2.16	0.54	0.89
M377	0.48	0.29	0.88	2.83	1.11	0.44	0.97	0.56	0.59	23.42	13.39	7.00	108.55	35.28	3.13	79.81	20.45	4.16	0.74	0.57
M378	0.27	0.26	0.87	3.00	1.11	0.36	1.61	1.25	0.76	22.27	16.92	6.78	144.76	48.07	3.06	77.09	37.25	2.07	0.53	0.79
M379*	0.51	0.30	0.60	2.11	0.89	0.45	1.38	0.86	0.65	17.94	10.84	4.11	99.66	54.16	2.04	80.35	37.16	2.30	0.87	0.72
M380	0.46	0.26	0.52	2.11	0.67	0.38	1.27	0.62	0.50	12.78	7.06	5.89	142.42	60.22	2.91	69.60	26.77	2.73	0.57	0.47
M381	0.35	0.17	0.52	2.33	1.33	0.56	1.42	0.72	0.52	15.01	8.47	5.67	133.71	47.46	3.14	74.65	31.62	2.45	0.60	0.68
M383	0.27	0.05	0.39	2.33	0.67	0.31	1.52	0.63	0.44	21.12	16.10	6.33	156.07	48.73	3.52	70.00	25.36	2.92	0.44	0.57

M384	0.31	0.22	0.85	1.89	0.89	0.50	0.74	0.41	0.53	9.36	4.02	6.33	117.01	48.47	2.95	81.23	24.51	3.39	0.69	0.58
M385	0.24	0.08	0.96	2.89	0.89	0.31	1.03	0.42	0.40	19.90	11.52	5.67	123.95	48.18	2.70	61.79	35.55	1.77	0.50	0.77
M386	0.47	0.17	0.40	1.89	0.67	0.36	1.04	0.40	0.41	11.62	9.34	7.22	129.23	40.13	3.26	57.27	22.96	2.50	0.45	0.58
M389	0.47	0.13	0.28	2.17	1.11	0.52	1.17	0.45	0.39	17.83	12.33	5.67	128.62	41.38	3.54	71.30	28.88	2.61	0.55	0.82
M392	0.50	0.09	0.20	2.67	0.89	0.29	1.69	0.52	0.31	23.13	11.65	5.00	100.40	37.35	2.87	62.09	23.54	2.65	0.63	0.63
M393*	0.25	0.20	1.77	2.67	1.00	0.35	1.30	0.97	0.68	20.63	14.45	5.67	115.11	59.52	2.07	64.23	34.84	1.68	0.55	0.58
M394	0.32	0.16	0.82	2.00	1.11	0.61	1.11	0.55	0.48	11.77	6.86	5.22	86.74	35.41	2.50	84.58	22.84	4.11	1.06	0.64
M397	0.31	0.27	0.81	1.56	0.00	0.00	0.56	0.38	0.67	10.67	6.02	6.33	135.64	42.56	3.17	68.39	23.42	2.93	0.51	0.55
M398	0.44	0.20	0.48	2.33	1.00	0.45	0.93	0.42	0.47	13.22	10.26	7.00	120.98	36.26	3.39	76.27	30.53	2.50	0.66	0.89
M399*	0.28	0.16	0.48	1.89	1.00	0.59	0.70	0.73	1.09	13.90	9.64	4.78	77.30	42.90	1.80	78.78	17.02	5.26	1.19	0.39
M400	0.47	0.19	0.39	2.56	1.06	0.40	0.84	0.72	0.90	18.55	11.15	7.89	138.80	33.87	4.11	75.78	24.19	3.28	0.57	0.73
M401*	0.55	0.31	0.57	2.11	1.22	0.57	1.80	0.60	0.31	26.89	18.89	6.33	132.34	50.03	3.17	72.76	28.30	2.95	0.60	0.63
M402*	0.52	0.24	0.49	2.06	1.22	0.50	0.84	0.41	0.57	8.82	6.97	6.33	155.13	30.95	4.98	73.50	17.98	4.08	0.49	0.58
M403	0.39	0.18	0.56	2.44	0.89	0.40	1.00	0.51	0.54	9.14	9.94	6.56	157.08	42.84	3.94	68.18	23.53	3.03	0.47	0.57
M405*	0.23	0.17	0.74	2.17	0.89	0.47	0.57	0.50	0.86	8.83	5.70	6.56	137.64	30.88	4.55	64.90	37.01	1.83	0.47	1.22
M406	0.71	0.07	0.11	3.11	1.78	0.57	2.44	0.98	0.40	24.21	14.92	5.89	122.72	37.59	3.24	64.90	28.31	2.44	0.56	0.75
M407	0.42	0.14	0.38	2.44	0.89	0.36	0.86	0.54	0.62	20.40	14.61	6.78	111.64	35.54	3.21	72.55	28.06	2.70	0.65	0.79
M410	0.49	0.17	0.55	3.00	0.50	0.18	1.28	0.54	0.42	9.51	12.91	8.56	160.04	38.39	4.08	77.96	26.27	3.03	0.53	0.72
M411*	0.34	0.01	0.03	3.00	0.89	0.27	0.90	0.43	0.47	11.37	8.43	7.00	177.62	33.95	5.42	80.32	29.26	2.82	0.45	0.86
Mxg	0.66	0.10	0.18	1.78	1.00	0.54	1.36	0.48	0.37	11.24	8.18	6.11	125.97	53.96	2.68	60.54	27.38	2.21	0.52	0.59

*64 genotypes of mini-core collection developed using the Core Hunter algorithm.

GR_CK: Shoot growth rate under control, GR_S: Shoot growth rate under 150 mM NaCl, RGR: salt-tolerance index of shoot growth rate, NIL_CK: leaves increased number under control, NIL_S: leaves increased number under 150 mM NaCl, RNIL: salt-tolerance index of leaves increased number, LER_CK: leaf expansion rate under control, LER_S: leaf expansion rate under 150 mM NaCl, RLER: salt-tolerance index of leaf expansion rate, Sen: leaf senescence scale, SWC: shoot water content, RWC: root water content, SNC: shoot Na^+ concentration, RNC: root Na^+ concentration, SN/RN: the ratio of shoot Na^+ concentration to root Na^+ concentration, SKC: shoot K^+ concentration, RKC: root K^+ concentration, SK/RK: the ratio of shoot K^+ concentration to root K^+ concentration, SK/N: the ratio of shoot K^+ concentration to shoot Na^+ concentration, RK/N: the ratio of root K^+ concentration to root Na^+ concentration.

Sen, SWC, RWC, SNC, RNC, SN/RN, SKC, RKC, SK/RK, SK/N and RK/N were evaluated under 150 mM NaCl treatment.

Table S5. MFVs and salt tolerance comprehensive evaluation of 318 *M. sacchariflorus* and *M. lutarioriparius* genotypes at the seedling stage.

Genotype	MFV of RGR	MFV of RNIL	MFV of RLER	MFV of SWC	MFV of RWC	MFV of Sen	MFV of SNC	MFV of RNC	MFV of SN/RN	MFV of SKC	MFV of RKC	MFV of SK/RK	MFV of SK/N	MFV of RK/N	D value	Category
M135*	0.74	0.71	0.51	0.64	0.37	0.91	0.91	0.80	0.82	0.54	0.66	0.42	0.75	0.36	0.65	HST
M399*	0.25	0.62	1.00	0.39	0.32	0.78	0.86	0.67	0.86	0.43	0.84	0.83	0.86	0.13	0.63	HST
M275	0.56	0.59	0.76	0.33	0.20	0.87	0.89	0.69	0.79	0.44	0.92	0.88	0.77	0.14	0.63	HST
M228*	0.42	0.49	0.66	0.44	0.40	0.78	0.71	0.93	0.46	0.53	0.97	0.92	0.47	0.24	0.60	ST
M29*	0.57	0.83	0.76	0.51	0.21	1.00	0.86	0.43	0.96	0.62	0.57	0.32	0.58	0.16	0.60	ST
M364	0.22	0.81	0.72	0.57	0.76	0.61	0.80	0.81	0.68	0.56	0.53	0.31	0.50	0.48	0.60	ST
M205*	0.36	1.00	0.83	0.77	0.76	0.52	0.67	0.85	0.52	0.72	0.24	0.06	0.25	0.79	0.60	ST
M225	0.31	0.84	0.60	0.82	0.84	1.00	0.73	0.47	0.79	0.84	0.48	0.13	0.22	0.26	0.59	ST
M124	0.16	0.82	0.48	0.79	0.92	0.52	0.73	0.91	0.51	0.77	0.49	0.17	0.27	0.72	0.59	ST
M322*	0.14	0.79	0.65	0.81	0.81	0.87	0.67	0.61	0.74	0.68	0.53	0.21	0.32	0.32	0.58	ST
M60	0.24	0.71	0.71	0.54	0.43	0.65	0.85	0.81	0.76	0.59	0.41	0.20	0.58	0.64	0.58	ST
M361	0.32	0.50	0.54	0.70	0.46	0.65	0.76	0.83	0.63	0.57	0.70	0.48	0.44	0.39	0.57	ST
M372	0.11	0.71	0.70	0.76	0.82	0.78	0.66	0.64	0.72	0.75	0.57	0.20	0.22	0.30	0.57	ST
M209	0.70	0.64	0.66	0.62	0.47	0.57	0.70	0.88	0.47	0.73	0.37	0.14	0.26	0.72	0.57	ST
M297	0.61	0.55	0.74	0.29	0.37	0.57	0.74	0.75	0.70	0.58	0.83	0.54	0.42	0.21	0.56	ST
M346	0.28	0.74	0.68	0.67	0.88	0.39	0.62	0.78	0.53	0.65	0.68	0.39	0.23	0.36	0.56	ST
M45*	0.10	0.48	0.23	0.90	0.46	0.65	0.87	0.72	0.86	0.41	0.52	0.36	0.91	0.39	0.56	ST
M371	0.41	0.61	0.74	0.73	0.54	0.61	0.63	0.90	0.37	0.57	0.50	0.26	0.36	0.63	0.56	ST
M276	0.36	0.50	0.46	0.65	0.69	0.61	0.63	0.92	0.37	0.58	0.85	0.59	0.28	0.34	0.56	ST
M174	0.71	0.46	0.67	0.33	0.19	0.78	0.81	0.67	0.70	0.57	0.63	0.43	0.56	0.32	0.56	ST
M220	0.21	1.00	0.65	0.74	0.76	0.48	0.58	0.81	0.47	0.62	0.48	0.23	0.23	0.54	0.56	ST
M134	0.39	0.75	0.85	0.88	0.62	0.70	0.58	0.65	0.55	0.78	0.34	0.07	0.13	0.51	0.56	ST
M362*	0.67	0.66	0.78	0.52	0.55	0.48	0.69	0.82	0.57	0.55	0.28	0.15	0.36	0.71	0.56	ST
M24	0.41	0.55	0.40	0.51	0.35	0.78	0.79	0.67	0.78	0.66	0.76	0.53	0.41	0.17	0.56	ST
M179	0.47	0.68	0.69	0.50	0.23	0.74	0.85	0.34	0.96	0.49	0.44	0.40	0.78	0.18	0.55	ST
M113	0.19	0.75	0.34	0.41	0.22	0.74	0.75	0.62	0.80	0.40	0.94	1.00	0.52	0.05	0.55	ST
M34	0.19	0.55	0.44	0.79	0.55	0.65	0.72	0.80	0.62	0.63	0.66	0.36	0.35	0.41	0.55	ST
M136	0.50	0.58	0.77	0.57	0.29	0.96	0.74	0.16	0.94	0.77	0.73	0.32	0.35	0.04	0.55	ST
M148	0.28	0.75	0.57	0.42	0.19	0.78	0.79	0.55	0.74	0.45	0.66	0.73	0.58	0.22	0.55	ST
M199	0.40	0.74	0.54	0.73	0.80	0.61	0.53	0.86	0.36	0.57	0.46	0.24	0.25	0.62	0.55	ST
M192*	0.05	0.73	0.53	0.17	0.14	0.78	1.00	0.58	1.00	0.72	0.43	0.27	1.00	0.30	0.55	ST
M130	0.16	0.56	0.77	0.60	0.55	0.48	0.66	0.89	0.46	0.73	0.77	0.43	0.25	0.36	0.55	ST
M139*	0.30	0.79	0.49	0.50	0.18	0.91	0.72	0.32	0.89	0.53	0.82	0.72	0.47	0.01	0.55	ST
M118	0.34	0.68	0.37	0.64	0.45	0.67	0.68	0.75	0.66	0.60	0.65	0.33	0.45	0.35	0.55	ST
M86	0.65	0.48	0.43	0.53	0.72	0.57	0.68	0.80	0.56	0.74	0.60	0.24	0.24	0.41	0.55	ST
M90	0.43	0.49	0.69	0.34	0.49	0.74	0.73	0.47	0.83	0.83	0.79	0.35	0.34	0.10	0.54	ST
M262	0.32	0.47	0.38	0.79	0.74	0.39	0.73	0.85	0.57	0.68	0.60	0.29	0.34	0.47	0.54	ST
M132	0.63	0.72	0.62	0.72	0.83	0.70	0.50	0.69	0.49	0.81	0.19	0.04	0.08	0.60	0.54	ST
M177*	0.61	0.52	0.65	0.31	0.51	0.57	0.70	0.94	0.43	0.94	0.40	0.03	0.14	0.84	0.54	ST
M215	0.37	0.38	0.15	0.77	0.67	0.61	0.74	0.87	0.57	0.83	0.74	0.26	0.23	0.39	0.54	ST

M264	0.27	0.46	0.48	0.89	0.78	0.61	0.65	0.82	0.55	0.76	0.37	0.10	0.21	0.63	0.54	ST
M3	0.24	0.65	0.57	0.53	0.25	0.65	0.79	0.73	0.72	0.76	0.71	0.30	0.36	0.28	0.54	ST
M394	0.45	0.65	0.32	0.30	0.17	0.70	0.80	0.80	0.71	0.33	0.62	0.57	0.73	0.39	0.54	ST
M43	0.35	0.61	0.85	0.37	0.30	0.70	0.80	0.33	0.92	0.52	0.66	0.48	0.56	0.10	0.54	ST
M42	0.40	0.66	0.52	0.36	0.39	0.70	0.71	0.47	0.83	0.48	0.81	0.73	0.42	0.05	0.54	ST
M377	0.49	0.46	0.44	0.78	0.52	0.35	0.67	0.81	0.57	0.41	0.71	0.58	0.42	0.32	0.54	ST
M321	0.19	0.77	0.96	0.64	0.54	0.74	0.61	0.57	0.68	0.74	0.41	0.15	0.18	0.34	0.54	ST
M296	0.17	0.65	0.23	0.53	0.41	0.57	0.82	0.80	0.74	0.48	0.57	0.43	0.66	0.44	0.54	ST
M31	0.30	0.93	0.78	0.42	0.22	0.91	0.81	0.14	1.00	0.51	0.51	0.30	0.55	0.10	0.53	ST
M116*	0.88	0.51	0.49	0.40	0.26	0.48	0.77	0.79	0.69	0.84	0.44	0.08	0.30	0.53	0.53	ST
M253	0.44	0.50	0.46	0.29	0.14	0.57	0.74	0.61	0.70	0.49	0.93	0.88	0.61	0.07	0.53	ST
M115	0.35	0.80	0.71	0.26	0.12	0.72	0.76	0.74	0.71	0.67	0.53	0.22	0.38	0.48	0.53	ST
M183	0.45	0.43	0.75	0.66	0.78	0.70	0.58	0.48	0.73	0.70	0.53	0.22	0.20	0.23	0.53	ST
M359	0.23	0.61	0.48	0.90	0.59	0.74	0.74	0.59	0.79	0.71	0.17	0.05	0.31	0.50	0.53	MST
M293	0.11	0.81	0.78	0.73	0.71	0.61	0.66	0.55	0.73	0.74	0.25	0.07	0.21	0.44	0.53	MST
M328*	0.36	0.53	0.43	0.69	0.64	0.61	0.61	0.81	0.50	0.62	0.58	0.28	0.28	0.44	0.53	MST
M37*	0.38	0.43	0.63	0.70	0.38	0.78	0.80	0.25	0.92	0.52	0.57	0.40	0.52	0.11	0.53	MST
M239	0.34	0.76	0.55	0.33	0.14	0.87	0.71	0.54	0.73	0.74	0.74	0.55	0.27	0.11	0.53	MST
M1	0.27	0.76	0.41	0.80	0.45	0.61	0.59	0.82	0.47	0.49	0.60	0.36	0.29	0.45	0.53	MST
M193	0.40	0.40	0.31	0.53	0.71	0.48	0.70	0.85	0.56	0.67	0.67	0.31	0.33	0.41	0.52	MST
M128	0.55	0.57	0.44	0.62	0.38	0.78	0.80	0.51	0.88	0.78	0.23	0.04	0.35	0.41	0.52	MST
M308*	0.29	0.81	0.71	0.34	0.09	0.74	0.73	0.77	0.68	0.66	0.40	0.18	0.34	0.58	0.52	MST
M149*	0.24	0.66	0.71	0.46	0.47	0.74	0.65	0.43	0.76	0.70	0.68	0.44	0.25	0.12	0.52	MST
M345	0.36	0.46	0.63	0.67	0.49	0.65	0.68	0.65	0.73	0.64	0.51	0.22	0.29	0.33	0.52	MST
M28	0.55	0.48	0.59	0.52	0.28	0.89	0.84	0.31	0.92	0.64	0.31	0.13	0.52	0.28	0.52	MST
M121	0.32	0.56	0.34	0.40	0.44	0.61	0.77	0.91	0.51	0.80	0.45	0.15	0.29	0.73	0.52	MST
M306	0.28	0.71	0.31	0.39	0.22	0.70	0.88	0.32	0.87	0.66	0.77	0.48	0.59	0.11	0.52	MST
M125	0.21	0.67	0.45	0.43	0.41	0.61	0.69	0.84	0.53	0.61	0.59	0.34	0.41	0.48	0.52	MST
M315	0.27	0.62	0.51	0.86	0.92	0.57	0.47	0.70	0.46	0.63	0.38	0.21	0.17	0.48	0.52	MST
M300*	0.20	0.92	0.94	0.77	0.49	0.65	0.54	0.35	0.62	0.74	0.42	0.15	0.14	0.30	0.52	MST
M117	0.39	0.74	0.70	0.44	0.09	0.61	0.70	0.69	0.69	0.82	0.66	0.23	0.20	0.27	0.52	MST
M235	0.24	0.47	0.74	0.34	0.15	0.74	0.80	0.49	0.87	0.61	0.73	0.41	0.47	0.14	0.51	MST
M351	0.23	0.58	0.30	0.85	0.68	0.43	0.58	0.84	0.41	0.57	0.57	0.40	0.25	0.50	0.51	MST
M22	0.28	0.36	0.52	0.51	0.55	0.65	0.80	0.71	0.75	0.54	0.27	0.15	0.50	0.61	0.51	MST
M256	0.26	0.66	0.46	0.53	0.54	0.63	0.65	0.88	0.37	0.63	0.21	0.08	0.27	0.99	0.51	MST
M67	0.41	0.55	0.83	0.40	0.32	0.70	0.70	0.42	0.79	0.54	0.53	0.43	0.37	0.16	0.51	MST
M285	0.22	0.51	0.62	0.85	0.73	0.43	0.59	0.68	0.54	0.52	0.49	0.27	0.28	0.42	0.51	MST
M303	0.23	0.69	0.44	0.68	0.54	0.52	0.68	0.69	0.62	0.49	0.38	0.30	0.48	0.42	0.51	MST
M165*	0.38	0.55	0.47	0.38	0.30	0.52	0.63	0.65	0.69	0.39	0.82	0.75	0.43	0.19	0.51	MST
M50	0.33	0.61	0.71	0.55	0.39	0.63	0.51	0.59	0.60	0.43	0.77	0.58	0.29	0.14	0.51	MST
M14*	0.40	0.65	0.80	0.47	0.40	0.43	0.54	0.90	0.24	0.69	0.62	0.28	0.16	0.56	0.51	MST
M173	0.27	0.61	0.26	0.36	0.22	0.57	0.80	0.93	0.57	0.77	0.58	0.21	0.33	0.66	0.51	MST
M129	0.15	0.38	0.28	0.47	0.41	0.48	0.78	0.96	0.50	0.44	0.52	0.43	0.55	0.76	0.51	MST

M373*	0.14	0.41	0.60	0.54	0.29	0.57	0.77	0.71	0.72	0.84	0.75	0.30	0.26	0.22	0.51	MST
M226	0.40	0.56	0.81	0.27	0.24	0.48	0.70	0.73	0.57	0.78	0.70	0.39	0.24	0.25	0.51	MST
M153*	0.40	0.80	0.85	0.30	0.13	0.61	0.47	0.52	0.63	0.59	0.86	0.73	0.17	0.05	0.51	MST
M171	0.30	0.46	0.61	0.44	0.22	0.52	0.64	0.82	0.51	0.54	0.85	0.62	0.31	0.24	0.51	MST
M221	0.24	0.52	0.55	0.63	0.58	0.70	0.60	0.68	0.55	0.57	0.46	0.28	0.26	0.48	0.51	MST
M363	0.34	0.40	0.68	0.49	0.42	0.57	0.67	0.72	0.64	0.62	0.46	0.28	0.31	0.49	0.51	MST
M332	0.14	0.49	0.59	0.39	0.30	0.57	0.64	0.81	0.50	0.65	0.89	0.68	0.25	0.19	0.51	MST
M376	0.33	0.78	0.55	0.66	0.53	0.39	0.55	0.84	0.38	0.70	0.37	0.13	0.21	0.66	0.51	MST
M145	0.22	0.68	0.66	0.40	0.23	0.70	0.69	0.57	0.79	0.47	0.64	0.40	0.42	0.20	0.51	MST
M311*	0.17	0.64	0.53	0.88	0.69	0.57	0.57	0.66	0.57	0.78	0.18	0.03	0.16	0.60	0.50	MST
M143	0.33	0.66	0.72	0.50	0.14	0.57	0.68	0.41	0.81	0.63	0.71	0.50	0.30	0.09	0.50	MST
M112	0.35	0.59	0.60	0.58	0.32	0.61	0.62	0.70	0.58	0.63	0.48	0.22	0.29	0.47	0.50	MST
M343	0.44	0.62	0.49	0.80	0.65	0.61	0.39	0.58	0.51	0.66	0.62	0.27	0.13	0.27	0.50	MST
M245	0.15	0.50	0.44	0.36	0.46	0.65	0.73	0.77	0.66	0.62	0.44	0.32	0.38	0.53	0.50	MST
M274	0.26	0.61	0.46	0.78	1.00	0.57	0.54	0.51	0.69	0.57	0.29	0.18	0.21	0.35	0.50	MST
M393*	1.00	0.37	0.54	0.67	0.57	0.61	0.63	0.39	0.80	0.67	0.17	0.02	0.23	0.33	0.50	MST
M110	0.36	0.41	0.54	0.63	0.41	0.57	0.56	0.96	0.19	0.55	0.46	0.26	0.28	0.84	0.50	MST
M338*	0.26	0.54	0.48	0.58	0.59	0.48	0.50	1.00	0.00	0.62	0.67	0.35	0.20	0.72	0.50	MST
M233*	0.27	0.23	0.38	0.44	0.25	0.78	0.78	0.82	0.67	0.39	0.43	0.32	0.61	0.61	0.50	MST
M280	0.37	0.64	0.63	0.62	0.53	0.35	0.53	0.68	0.51	0.63	0.66	0.35	0.18	0.31	0.50	MST
M229	0.03	0.51	0.55	0.69	0.52	0.61	0.63	0.83	0.52	0.62	0.36	0.16	0.30	0.64	0.50	MST
M79	0.25	0.35	0.30	0.59	0.52	0.61	0.73	0.80	0.62	0.78	0.47	0.13	0.31	0.52	0.50	MST
M291	0.25	0.47	0.48	0.65	0.40	0.74	0.60	0.83	0.38	0.64	0.44	0.20	0.25	0.59	0.49	MST
M374	0.45	0.48	0.41	0.33	0.28	0.48	0.76	0.85	0.62	0.76	0.35	0.14	0.31	0.70	0.49	MST
M96	0.41	0.67	0.42	0.35	0.09	0.78	0.65	0.75	0.49	0.71	0.64	0.36	0.26	0.32	0.49	MST
M204	0.26	0.47	0.43	0.58	0.42	0.48	0.63	0.86	0.46	0.55	0.60	0.36	0.31	0.49	0.49	MST
M168*	0.45	0.62	0.28	0.15	0.17	0.54	0.75	0.78	0.69	0.69	0.65	0.29	0.41	0.40	0.49	MST
M106	0.29	0.47	0.64	0.42	0.35	0.48	0.65	0.54	0.71	0.47	0.66	0.54	0.47	0.18	0.49	MST
M92	0.11	0.68	0.78	0.27	0.18	0.57	0.61	0.47	0.69	0.45	0.71	0.87	0.33	0.14	0.49	MST
M175	0.57	0.37	0.45	0.22	0.36	0.39	0.64	0.87	0.47	0.76	0.81	0.38	0.22	0.32	0.49	MST
M379*	0.33	0.47	0.51	0.56	0.38	0.91	0.72	0.48	0.81	0.40	0.09	0.16	0.54	0.47	0.49	MST
M227	0.08	0.45	0.65	0.58	0.42	0.61	0.66	0.72	0.66	0.50	0.44	0.25	0.37	0.44	0.49	MST
M344	0.38	0.64	0.57	0.51	0.50	0.39	0.55	0.87	0.34	0.46	0.38	0.23	0.28	0.73	0.49	MST
M339	0.26	0.49	0.37	0.67	0.82	0.39	0.59	0.78	0.43	0.60	0.32	0.18	0.24	0.68	0.49	MST
M392	0.09	0.31	0.13	0.77	0.42	0.74	0.72	0.77	0.62	0.71	0.60	0.24	0.31	0.38	0.49	MST
M330*	0.27	0.60	0.65	0.31	0.32	0.39	0.66	0.77	0.53	0.65	0.58	0.39	0.29	0.38	0.49	MST
M401*	0.31	0.60	0.13	0.92	0.80	0.48	0.53	0.55	0.56	0.53	0.42	0.31	0.28	0.38	0.48	MST
M120	0.36	0.50	0.56	0.74	0.54	0.52	0.57	0.61	0.57	0.68	0.32	0.11	0.18	0.52	0.48	MST
M154	0.15	0.51	0.47	0.42	0.39	0.61	0.73	0.52	0.80	0.62	0.60	0.32	0.45	0.19	0.48	MST
M312	0.29	0.47	0.60	1.00	0.64	0.70	0.51	0.49	0.62	0.48	0.12	0.12	0.25	0.48	0.48	MST
M150	0.34	0.56	0.68	0.38	0.25	0.65	0.68	0.52	0.73	0.77	0.52	0.21	0.23	0.24	0.48	MST
M146	0.34	0.55	0.39	0.36	0.28	0.65	0.71	0.60	0.73	0.66	0.59	0.32	0.33	0.26	0.48	MST
M407	0.20	0.38	0.48	0.66	0.58	0.39	0.65	0.80	0.55	0.53	0.43	0.25	0.32	0.56	0.48	MST

M77	0.18	0.50	0.39	0.42	0.49	0.74	0.72	0.58	0.76	0.72	0.43	0.17	0.29	0.34	0.48	MST
M406	0.04	0.60	0.23	0.81	0.60	0.57	0.58	0.77	0.54	0.66	0.42	0.19	0.23	0.51	0.48	MST
M353*	0.35	0.52	0.40	0.42	0.41	0.48	0.61	0.90	0.39	0.58	0.36	0.19	0.29	0.84	0.48	MST
M195	0.26	0.57	0.44	0.31	0.14	0.70	0.57	0.62	0.65	0.50	0.70	0.66	0.43	0.19	0.48	MST
M216	0.07	0.60	0.38	0.31	0.22	0.61	0.67	0.75	0.58	0.44	0.80	0.64	0.43	0.24	0.48	MST
M356	0.34	0.75	0.59	0.62	0.44	0.30	0.63	0.72	0.60	0.55	0.08	0.12	0.29	0.69	0.48	MST
M11	0.27	0.45	0.49	0.20	0.28	0.48	0.71	0.95	0.36	0.76	0.48	0.15	0.31	0.82	0.48	MST
M103	0.44	0.71	0.54	0.37	0.26	0.48	0.51	0.83	0.36	0.62	0.65	0.36	0.18	0.40	0.48	MST
M59	0.10	0.71	0.81	0.47	0.23	0.78	0.70	0.20	0.91	0.75	0.43	0.16	0.32	0.14	0.48	MST
M55	0.22	0.45	0.46	0.61	0.71	0.65	0.60	0.56	0.60	0.53	0.37	0.24	0.28	0.40	0.48	MST
M271	0.30	0.41	0.55	0.30	0.15	0.65	0.75	0.37	0.71	0.42	0.71	0.56	0.53	0.25	0.48	MST
M269	0.20	0.41	0.48	0.41	0.34	0.43	0.63	0.65	0.58	0.55	0.80	0.73	0.30	0.14	0.48	MST
M367	0.14	0.43	0.38	0.42	0.85	0.30	0.57	0.84	0.43	0.61	0.68	0.38	0.23	0.39	0.48	MST
M207	0.19	0.50	0.15	0.43	0.43	0.43	0.73	0.84	0.54	0.59	0.59	0.31	0.39	0.50	0.47	MST
M206	0.38	0.32	0.34	0.33	0.34	0.35	0.67	0.58	0.75	0.43	0.88	0.75	0.40	0.08	0.47	MST
M69*	0.62	0.33	0.47	0.61	0.59	0.30	0.53	0.82	0.41	0.61	0.32	0.15	0.18	0.66	0.47	MST
M196	0.43	0.35	0.40	0.36	0.44	0.30	0.64	0.97	0.23	0.57	0.48	0.24	0.31	0.85	0.47	MST
M267	0.38	0.54	0.45	0.40	0.39	0.48	0.52	0.84	0.36	0.64	0.55	0.30	0.20	0.53	0.47	MST
M40	0.30	0.50	0.31	0.70	0.78	0.52	0.51	0.59	0.58	0.88	0.38	0.09	0.06	0.34	0.47	MST
M187	0.26	0.44	0.43	0.33	0.31	0.48	0.69	0.62	0.70	0.27	0.62	0.58	0.55	0.26	0.47	MST
M119	0.23	0.59	0.52	0.33	0.20	0.43	0.62	0.72	0.59	0.71	0.64	0.37	0.21	0.37	0.47	MST
M266	0.38	0.51	0.41	0.71	0.50	0.52	0.58	0.68	0.57	0.71	0.08	0.02	0.18	0.66	0.47	MST
M236	0.26	0.71	0.44	0.65	0.40	0.52	0.50	0.66	0.54	0.57	0.38	0.27	0.20	0.39	0.46	MST
M282	0.12	0.57	0.45	0.36	0.38	0.61	0.61	0.57	0.67	0.65	0.64	0.43	0.23	0.19	0.46	MST
M217	0.28	0.60	0.62	0.36	0.27	0.52	0.51	0.85	0.31	0.38	0.52	0.38	0.30	0.57	0.46	MST
M319*	0.14	0.72	0.36	0.50	0.56	0.35	0.48	0.74	0.41	0.50	0.69	0.53	0.21	0.28	0.46	MST
M109	0.41	0.79	1.00	0.30	0.19	0.39	0.39	0.80	0.26	0.59	0.42	0.23	0.13	0.56	0.46	MST
M63	0.19	0.74	0.75	0.27	0.11	0.65	0.66	0.42	0.78	0.60	0.46	0.31	0.30	0.22	0.46	MST
M298	0.20	0.39	0.71	0.45	0.36	0.61	0.56	0.40	0.74	0.50	0.65	0.40	0.34	0.15	0.46	MST
M181	0.18	0.48	0.45	0.59	0.48	0.65	0.46	0.71	0.40	0.61	0.48	0.40	0.14	0.41	0.46	MST
M46	0.10	0.62	0.75	0.60	0.56	0.57	0.53	0.55	0.62	0.82	0.12	0.03	0.09	0.48	0.46	MST
M4	0.42	0.49	0.55	0.30	0.32	0.52	0.53	0.64	0.58	0.71	0.58	0.29	0.24	0.27	0.46	MST
M38	0.18	0.50	0.51	0.63	0.50	0.48	0.46	0.66	0.43	0.47	0.59	0.40	0.31	0.30	0.46	MST
M400	0.21	0.42	0.79	0.58	0.40	0.17	0.49	0.83	0.35	0.48	0.57	0.38	0.25	0.49	0.46	MST
M240	0.27	0.43	0.49	0.28	0.35	0.54	0.60	0.74	0.59	0.61	0.53	0.25	0.27	0.42	0.46	MST
M242	0.41	0.71	0.72	0.30	0.15	0.48	0.46	0.67	0.52	0.38	0.50	0.35	0.37	0.35	0.45	MST
M348	0.23	0.59	0.54	0.54	0.51	0.48	0.62	0.64	0.61	0.73	0.03	0.05	0.21	0.60	0.45	MST
M378	0.48	0.38	0.63	0.73	0.70	0.39	0.45	0.59	0.58	0.46	0.08	0.11	0.20	0.55	0.45	MST
M247*	0.44	0.56	0.67	0.88	0.63	0.43	0.40	0.38	0.64	0.56	0.11	0.12	0.14	0.37	0.45	MST
M333	0.23	0.62	0.40	0.65	0.31	0.61	0.46	0.68	0.50	0.54	0.51	0.27	0.18	0.36	0.45	MST
M342	0.25	0.51	0.63	0.27	0.26	0.35	0.66	0.86	0.51	0.74	0.02	0.00	0.24	1.00	0.45	MST
M162	0.58	0.49	0.44	0.53	0.46	0.48	0.48	0.57	0.62	0.60	0.34	0.17	0.17	0.36	0.45	MST
M57	0.19	0.33	0.30	0.51	0.36	0.39	0.59	0.77	0.46	0.71	0.76	0.37	0.28	0.26	0.45	MST

M127	0.10	0.62	0.52	0.53	0.60	0.35	0.52	0.55	0.65	0.64	0.53	0.27	0.17	0.25	0.45	MST
M166*	0.25	0.31	0.59	0.53	0.60	0.43	0.55	0.67	0.55	0.49	0.38	0.23	0.26	0.42	0.45	MST
M389	0.14	0.55	0.22	0.55	0.46	0.61	0.55	0.70	0.48	0.55	0.40	0.23	0.22	0.58	0.45	MST
M307	0.52	0.29	0.46	0.38	0.20	0.48	0.58	0.68	0.61	0.49	0.37	0.31	0.29	0.58	0.45	MST
M211	0.10	0.86	0.33	0.37	0.45	0.48	0.49	0.71	0.47	0.62	0.57	0.28	0.16	0.35	0.44	MST
M156	0.06	0.48	0.64	0.52	0.17	0.78	0.59	0.47	0.68	0.62	0.46	0.23	0.24	0.25	0.44	MST
M385	0.53	0.33	0.23	0.64	0.42	0.61	0.58	0.58	0.66	0.71	0.15	0.04	0.17	0.53	0.44	MST
M176	0.32	0.74	0.47	0.43	0.29	0.43	0.49	0.61	0.57	0.59	0.47	0.24	0.19	0.32	0.44	MST
M278	0.30	0.43	0.43	0.71	0.51	0.61	0.49	0.59	0.54	0.42	0.12	0.16	0.25	0.60	0.44	MST
M299	0.20	0.50	0.53	0.34	0.19	0.48	0.61	0.75	0.54	0.71	0.40	0.13	0.20	0.57	0.44	MST
M104	0.11	0.63	0.36	0.34	0.32	0.48	0.50	0.52	0.55	0.65	0.83	0.66	0.16	0.07	0.44	MST
M19	0.12	0.36	0.30	0.47	0.42	0.48	0.62	0.74	0.60	1.00	0.46	0.03	0.06	0.46	0.44	SS
M238	0.34	0.48	0.31	0.31	0.28	0.61	0.52	0.87	0.32	0.68	0.37	0.13	0.15	0.71	0.44	SS
M248*	0.35	0.60	0.61	0.29	0.02	0.43	0.57	0.34	0.79	0.49	0.66	0.47	0.34	0.10	0.43	SS
M160	0.23	0.44	0.43	0.23	0.18	0.30	0.60	0.88	0.38	0.89	0.65	0.25	0.11	0.48	0.43	SS
M201	0.24	0.47	0.39	0.59	0.28	0.70	0.65	0.19	0.85	0.57	0.39	0.21	0.32	0.21	0.43	SS
M144	0.26	0.45	0.43	0.38	0.47	0.43	0.43	0.61	0.47	0.66	0.74	0.42	0.12	0.18	0.43	SS
M164	0.28	0.40	0.23	0.27	0.23	0.17	0.65	0.84	0.45	0.51	0.70	0.49	0.32	0.47	0.43	SS
M107	0.18	0.28	0.40	0.22	0.35	0.30	0.63	0.83	0.49	0.47	0.69	0.45	0.34	0.41	0.43	SS
M51	0.18	0.45	0.52	0.48	0.52	0.43	0.52	0.55	0.61	0.55	0.34	0.19	0.25	0.43	0.43	SS
M230	0.27	0.39	0.40	0.42	0.08	0.57	0.66	0.31	0.78	0.46	0.65	0.52	0.40	0.11	0.43	SS
M147	0.11	0.42	0.56	0.61	0.42	0.65	0.51	0.16	0.77	0.69	0.60	0.24	0.15	0.10	0.43	SS
M398	0.26	0.48	0.31	0.36	0.35	0.35	0.59	0.79	0.51	0.47	0.34	0.20	0.34	0.65	0.43	SS
M283	0.11	0.79	0.66	0.57	0.49	0.43	0.33	0.59	0.46	0.52	0.26	0.15	0.14	0.48	0.43	SS
M157	0.12	0.52	0.56	0.33	0.27	0.57	0.53	0.45	0.71	0.59	0.50	0.40	0.24	0.19	0.43	SS
M277	0.27	0.49	0.52	0.34	0.32	0.37	0.50	0.85	0.27	0.57	0.43	0.24	0.19	0.63	0.43	SS
M341	0.06	0.30	0.25	0.65	0.52	0.17	0.58	0.85	0.38	0.64	0.59	0.29	0.23	0.48	0.43	SS
M294*	0.18	0.56	0.45	0.49	0.32	0.48	0.49	0.69	0.47	0.73	0.37	0.12	0.11	0.50	0.43	SS
M169	0.21	0.65	0.50	0.24	0.30	0.26	0.44	0.76	0.43	0.81	0.71	0.25	0.10	0.31	0.43	SS
M405*	0.41	0.50	0.75	0.18	0.11	0.43	0.49	0.88	0.25	0.66	0.09	0.05	0.15	1.00	0.43	SS
M100*	0.25	0.42	0.51	0.26	0.16	0.39	0.71	0.71	0.71	0.68	0.05	0.04	0.33	0.74	0.43	SS
M252*	0.16	0.27	0.30	0.34	0.54	0.30	0.63	0.79	0.45	0.62	0.53	0.29	0.27	0.46	0.42	SS
M203*	0.11	0.68	0.12	0.05	0.08	0.30	0.55	0.74	0.47	0.52	1.00	0.98	0.25	0.09	0.42	SS
M384	0.47	0.53	0.37	0.20	0.02	0.48	0.62	0.58	0.61	0.39	0.56	0.40	0.36	0.33	0.42	SS
M66*	0.54	0.47	0.25	0.26	0.24	0.11	0.58	0.56	0.71	0.66	0.78	0.43	0.22	0.13	0.42	SS
M383	0.20	0.33	0.28	0.69	0.66	0.48	0.38	0.57	0.48	0.58	0.53	0.30	0.11	0.32	0.42	SS
M290	0.25	0.29	0.22	0.50	0.48	0.35	0.54	0.81	0.43	0.51	0.47	0.29	0.26	0.52	0.42	SS
M381	0.28	0.59	0.37	0.44	0.26	0.61	0.52	0.60	0.56	0.50	0.30	0.19	0.27	0.43	0.42	SS
M101	0.53	0.40	0.37	0.53	0.54	0.43	0.38	0.41	0.61	0.65	0.44	0.23	0.09	0.25	0.42	SS
M223	0.10	0.51	0.30	0.58	0.59	0.35	0.49	0.65	0.56	0.32	0.37	0.30	0.29	0.45	0.42	SS
M265	0.16	0.36	0.34	0.66	0.53	0.52	0.52	0.49	0.66	0.69	0.36	0.13	0.14	0.31	0.42	SS
M402*	0.26	0.53	0.42	0.18	0.18	0.48	0.39	0.88	0.16	0.52	0.81	0.56	0.16	0.33	0.42	SS
M324	0.29	0.45	0.50	0.19	0.12	0.61	0.69	0.49	0.79	0.89	0.24	0.00	0.18	0.40	0.42	SS

M331	0.19	0.60	0.25	0.66	0.54	0.26	0.48	0.64	0.55	0.54	0.29	0.18	0.19	0.47	0.42	SS
M246	0.19	0.25	0.79	0.21	0.27	0.26	0.57	0.45	0.72	0.57	0.70	0.53	0.23	0.09	0.42	SS
M288*	0.24	0.29	0.13	0.58	0.32	0.41	0.50	0.91	0.24	0.27	0.41	0.36	0.39	0.78	0.42	SS
M260*	0.37	0.39	0.06	0.47	0.40	0.43	0.63	0.72	0.56	0.34	0.05	0.14	0.41	0.83	0.42	SS
M133	0.18	0.68	0.52	0.40	0.42	0.30	0.42	0.54	0.59	0.52	0.50	0.27	0.19	0.28	0.42	SS
M138	0.28	0.40	0.16	0.49	0.22	0.30	0.64	0.80	0.55	0.66	0.21	0.11	0.27	0.73	0.42	SS
M243*	0.14	0.56	0.51	0.24	0.31	0.43	0.49	0.41	0.55	0.71	0.76	0.43	0.14	0.10	0.41	SS
M263	0.18	0.26	0.50	0.48	0.71	0.26	0.41	0.68	0.45	0.84	0.49	0.10	0.02	0.41	0.41	SS
M27	0.11	0.47	0.62	0.60	0.41	0.39	0.51	0.46	0.67	0.84	0.25	0.03	0.08	0.35	0.41	SS
M287	0.19	0.58	0.61	0.55	0.28	0.30	0.37	0.75	0.32	0.63	0.42	0.18	0.10	0.49	0.41	SS
M140	0.10	0.16	0.28	0.40	0.58	0.22	0.63	0.80	0.54	0.57	0.42	0.22	0.28	0.57	0.41	SS
M317*	0.30	0.54	0.29	0.38	0.51	0.04	0.52	0.64	0.60	0.35	0.52	0.41	0.33	0.32	0.41	SS
M35*	0.04	0.40	0.21	0.48	0.48	0.52	0.57	0.56	0.62	0.62	0.46	0.25	0.23	0.31	0.41	SS
M141	0.05	0.29	0.52	0.22	0.46	0.17	0.54	0.50	0.62	0.62	0.87	0.60	0.19	0.08	0.41	SS
M151	0.18	0.43	0.65	0.21	0.09	0.35	0.53	0.53	0.65	0.55	0.67	0.48	0.26	0.15	0.41	SS
M255	0.14	0.38	0.50	0.27	0.37	0.39	0.48	0.58	0.50	0.81	0.70	0.32	0.10	0.19	0.41	SS
M272	0.20	0.52	0.29	0.36	0.37	0.35	0.50	0.70	0.47	0.70	0.43	0.20	0.20	0.43	0.41	SS
M329	0.33	0.31	0.53	0.52	0.39	0.43	0.49	0.62	0.35	0.56	0.25	0.18	0.23	0.55	0.41	SS
M237*	0.39	0.43	0.41	0.35	0.28	0.52	0.44	0.48	0.56	0.64	0.51	0.31	0.19	0.21	0.41	SS
M33	0.36	0.44	0.47	0.54	0.63	0.17	0.35	0.62	0.47	0.51	0.40	0.24	0.13	0.37	0.41	SS
M325	0.32	0.44	0.31	0.34	0.34	0.09	0.53	0.69	0.51	0.46	0.55	0.34	0.40	0.35	0.41	SS
M89	0.18	0.58	0.40	0.40	0.26	0.39	0.61	0.42	0.62	0.54	0.35	0.29	0.34	0.29	0.41	SS
M357	0.07	0.48	0.16	0.62	0.58	0.22	0.46	0.63	0.47	0.31	0.58	0.56	0.26	0.27	0.40	SS
M74*	0.10	0.47	0.77	0.23	0.20	0.46	0.59	0.40	0.65	0.64	0.43	0.24	0.26	0.22	0.40	SS
M352	0.19	0.35	0.23	0.33	0.23	0.35	0.61	0.77	0.50	0.59	0.45	0.27	0.30	0.48	0.40	SS
M39	0.36	0.31	0.35	0.47	0.27	0.30	0.47	0.46	0.64	0.74	0.71	0.34	0.10	0.12	0.40	SS
M170	0.09	0.43	0.58	0.31	0.20	0.39	0.53	0.47	0.62	0.60	0.55	0.34	0.27	0.27	0.40	SS
M386	0.21	0.38	0.25	0.30	0.30	0.30	0.55	0.72	0.54	0.79	0.62	0.20	0.13	0.33	0.40	SS
M17	0.26	0.40	0.26	0.40	0.43	0.61	0.43	0.55	0.54	0.49	0.32	0.30	0.27	0.34	0.40	SS
M155	0.22	0.28	0.40	0.29	0.21	0.52	0.49	0.31	0.65	0.78	0.79	0.50	0.11	0.03	0.40	SS
M87	0.26	0.34	0.51	0.44	0.45	0.22	0.43	0.42	0.62	0.72	0.63	0.31	0.09	0.14	0.40	SS
M397	0.45	0.00	0.53	0.26	0.13	0.48	0.51	0.68	0.56	0.60	0.60	0.30	0.18	0.30	0.40	SS
M305	0.13	0.53	0.42	0.64	0.50	0.30	0.34	0.50	0.37	0.73	0.48	0.20	0.08	0.33	0.40	SS
M337	0.19	0.41	0.30	0.61	0.39	0.30	0.45	0.60	0.49	0.62	0.42	0.24	0.16	0.34	0.40	SS
M403	0.30	0.43	0.39	0.20	0.33	0.43	0.38	0.68	0.39	0.61	0.60	0.32	0.14	0.32	0.39	SS
M152	0.12	0.53	0.46	0.17	0.32	0.43	0.49	0.43	0.61	0.75	0.64	0.29	0.11	0.14	0.39	SS
M286	0.08	0.39	0.00	0.19	0.37	0.26	0.69	0.87	0.49	0.67	0.43	0.14	0.26	0.64	0.39	SS
M304	0.13	0.40	0.50	0.48	0.34	0.48	0.41	0.63	0.29	0.78	0.37	0.13	0.07	0.47	0.39	SS
Mxg	0.09	0.57	0.20	0.28	0.24	0.52	0.56	0.48	0.67	0.73	0.45	0.14	0.19	0.35	0.39	SS
M20*	0.14	0.26	0.45	0.37	0.49	0.13	0.47	0.50	0.62	0.69	0.67	0.35	0.14	0.19	0.39	SS
M159*	0.24	0.18	0.30	0.10	0.28	0.22	0.57	0.91	0.27	0.93	0.68	0.21	0.10	0.47	0.39	SS
M232	0.22	0.32	0.13	0.35	0.27	0.30	0.49	0.73	0.41	0.68	0.70	0.37	0.18	0.28	0.39	SS
M189*	0.13	0.41	0.25	0.62	0.68	0.26	0.35	0.59	0.51	0.53	0.38	0.20	0.13	0.39	0.39	SS

M258*	0.06	0.68	0.89	0.29	0.10	0.35	0.41	0.63	0.50	0.40	0.20	0.19	0.23	0.49	0.39	SS
M375	0.10	0.32	0.26	0.42	0.57	0.30	0.43	0.87	0.20	0.45	0.35	0.24	0.19	0.72	0.39	SS
M281	0.28	0.49	0.45	0.41	0.33	0.26	0.41	0.76	0.33	0.50	0.16	0.15	0.17	0.70	0.39	SS
M191	0.12	0.64	0.31	0.30	0.27	0.26	0.41	0.41	0.60	0.60	0.77	0.46	0.13	0.11	0.38	SS
M318*	0.25	0.48	0.47	0.35	0.35	0.17	0.39	0.71	0.25	0.49	0.49	0.28	0.15	0.53	0.38	SS
M102	0.10	0.32	0.39	0.44	0.45	0.39	0.48	0.67	0.49	0.76	0.16	0.04	0.09	0.59	0.38	SS
M7	0.28	0.47	0.56	0.32	0.34	0.17	0.38	0.69	0.40	0.63	0.36	0.14	0.10	0.50	0.38	SS
M13	0.12	0.53	0.34	0.37	0.52	0.26	0.22	0.72	0.21	0.48	0.74	0.51	0.09	0.26	0.38	SS
M99	0.06	0.52	0.41	0.41	0.46	0.35	0.39	0.55	0.53	0.67	0.31	0.15	0.10	0.41	0.38	SS
M380	0.28	0.40	0.34	0.34	0.18	0.57	0.47	0.38	0.61	0.58	0.48	0.26	0.24	0.21	0.38	SS
M126	0.26	0.38	0.30	0.48	0.75	0.26	0.21	0.62	0.34	0.76	0.45	0.17	0.00	0.34	0.38	SS
M241	0.19	0.34	0.25	0.31	0.25	0.48	0.43	0.43	0.64	0.57	0.64	0.49	0.17	0.13	0.38	SS
M18	0.34	0.44	0.46	0.23	0.00	0.43	0.50	0.27	0.69	0.65	0.57	0.28	0.31	0.12	0.38	SS
M190	0.22	0.31	0.32	0.50	0.57	0.13	0.49	0.55	0.57	0.47	0.11	0.26	0.24	0.49	0.38	SS
M167*	0.02	0.74	0.18	0.21	0.25	0.43	0.48	0.20	0.67	0.39	0.70	0.60	0.31	0.06	0.38	SS
M185	0.09	0.39	0.25	0.44	0.42	0.22	0.56	0.45	0.68	0.62	0.42	0.23	0.20	0.27	0.37	SS
M284	0.33	0.40	0.39	0.26	0.20	0.35	0.50	0.55	0.60	0.64	0.36	0.17	0.16	0.34	0.37	SS
M30	0.15	0.38	0.44	0.14	0.08	0.52	0.60	0.31	0.81	0.63	0.46	0.28	0.28	0.16	0.37	SS
M21	0.06	0.24	0.26	0.46	0.46	0.35	0.44	0.69	0.47	0.57	0.33	0.19	0.15	0.53	0.37	SS
M355	0.34	0.41	0.36	0.36	0.38	0.39	0.40	0.50	0.42	0.71	0.31	0.17	0.12	0.35	0.37	SS
M137	0.24	0.27	0.40	0.36	0.48	0.22	0.35	0.49	0.50	0.56	0.59	0.45	0.11	0.19	0.37	SS
M261	0.39	0.41	0.23	0.41	0.28	0.26	0.41	0.64	0.28	0.67	0.43	0.19	0.11	0.47	0.37	SS
M47	0.04	0.26	0.07	0.29	0.34	0.26	0.54	0.85	0.29	0.62	0.64	0.33	0.20	0.44	0.37	SS
M254	0.13	0.54	0.75	0.39	0.23	0.22	0.38	0.49	0.52	0.56	0.22	0.18	0.12	0.41	0.37	SS
M214	0.31	0.52	0.37	0.47	0.42	0.26	0.17	0.61	0.31	0.58	0.42	0.21	0.05	0.42	0.37	SS
M210	0.02	0.24	0.30	0.41	0.41	0.26	0.47	0.65	0.41	0.56	0.48	0.24	0.18	0.46	0.36	SS
M270	0.19	0.32	0.14	0.54	0.57	0.30	0.52	0.44	0.71	0.71	0.06	0.05	0.15	0.39	0.36	SS
M301	0.16	0.30	0.25	0.53	0.53	0.39	0.51	0.00	0.77	0.60	0.51	0.24	0.18	0.12	0.36	SS
M172	0.23	0.43	0.38	0.23	0.26	0.13	0.33	0.70	0.27	0.62	0.66	0.39	0.09	0.35	0.36	SS
M251*	0.30	0.28	0.47	0.35	0.23	0.30	0.55	0.25	0.81	0.57	0.33	0.17	0.22	0.22	0.36	SS
M234*	0.09	0.35	0.40	0.29	0.27	0.30	0.37	0.66	0.41	0.69	0.54	0.22	0.08	0.36	0.36	SS
M250	0.29	0.43	0.44	0.45	0.44	0.26	0.24	0.61	0.22	0.64	0.20	0.08	0.06	0.66	0.36	SS
M295	0.11	0.48	0.29	0.39	0.33	0.22	0.42	0.38	0.65	0.66	0.44	0.29	0.12	0.23	0.36	SS
M326	0.17	0.36	0.27	0.37	0.33	0.57	0.27	0.57	0.48	0.58	0.32	0.21	0.11	0.40	0.36	SS
M5	0.11	0.12	0.07	0.20	0.24	0.09	0.55	0.71	0.53	0.38	0.72	0.67	0.37	0.22	0.36	SS
M212	0.14	0.33	0.19	0.41	0.29	0.41	0.38	0.55	0.54	0.42	0.52	0.36	0.20	0.25	0.36	SS
M85	0.09	0.31	0.09	0.32	0.44	0.22	0.44	0.76	0.32	0.58	0.52	0.39	0.13	0.37	0.36	SS
M292	0.09	0.27	0.20	0.29	0.54	0.35	0.49	0.50	0.48	0.61	0.37	0.22	0.19	0.37	0.36	SS
M347	0.00	0.32	0.48	0.31	0.51	0.22	0.31	0.54	0.48	0.38	0.61	0.42	0.16	0.22	0.35	SS
M158	0.12	0.50	0.46	0.22	0.34	0.17	0.37	0.59	0.48	0.78	0.32	0.14	0.06	0.41	0.35	SS
M131	0.15	0.32	0.30	0.29	0.16	0.30	0.37	0.76	0.33	0.39	0.54	0.39	0.22	0.43	0.35	SS
M224	0.28	0.30	0.47	0.29	0.24	0.39	0.46	0.27	0.77	0.54	0.27	0.19	0.22	0.23	0.35	SS
M349	0.37	0.51	0.45	0.57	0.30	0.43	0.27	0.32	0.59	0.51	0.00	0.09	0.09	0.38	0.35	HSS

M410	0.30	0.19	0.25	0.21	0.49	0.04	0.36	0.75	0.36	0.44	0.50	0.32	0.20	0.48	0.35	HSS
M268	0.25	0.08	0.40	0.35	0.29	0.35	0.43	0.45	0.61	0.47	0.42	0.34	0.19	0.24	0.35	HSS
M358*	0.23	0.49	0.35	0.49	0.20	0.48	0.28	0.40	0.61	0.35	0.16	0.21	0.18	0.36	0.34	HSS
M334	0.28	0.44	0.40	0.39	0.24	0.26	0.34	0.54	0.53	0.34	0.18	0.19	0.18	0.45	0.34	HSS
M73	0.34	0.46	0.33	0.23	0.30	0.39	0.26	0.43	0.49	0.49	0.35	0.29	0.14	0.27	0.34	HSS
M360	0.15	0.20	0.05	0.51	0.51	0.09	0.50	0.69	0.47	0.47	0.10	0.11	0.23	0.68	0.34	HSS
M108*	0.13	0.24	0.39	0.44	0.46	0.30	0.32	0.56	0.45	0.61	0.09	0.05	0.08	0.58	0.33	HSS
M32*	0.09	0.42	0.22	0.11	0.14	0.26	0.39	0.38	0.69	0.79	0.68	0.24	0.10	0.14	0.33	HSS
M25	0.10	0.33	0.15	0.30	0.21	0.48	0.36	0.64	0.41	0.76	0.16	0.05	0.08	0.57	0.33	HSS
M10	0.03	0.24	0.16	0.50	0.37	0.00	0.36	0.70	0.40	0.46	0.50	0.29	0.14	0.39	0.32	HSS
M289	0.31	0.27	0.48	0.39	0.40	0.13	0.32	0.29	0.68	0.47	0.23	0.14	0.13	0.26	0.32	HSS
M336	0.07	0.25	0.26	0.11	0.28	0.22	0.35	0.54	0.47	0.61	0.65	0.34	0.10	0.20	0.32	HSS
M411*	0.00	0.28	0.31	0.29	0.25	0.35	0.26	0.83	0.06	0.40	0.38	0.28	0.13	0.62	0.32	HSS
M198	0.10	0.36	0.20	0.32	0.31	0.30	0.27	0.50	0.43	0.53	0.28	0.28	0.12	0.34	0.31	HSS
M123*	0.08	0.09	0.06	0.00	0.24	0.00	0.37	0.44	0.55	0.55	0.95	0.78	0.12	0.00	0.30	HSS
M161	0.13	0.24	0.26	0.25	0.15	0.26	0.20	0.29	0.60	0.50	0.61	0.48	0.09	0.09	0.30	HSS
M76	0.31	0.38	0.33	0.19	0.29	0.17	0.13	0.44	0.37	0.60	0.37	0.30	0.02	0.24	0.30	HSS
M88*	0.20	0.47	0.25	0.22	0.19	0.17	0.00	0.49	0.26	0.56	0.63	0.37	0.02	0.17	0.29	HSS
M142	0.01	0.19	0.05	0.17	0.20	0.13	0.26	0.38	0.59	0.44	0.42	0.26	0.13	0.22	0.25	HSS
M122*	0.12	0.18	0.11	0.21	0.29	0.09	0.05	0.32	0.52	0.00	0.49	0.65	0.16	0.16	0.24	HSS

*64 genotypes of mini-core collection developed using the Core Hunter algorithm.

MFV: membership function value; HST: highly salt tolerant, ST: salt tolerant, MST: moderately salt tolerant, SS: salt sensitive, HSS: highly salt sensitive.

RGR: salt-tolerance index of shoot growth rate, RNIL: salt-tolerance index of leaves increased number, RLER: salt-tolerance index of leaf expansion rate, Sen: leaf senescence scale, SWC: shoot water content, RWC: root water content, SNC: shoot Na^+ concentration, RNC: root Na^+ concentration, SN/RN: the ratio of shoot Na^+ concentration to root Na^+ concentration, SKC: shoot K^+ concentration, RKC: root K^+ concentration, SK/RK: the ratio of shoot K^+ concentration to root K^+ concentration, SK/N: the ratio of shoot K^+ concentration to shoot Na^+ concentration, RK/N: the ratio of root K^+ concentration to root Na^+ concentration.

Table S6. Comparison of all traits of genotypes of different salt tolerance categories in *M. sacchariflorus* and *M. lutarioriparius*.

Category	RGR	RNIL	RLER	SWC (%)	RWC (%)	Sen	SNC (mg/g)	RNC (mg/g)	SN/RN	SKC (mg/g)	RKC (mg/g)	SK/RK	SK/N	RK/N
HST	0.93	0.61	0.87	15.43	9.26	4.41	72.52	40.31	1.96	76.31	17.97	4.74	1.12	0.46
ST	0.72	0.62	0.72	17.64	12.43	5.38	98.81	43.30	2.57	66.83	23.82	3.22	0.74	0.60
MST	0.56	0.52	0.68	15.97	10.65	5.87	113.95	44.56	2.92	67.54	25.60	3.01	0.63	0.63
SS	0.40	0.41	0.55	12.96	9.65	6.90	139.98	49.21	3.30	68.27	26.08	2.92	0.52	0.58
HSS	0.35	0.31	0.44	11.45	8.48	7.31	176.60	54.89	3.53	74.73	29.24	2.96	0.44	0.56

HST: highly salt tolerant, ST: salt tolerant, MST: moderately salt tolerant, SS: salt sensitive, HSS: highly salt sensitive.

RGR: salt-tolerance index of shoot growth rate, RNIL: salt-tolerance index of leaves increased number, RLER: salt-tolerance index of leaf expansion rate, Sen: leaf senescence scale, SWC: shoot water content, RWC: root water content, SNC: shoot Na^+ concentration, RNC: root Na^+ concentration, SN/RN: the ratio of shoot Na^+ concentration to root Na^+ concentration, SKC: shoot K^+ concentration, RKC: root K^+ concentration, SK/RK: the ratio of shoot K^+ concentration to root K^+ concentration, SK/N: the ratio of shoot K^+ concentration to shoot Na^+ concentration, RK/N: the ratio of root K^+ concentration to root Na^+ concentration.
